# Potential plastic biodegradation in lakes worldwide

**DOI:** 10.1016/j.xinn.2026.101338

**Published:** 2026-03-03

**Authors:** Qi Zhang, Zhenyan Zhang, Ziyao Zhang, Guoyan Qin, Mingkang Jin, Bingfeng Chen, Yitian Yu, Tingzhang Wang, Meixia Wang, Tao Lu, Dong Zhu, Li Cui, Haifeng Qian, Matthias C. Rillig, Yong-Guan Zhu

**Affiliations:** 1 Institute for Advanced Study, Shaoxing University, Shaoxing 312000, China; 2 College of Environment, Zhejiang University of Technology, Hangzhou 310032, China; 3 Key Laboratory of Urban Environment and Health, Institute of Urban Environment, Chinese Academy of Sciences, Xiamen 361021, China; 4 Institute of Biology, Freie Universität Berlin, Berlin, Germany; 5 State Key Laboratory of Urban and Regional Ecology, Research Center for Eco-environmental Sciences, Chinese Academy of Sciences, Beijing 100085, China; 6 Key Laboratory of Microbial Technology and Bioinformatics of Zhejiang Province, Hangzhou 310012, China

**Keywords:** plastic pollution, lake microbiome, plastic-degrading bacteria, machine learning, plastisphere

## Abstract

Plastic pollution is ubiquitous, yet the biodegradation of plastic waste remains poorly understood due to limited knowledge of microbial plastic degradation potential. Here, we demonstrate that plastic waste shapes the global distribution of plastic-degrading potential across 182,661 lakes worldwide using integrated metagenomic and machine learning analyses and identify a tipping point (≥7.44 particles/m^3^) for effective *in situ* bioremediation. We constructed, for the first time, a catalog of candidate plastic-degrading bacteria, including 15,715 nonredundant enzyme homologs and 4,856 metagenome-assembled genomes. To facilitate future applications, we developed a computational approach to categorizing candidate plastic-degrading bacteria according to their degradation potential, ecological risk, environmental adaptation, and competition capacity. Furthermore, we customized eight template culture media based on the growth factor biosynthesis profiles of high-priority candidate plastic-degrading bacteria. Using these media, we successfully enriched the plastic-degrading microbial communities and isolated a high-priority strain, *Serratia ficaria* HfyG-1, from Xiazhu Lake, which harbors a wide variety of previously uncharacterized putative degrading enzymes that effectively degrade polylactic acid and polyethylene terephthalate. Our study provides a molecular resource for the bioremediation of plastic-polluted environments worldwide and highlights a proof-of-concept framework for identifying, investigating, and exploiting unknown functional microorganisms for practical applications.

## Public summary

• Plastic waste contributes to the geographical distribution of global lake plastic-degrading potential.
• We develop a computational framework to assess environmental application potential of plastic degraders.
• Most plastic-degrading bacteria lack complete essential growth factor pathways.
• We provide a proof-of-concept framework to identify and harness novel plastic degraders.

## Graphical abstract

**Figure:**
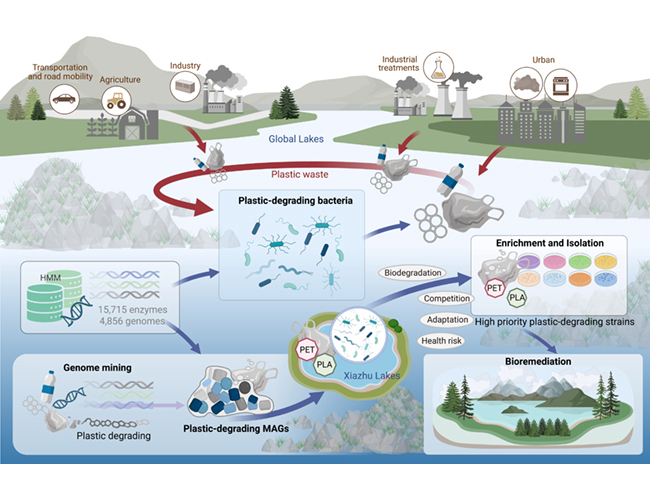

## Introduction

Plastic pollutants have emerged as one of the greatest challenges of our time.^1^^,^^2^ While most attention has focused on the ocean as a terminal aquatic sink,^3^ freshwater ecosystems have been comparatively neglected. A recent standardized survey across 38 lakes revealed that plastic concentrations in some lakes can exceed those in oceanic gyres.^4^ Owing to their limited water exchange and low oxygen levels, lakes have reduced self-purifying capacity and therefore accumulate various pollutants that are adsorbed on the surface of plastic debris.^5^ Lakes also provide more direct exposure pathways for plastic debris to humans than many other ecosystems do because lakes are, for example, used for irrigation, drinking water, or recreation.^6^ A growing body of evidence indicates that plastic contamination profoundly alters microbial community composition and ecological functions in freshwater systems.^7^^,^^8^^,^^9^^,^^10^ Such continuous restructuring of lake microbiomes leads to changes in nutrient cycling, biofilm dynamics, and pathogen transmission, thereby threatening ecosystem stability and human health. Notably, persistent plastic contamination drives a bidirectional interaction between pollutants and the microbiota: while plastics reshape the microbial community structure, microorganisms adapt by evolving and enriching plastic-degrading traits. However, the true breadth and efficiency of microbial plastic degradation in lakes remain poorly understood.

Microbial degradation delivers dual environmental benefits: mitigating pervasive microplastic pollution while catalyzing a circular economy through waste upcycling.^11^ Environmental microorganisms, which are chronically exposed to plastic pollution, have evolved unique enzymatic degradation systems that represent promising targets for enzyme discovery. Yoshida et al. first reported *Ideonella sakaiensis*—a polyethylene terephthalate (PET)-degrading bacterium—and its secreted enzyme PETase, which depolymerizes PET at 30°C, unveiling the metabolic potential of environmental microbes toward plastics.^12^ Subsequent efforts have established curated repositories of validated plastic-degrading enzymes, including the PAZy^13^ and PlasticsDB.^14^ Nevertheless, cultivation-dependent approaches create a bottleneck for obtaining reliable insights into plastic degraders, including plastic-degrading microorganisms and their associated enzymes, thereby limiting the discovery of novel functional elements.^15^ Meanwhile, analyses focused on individual locations limits our knowledge of the distribution of plastic degraders across lakes worldwide, as they vary as a function of various environmental and anthropogenetic factors.^15^ Moreover, because of the wide variety of forms and types of plastics in lakes, the identification and screening of specific plastic degraders is particularly challenging.^15^ Although recent studies have attempted to predict candidate plastic degraders on the basis of metagenomes from different habitats, their accuracy is limited by sequencing noise, coverage depth, and fragment length.^16^^,^^17^ Genome-resolved metagenomics can be used to construct composite genomes from microbial populations,^18^ providing reference genomes for uncultivated taxa and revealing enormous amounts of microbial dark matter.^19^ Therefore, it is essential to construct a catalog that represents plastic-degrading bacteria and their related enzymes in lakes worldwide.

The central challenge confronting the environmental deployment of plastic-degrading bacteria resides in their limited capacity for sustained surface colonization and persistence on nutrient-depauperate plastic substrates.^20^ Determining the environmental adaptability and competitiveness of plastic-degrading bacteria on a large scale is extremely challenging. Moreover, the ecological risk of the addition of plastic-degrading bacteria to the environment cannot be ignored; otherwise, unintended consequences may arise. Numerous studies have shown that plastic-degrading bacteria are potential pathogens or harbor ecological risk factors such as antibiotic resistance genes and virulence factors.^21^^,^^22^^,^^23^ Some known plastic-degrading bacteria are potential pathogens, such as *Pseudomonas aeruginosa*,^24^ *Klebsiella pneumoniae*,^25^ and *Burkholderia cepacian*.^26^ However, this issue has not yet received sufficient attention.

In this study, we systematically investigated the degradation potential, distribution, ecological risks, and metabolic functions of candidate plastic-degrading bacteria across lakes worldwide. We first applied hidden Markov models (HMMs) and machine learning to delineate the distribution and key environmental factors of plastic degradation potential in 182,661 lakes worldwide. Next, we constructed a metagenome-assembled genome (MAG) catalog of microbes with plastic-degrading potential in lakes worldwide and resolved their phylogenetic relationships. In addition, we developed a computational framework to quantitatively categorize candidate microbes according to their capacity for biodegradation, health risk, environmental adaptation, and resource competition. Additionally, we constructed biosynthetic profiles of essential growth factors in candidate plastic-degrading bacteria and designed customized culture media specifically to enrich these microorganisms. On the basis of these media, we enriched and isolated plastic-degrading bacterial strains from Xiazhu Lake (Huzhou, China) and rigorously verified their degradation abilities. Overall, we established a general workflow for identifying, investigating, and exploiting novel candidate plastic-degrading bacteria for the biodegradation and bioremediation of plastic waste in lakes worldwide.

## Materials and methods

Detailed descriptions of datasets, computational pipelines, experimental procedures, and statistical analyses are provided in the methods in the supplemental information.

### Metagenomic datasets

Public freshwater lake metagenomes were retrieved from the European Nucleotide Archive and National Center for Biotechnology Information (NCBI) Sequence Read Archive (metadata as of January 16, 2023) using the keywords “Lake,” “Metagenome,” and “Freshwater.” To minimize sampling bias, we excluded pond samples, non-water-column samples (e.g., sediments or aquatic organisms), lakes adjacent to point-source pollution or extreme environments, samples with inaccurate coordinates, and datasets lacking paired-end FASTQ reads or with mean read lengths below 100 bp. After filtering, 715 metagenomes from 95 lakes across 5 continents and 19 countries were retained.

### Metagenomic assemblies and reconstruction of lake plastic-degrading MAGs

Metagenomic assemblies were generated using MEGAHIT within the MetaWRAP pipeline.^27^ Reads were mapped to contigs with Bowtie2,^28^ and resulting alignments were processed using SAMtools.^29^ MAGs were reconstructed using a consensus binning strategy combining CONCOCT,^30^ MaxBin2,^31^ and MetaBAT2.^32^ MAG quality was evaluated with CheckM,^33^ retaining bins with ≥50% completeness and <10% contamination. To reduce functional false positives, downstream analyses were restricted to contigs longer than 10 kb and with taxonomic assignments consistent with their parent MAGs. Dereplication at 95% average nucleotide identity yielded 4,856 lake plastic-degrading MAGs (LPDMAGs), clustered into 1,370 species-level bins.

### Enzyme dataset and construction of HMMs

A curated reference set of 184 experimentally validated plastic-degrading enzymes spanning 10 plastic types was compiled from published literature and databases.^13^^,^^14^ These enzymes were grouped by polymer class and used to construct HMMs.^34^ To expand sequence diversity, homologous sequences were retrieved from the UniProt TrEMBL database, dereplicated, aligned, and used to build 413 HMM profiles. Predicted proteins from all metagenomes were scanned using hmmsearch, retaining only the best hit per gene. Candidate hits were further validated using the machine learning-based CLEAN framework to predict enzyme commission numbers, ensuring functional consistency with known plastic-degrading activities.^35^ This combined HMM-machine learning pipeline identified 45,154 nonredundant enzyme homologs across all lake metagenomes.

### Functional annotation of LPDMAGs

Antibiotic resistance genes were annotated using the Comprehensive Antibiotic Resistance Database with RGI. Virulence- and pathogenicity-related genes were identified using the PHI-base (www.phi-base.org/) and Virulence Factor Database of Pathogenic Bacteria databases (mgc.ac.cn). To characterize ecological functionality, LPDMAGs were annotated for carbon-, nitrogen-, phosphorus-, and sulfur-cycling potential using a curated integration of NCycDB, SCycDB, QMEC, and GeoChip databases.^36^^,^^37^^,^^38^^,^^39^ These annotations provided the basis for assessing metabolic versatility, ecological adaptation, and potential risk.

### Reconstruction of growth factor biosynthesis pathways

To evaluate nutritional independence, biosynthesis pathways for 41 essential growth factors were manually reconstructed, encompassing 73 pathways and 469 Kyoto Encyclopedia of Genes and Genomes orthologs. Nearly complete LPDMAGs were annotated against the Kyoto Encyclopedia of Genes and Genomes database to determine pathway completeness, accounting for alternative routes and required intermediates. This analysis quantified the extent to which candidate plastic degraders rely on external nutrient supplementation.

### Priority index for candidate plastic-degrading bacteria

We developed a computational framework to rank candidate plastic-degrading bacteria by integrating plastic-degrading potential, ecological risk, environmental adaptation capacity, and competitive capacity. Plastic-degrading potential was defined as the abundance of enzyme homologs per MAG. Adaptation and competitive capacities were estimated from functional traits related to stress tolerance, biofilm formation,^40^ and elemental cycling. Ecological risk was quantified based on pathogenicity, virulence, and antibiotic resistance profiles.^41^^,^^42^ These components were combined into a priority index, emphasizing high degradation potential and minimal ecological risk. Candidates were classified into four priority tiers using unsupervised clustering.

### Enrichment, isolation, and genomic characterization

Plastic colonization experiments were conducted in Xiazhu Lake (Huzhou, China) using polylactic acid (PLA) and PET substrates for *in situ* enrichment. Plastisphere communities were collected and transferred into mineral salt media supplemented with tailored growth factors. After long-term incubation, bacterial isolates were obtained through serial dilution and plating. A representative high-priority isolate, *Serratia ficaria* HfyG-1, was subjected to whole-genome sequencing, assembly, and annotation following standard quality control and functional annotation pipelines.

### Verification test of plastic degradation

Plastic degradation assays were performed using enriched communities and isolated strains incubated with PLA or PET as the sole carbon source. Degradation efficiency was assessed by mass loss measurements and surface characterization using scanning electron microscopy, atomic force microscopy, and Fourier transform infrared (FTIR) spectroscopy.

### Statistical analysis and visualization

Statistical analyses were performed using nonparametric tests and multivariate ordination methods as described in the main text. Data visualization and phylogenetic analyses were conducted using standard R, Python, and graphical software tools.

## Results

### Geographical distribution of microbial plastic-degrading potential in lakes

First, we compiled a catalog of 15,715 nonredundant candidate enzyme homologs with the potential to degrade 10 different plastics. We then characterized the distribution of microbial plastic-degrading potential on the basis of gene abundance across 715 metagenomes derived from 95 lakes (Figure S1; Table S1). Using HMMs integrated with machine learning, we identified a total of 45,154 putative plastic-degrading enzyme hits across all samples, spanning 10 representative polymer types (Table S2), and the pattern of plastic-degrading potential differed significantly across countries (Adonis analysis, *R*^2^ = 0.21, *p* < 0.001; Figure S2A), especially among lakes (Adonis analysis, *R*^2^ = 0.49, *p* < 0.001). Pairwise Adonis analysis based on Bray-Curtis dissimilarity revealed a different distribution of plastic-degrading potential among almost all the lakes (*p* < 0.001; Figure S2B), indicating a unique lake landscape with plastic-degrading potential.

**Figure 1:**
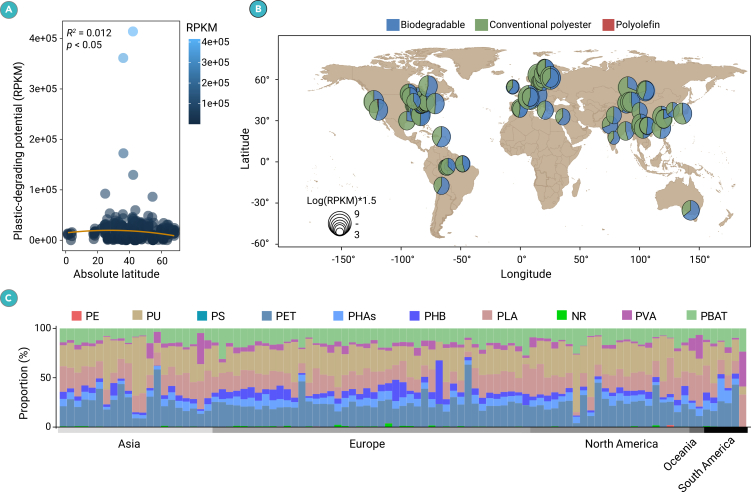
Composition and global distribution of microbial plastic-degrading potential in lakes (A) Total plastic-degrading potential is higher in mid-latitude lakes. The blue color gradient indicates the abundance of putative plastic-degrading enzyme-encoding genes. (B) Distribution of microbial plastic-degrading potential across 95 lakes. Blue, green, and red denote biodegradable, polyolefin, and conventional polyester plastics, respectively. Circle size represents the abundance of putative plastic-degrading enzyme-encoding genes [log(RPKM) × 1.5]. (C) Composition of putative plastic-degrading enzyme-encoding genes across 95 lakes spanning Asia, Europe, North America, Oceania, and South America, covering major plastic types, including PVA (polyvinyl alcohol), PHAs (polyhydroxyalkanoates), PHB (polyhydroxybutyrate), PU (polyurethane), PET (polyethylene terephthalate), PBAT (polybutylene adipate terephthalate), PS (polystyrene), PLA (polylactic acid), PE (polyethylene), and NR (natural rubber). RPKM, reads per kilobase per million mapped reads.

The total plastic-degrading potential was higher in mid-latitude lakes (*R*^2^ = 0.012, *p* < 0.05; Figure 1A), reflecting the combined influence of moderate climatic conditions and higher anthropogenic plastic inputs that favor microbial adaptation and enzymatic activity. The proportion of degrading potential for biodegradable, polyolefin, and conventional polyester plastics varied geographically (Figure 1B) due to differences in abundance of putative plastic-degrading enzyme-encoding genes across 95 lakes spanning 19 countries and 5 continents (Figures 1C, S3, and S4). We found that the microbial degradation potential varied substantially among the different plastic types across all the lake metagenomes (Figure S5). The genes encoding putative enzymes for PHA, PET, and polyurethane degradation were the most enriched, whereas those associated with polyolefin degradation showed the lowest abundance. Overall, the degradation potential of biodegradable and conventional polyester plastics was comparable and markedly higher than that of polyolefins, indicating that ester-linked polymers are more readily metabolized by lake microbial communities.

### Plastic pollution contributes to plastic-degrading potential in lake microbiomes

**Figure 2:**
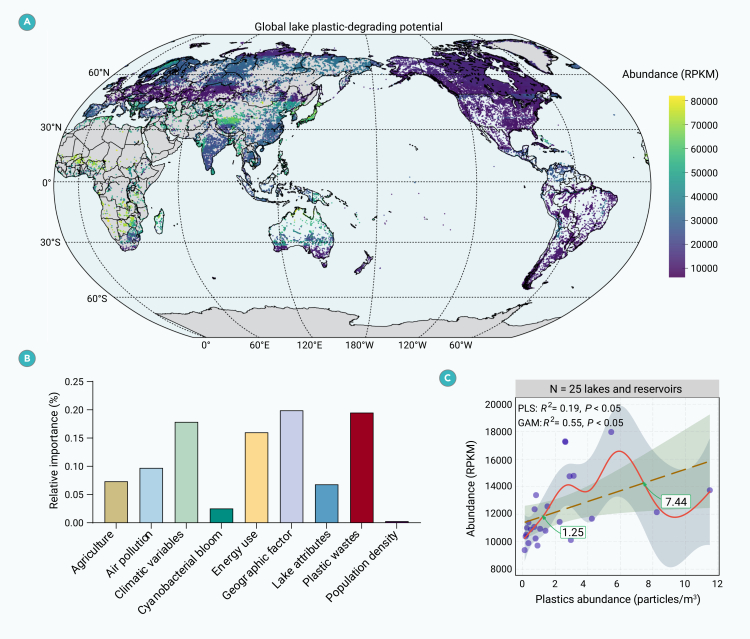
Global mapping of microbial plastic-degrading potential in lakes (A) Predicted plastic-degrading potential across 182,661 lakes worldwide (>1 km^2^). The color gradient from yellow to purple indicates the abundance of putative plastic-degrading enzyme-encoding genes [log(RPKM)], from high to low. (B) Relative importance of geographical variables highlights plastic pollution as the dominant driver shaping microbial plastic-degrading potential. (C) Plastic abundance (particles m^−3^) is positively associated with microbial plastic-degrading potential in lakes. A threshold (≥7.44 particles m^−3^) indicates a tipping point beyond which ecological disruption is influenced by factors exceeding simple resource availability. RPKM, reads per kilobase per million mapped reads; GAM, generalized additive model; PLS, partial least squares.

To examine the geographical mechanisms that drive the spatial patterns of plastic-degrading potential in lake microbiomes, we integrated 37 environmental and anthropogenic predictors constraints to map the microbial plastic degradation potential of 182,661 lakes worldwide (>1 km^2^) via machine learning (Figure 2A; Table S3). Among these predictors, national-level plastic waste generation and management statistics were used as proxies for contamination pressure, providing a large-scale environmental context rather than lake-specific pollution levels. The Random Forest model exhibited the best predictive performance, achieving the highest accuracy (*R*^2^ = 0.73) and the lowest root mean-square error (6605.01) among all the tested algorithms (Figure S6A), and residual analysis confirmed its robustness, with residuals tightly centered around zero (Figures S6B–S6D). In addition to geographic variation, plastic-related factors, especially national waste imports per capita, and climatic variables, such as moisture index seasonality and precipitation in the coldest quarter, were the strongest predictors of microbial plastic degradation potential (Figures 2B and S6E). These findings underscore the interplay between anthropogenic inputs and climate: plastic waste defines exposure intensity, whereas water balance and seasonality regulate microbial metabolism, jointly shaping the ecological niches of candidate plastic-degrading bacteria.

To further substantiate these model-derived patterns, empirical monitoring data of plastic abundance (particles/m^3^) from 25 lakes spanning 21 countries and 5 continents were obtained following the standardized protocol of Nava et al.^4^ A significant positive correlation (Figure 2C; generalized additive model: *R*^2^ = 0.55, *p* < 0.05; partial least squares: *R*^2^ = 0.19, *p* < 0.05) was observed between plastic abundance and microbial plastic degradation potential. The generalized additive model revealed a nonlinear tipping point at 7.44 particles/m^3^: below this threshold, the degradation potential increased sharply with increasing plastic load, whereas beyond this threshold the potential plateaued despite increasing pollution levels. This pattern suggests that excessive contamination, especially under unfavorable climatic regimes, may drive ecosystems toward functional instability and reduce microbial resilience.

Collectively, these findings demonstrate that plastic waste and climatic conditions interactively govern microbial degradation capacity in lake ecosystems. Recognizing this coupling provides critical insight for climate-adaptive management, emphasizing the need for targeted monitoring and stricter plastic control in regions where climatic variability limits microbial self-remediation potential while maintaining pollution below ecological tipping points to prevent irreversible functional disruptions.

### LPDMAG catalog

**Figure 3:**
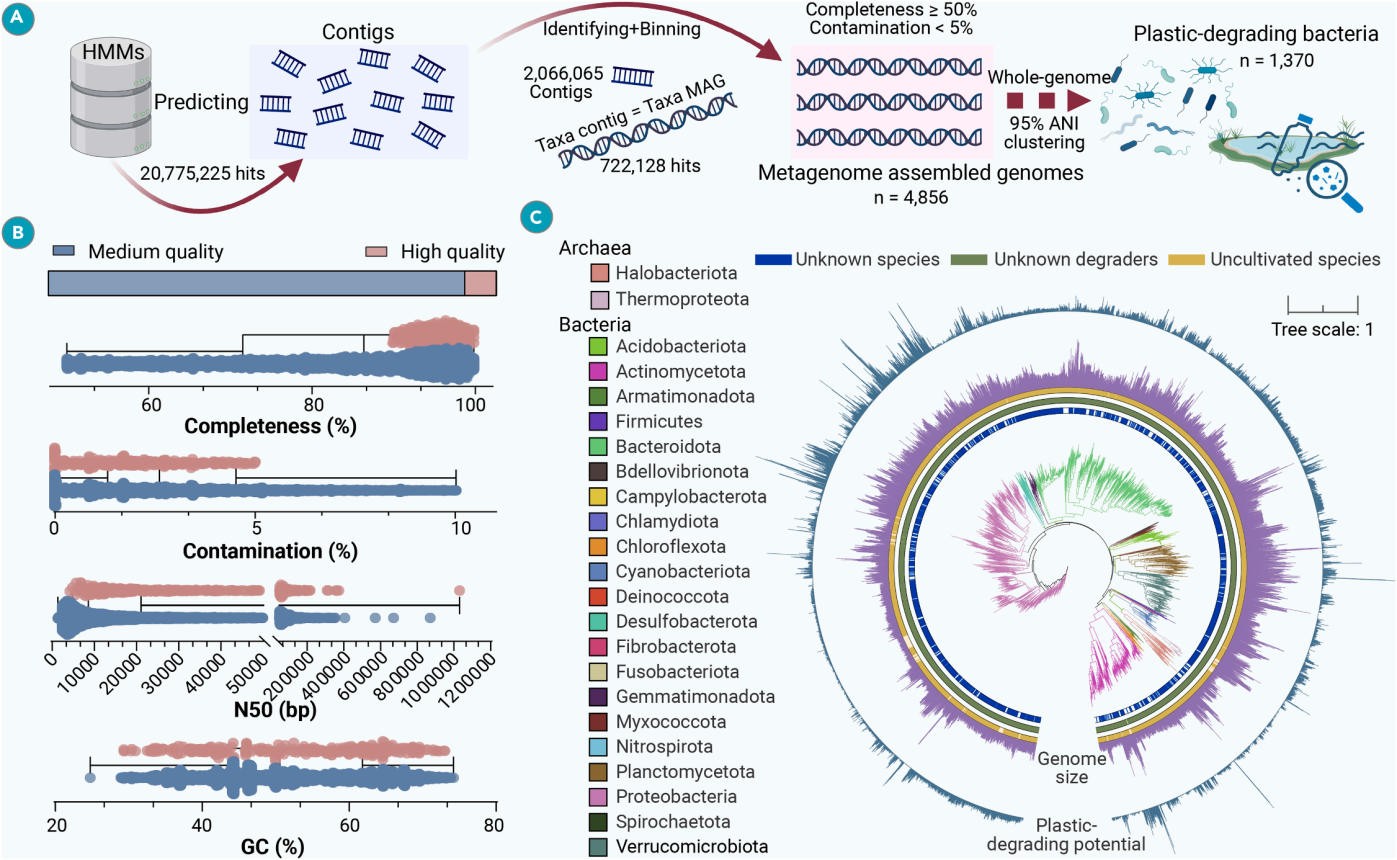
Identification of candidate plastic-degrading bacteria across global lakes (A) Overview of the computational framework developed to assign putative plastic-degrading enzyme-encoding genes to their microbial hosts based on MAGs. (B) Distribution of quality metrics across 4,856 MAGs; blue and red denote medium- and high-quality MAGs, respectively. (C) Phylogenetic tree of 4,856 candidate plastic-degrading MAGs recovered from lake ecosystems worldwide. HMMs, hidden Markov models; MAGs, metagenome-assembled genomes.

To reconstruct previously unexplored genomes of candidate plastic-degrading bacteria, we performed a large-scale single-sample metagenomic assembly on all samples and retained only those datasets in which the taxonomic affiliation (at the family level) of any hits found in candidate plastic-degrading contigs was consistent with the overall taxonomy of the MAGs (Figure 3A). After identification and filtration, we generated a total of 4,856 MAGs with plastic-degrading potential that met or exceeded the minimum information about a metagenome-assembled genome medium-quality standard (completion ≥50%, contamination ≤5%),^43^ with a mean completeness of 82.20% (±14.43%) and a mean contamination rate of 3.15% (±2.38%), which we refer to as the LPDMAG catalog. The N50 size of these MAGs ranged from 1,370 to 1,032,389 bp, and the GC content varied from 25% to 75% (Figure 3B; Table S4). Approximately 341 (7.02%) LPDMAGs were identified as high-quality genomes with completeness >90%, contamination <5%, the presence of 23S, 16S, and 5S rRNA genes and at least 18 tRNAs according to recent guidelines. Moreover, 1,744 (35.91%) of the LPDMAGs had completeness ≥90% and contamination ≤5% but lacked a complete set of rRNA genes or had fewer than 18 tRNAs, further suggesting that recovering a full complement of rRNA gene remains challenging for nearly complete MAGs.^43^ In addition, LPDMAGs covered 83% of the collected samples spanning 86 lakes, indicating that this catalog is a substantial but incomplete fraction of the diversity of candidate plastic-degrading bacteria from lakes worldwide.

### MAG analyses identify 1,370 species-level candidate plastic-degrading bacteria in lakes

To explore the taxonomic components in the LPDMAG catalog, we clustered the 4,856 MAGs into 1,370 species-level genome bins (denoted as candidate plastic-degrading bacteria) on the basis of a 95% whole-genome average nucleotide identity threshold (Figure S7). These candidate degraders were taxonomically annotated into 21 bacterial phyla and 2 archaeal phyla on the basis of the Genome Taxonomy Database, which is widely used.^44^ Next, we constructed a phylogenetic tree of all the LPDMAGs (Figure 3C), indicating that the diversity of candidate plastic-degrading bacteria across the Tree of Life was expanded by this catalog. By comparing 400,596 reference genomes, including 127,100 genomes from the RefSeq database (November 2022) and 273,496 genomes from GenBank (November 2022), we identified 609 unknown and 761 known species at the threshold of 95% average nucleotide identity. The proportion of singleton MAGs among unknown MAGs (73.9%) was substantially greater than that among known plastic-degrading bacteria (49.0%), indicating the critical contribution of the LPDMAG catalog to recovering rare species of lake microbiomes. Moreover, we compared these species against nearly 1,008 previously reported species-level plastic-degrading bacteria from PlasticDB (https://plasticdb.org/) and other literature sources (Table S5). In total, we identified 99.9% species-level, 99.3% genus-level, 79.9% family-level, 77.2% order-level, 78.1% class-level, and 69.6% phylum-level novel candidate plastic-degrading bacteria within the LPDMAG catalog (Table S6), substantially expanding the molecular resources of candidate plastic-degrading bacteria.

Most candidate degraders exhibited activity toward multiple plastic types, indicating substantial functional overlap rather than strict substrate separation (Figure S8). For instance, in the PLA-contaminated sites, 93% of the identified candidate degraders encoded enzymes targeting not only PLA but also other polymers, and their relative abundance was significantly higher than that observed in uncontaminated sites (Figure S9). These patterns suggest that degradation preferences are primarily governed by the enzyme repertoire rather than by taxonomic boundaries. Nonetheless, a few lineages showed narrow specialization, such as *Dermatophilaceae*, which appeared to be restricted to polyhydroxyalkanoates, and *Streptococcaceae*, which is restricted to polyurethane. Overall, these results reveal that functional convergence and enzymatic versatility are widespread among candidate plastic-degrading microbes, with specialization confined to a limited subset of taxa.

Across all the metagenomes, an average of 567 candidate plastic-degrading taxa per sample was identified, with a mean abundance of 813 reads per kilobase per million mapped reads (Figure S10). Although degrader diversity remained relatively consistent among the lakes, their abundance exhibited pronounced spatial heterogeneity, with several sites showing marked enrichment. These patterns suggest that local environmental conditions and contamination pressure jointly shape the biogeography of candidate plastic-degrading microorganisms in freshwater ecosystems. To further examine ecological interactions, we constructed a co-occurrence network based on abundance correlations between LDPMAGs and non-degrading taxa (Figure S11). The network comprised four major modules (modules 2, 3, 4, and 30) dominated by non-degraders such as *Comamonas*, *Magnetospirillum*, *Polynucleobacter*, and *Granulicella*, which were strongly associated with candidate plastic degraders. These associations indicate potential metabolic complementarity and ecological cooperation, whereby non-degraders may contribute to nutrient cycling or biofilm stabilization, supporting the persistence and activity of candidate degraders under varying lake conditions.

### Hot zones of candidate plastic-degrading bacteria in lakes

To identify the ecological hot zones of candidate plastic-degrading bacteria, we analyzed metagenomic data from a plateau karst lake encompassing lake water, plastisphere, and sediment samples with quantified plastic debris concentrations.^10^ The plastic debris concentration ranged from 20.27 to 58.80 n L^−1^ in the lake water and 583.33 to 996.67 n kg^−1^ in the sediments, indicating measurable plastic pollution across all sampling sites. The plastisphere harbored the highest microbial richness and diversity, significantly exceeding those of the lake water and sediments (Figure S12). Moreover, lakes with relatively high plastic pollution levels presented correspondingly high abundances of candidate plastic-degrading bacteria in sediments, suggesting that contamination intensity promotes the enrichment of functional degraders. These findings reveal that both the plastisphere and lake sediments act as key ecological reservoirs (hot zones) for candidate plastic-degrading microorganisms. The plastisphere provides a selective and nutrient-rich interface that facilitates microbial colonization and metabolic cooperation, whereas sediments serve as long-term sinks where plastic-associated degraders accumulate and persist.

### Promising candidate plastic-degrading bacteria for environmental application

**Figure 4:**
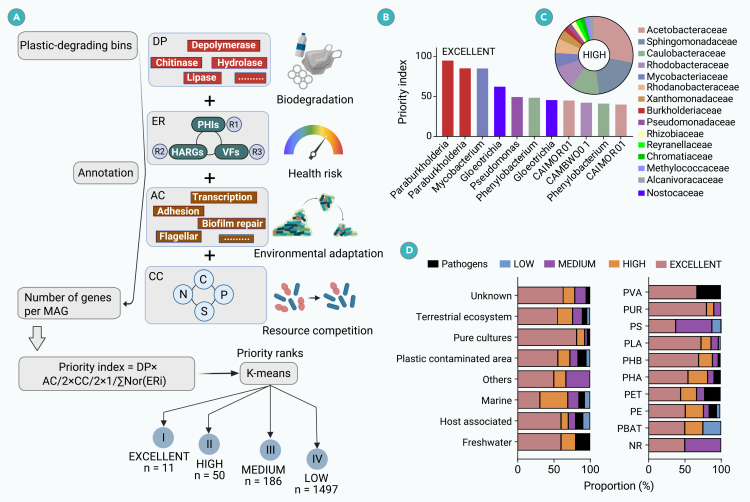
Priority index of candidate plastic-degrading bacteria (A) Framework for calculating the priority index of 1,744 candidate plastic-degrading MAGs with near-complete genomes, based on four factors: plastic-degrading potential (DP), ecological risk (ER), adaptation capacity (AC), and competition capacity (CC) (see materials and methods for details). (B) Priority index and taxonomic composition of candidates classified as EXCELLENT degraders. (C) Taxonomic proportions of candidates classified as HIGH degraders. (D) Priority index of experimentally validated plastic-degrading strains. MAGs, metagenome-assembled genomes; PHI, pathogen-host interaction (hypervirulence); HARGs, high-risk antibiotic resistance genes; VFs, virulence factors; C, carbon; N, nitrogen; P, phosphorus; S, sulfur.

To better understand the functional characteristics of these candidate plastic-degrading bacteria, we predicted full-length putative protein sequences from the 1,744 high-completeness MAGs (>90% completeness, <5% contamination) of the LDPMAG catalog. We developed a novel computational framework to assess the environmental applicability of these candidate degraders in the environment, compiling their four functional characteristics: plastic-degrading potential, ecological risk, adaptation capacity, and competitive capacity (Figure 4A). In addition to their plastic-degrading potential, the key step is that candidate degraders can survive on plastic surfaces under nutrient limitation and exogenous stress.^22^ Biofilm formation potential represents the capacity of candidate plastic-degrading bacteria to adapt to a new environment. Next, the potential for recycling core nutrient elements (carbon, nitrogen, phosphorus, and sulfur) was used to assess the competitive capacity of candidate degraders in resource-poor environments. In addition, we assessed the ecological health risk of these candidate degraders on the basis of commonly used risk factors,^42^^,^^45^ antibiotic resistance genes (with high human health risk), pathogenic factors (hypervirulence-related genes), and virulence factors. Overall, we calculated the priority index of the candidate plastic-degrading bacteria (for details, see materials and methods). Using the K-means algorithm, a well-known and widely used clustering method,^46^ we divided the candidate degraders into EXCELLENT (*n* = 11), HIGH (*n* = 50), MEDIUM (*n* = 186), and LOW (*n* = 1,068) categories. The most important EXCELLENT candidate degrader, *Paraburkholderia*, showed strong environmental application potential, with the highest degradation potential, adaptation, and competition capacity, and the lowest risk (Figure 4B). *Acetobacteraceae*, *Sphingomonadaceae*, and *Caulobacteraceae* were the main HIGH-priority candidate plastic-degrading bacteria (Figure 4C). In addition, the priority score of candidate plastic-degrading bacteria varied among different plastic types; for example, *Acetobacteraceae* was a higher priority when applied to polyethylene glycol, polyurethane, and phthalic acid pollution than when applied to other types of pollution (Figure S13). Therefore, in practical applications of this framework, the type of polluting plastic should be explicitly identified and considered.

To illustrate the applicability and necessity of this priority assessment framework, we calculated the priority indices of 165 experimentally validated plastic-degrading strains (with available species-level reference genomes) (Table S7). These strains were mainly isolated from pure cultures, terrestrial water, and plastic-contaminated water, whereas a few strains were isolated from freshwater. As shown in Figure 4D, most plastic-degrading strains fall into the EXCELLENT and HIGH categories, although a small fraction is clustered in the LOW and pathogen categories. Importantly, degraders of polyester-based plastics such as PLA, PET, and polybutylene adipate terephthalate were classified into the EXCELLENT and HIGH categories, reflecting their relatively well-characterized enzymatic pathways and higher biodegradation potential. In contrast, degraders of more recalcitrant plastics, such as polyethylene, and polystyrene, were more often assigned to the LOW or MEDIUM categories, indicating limited efficiency under current conditions.

### Enrichment strategies for high-priority candidate plastic-degrading bacteria

**Figure 5:**
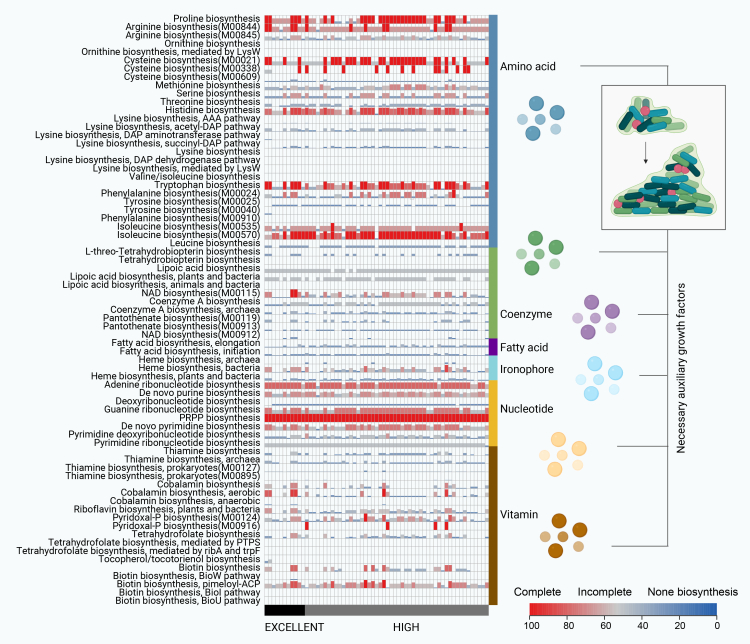
Biosynthetic profiles of essential growth factors in high-priority candidate plastic-degrading bacteria Heatmap showing the completeness of biosynthetic pathways for essential growth factors across EXCELLENT and HIGH-priority candidate plastic-degrading bacteria. Pathways are grouped into major functional categories, including amino acids, coenzymes, fatty acids, ironophores, nucleotides, and vitamins. Each row represents a specific biosynthetic pathway, and each column represents an individual candidate degrader. Color intensity indicates pathway completeness, with red denoting complete pathways, light colors indicating partial pathways, and blue indicating the absence of biosynthesis. The schematic on the right illustrates the conceptual framework linking growth factor auxotrophy to microbial community assembly and enrichment under nutrient-limited conditions, highlighting the role of necessary auxiliary growth factors in supporting the colonization and persistence of plastic-degrading bacteria.

To guide future enrichment, isolation, and culture, we manually restructured a total of 73 biosynthetic pathways with 299 proteins and 469 KO numbers, covering 41 essential growth factors (e.g., vitamins, amino acids, nucleotides, and coenzymes) for bacteria or archaea, on the basis of the Kyoto Encyclopedia of Genes and Genomes database (Table S8). As expected, many EXCELLENT and HIGH candidate plastic-degrading bacteria lacked complete biosynthetic pathways for some of these essential nutrients (Figure 5). The biosynthesis profiles of essential growth factors corresponding to each candidate degrader were not unique; thus, we formulated eight template media according to the metabolic profile of high-priority degrading bacteria to enrich and isolate more previously unknown degrading bacteria (Table S9).

**Figure 6:**
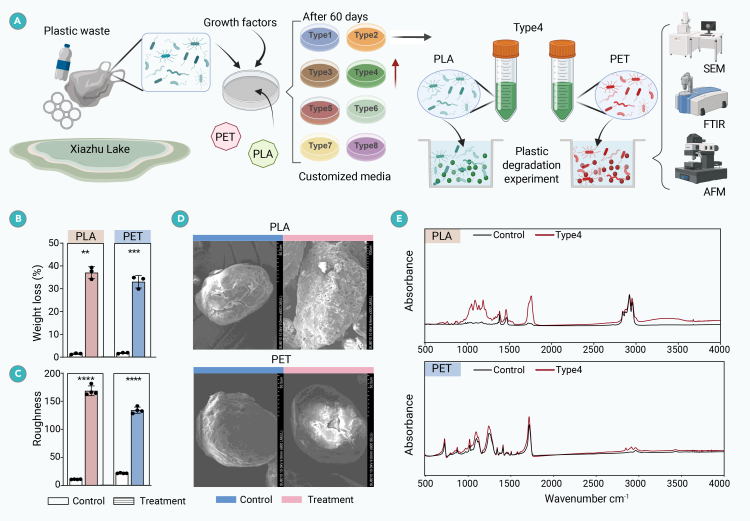
Enrichment and experimental validation of potential plastic-degrading bacterial communities (A) Microbial communities associated with plastic waste from Xiazhu Lake were enriched in customized media containing PLA or PET as the sole carbon source. (B) Weight loss of PLA and PET plastics after incubation with enriched microbial communities, showing significantly higher degradation in treatment groups compared with controls. Data are presented as mean ± SEM. (C) Surface morphology of PLA and PET after microbial treatment, revealing clear signs of microbial degradation. Scale bars, 50 μm. (D) Quantification of surface roughness of PLA and PET plastics following microbial treatment. (E) Fourier transform infrared spectra of PLA and PET plastics before treatment (black curves, control) and after incubation with the type 4 enriched bacterial community (red curves, treatment).

Genomic analysis revealed that the EXCELLENT candidate plastic-degrading bacteria exhibit high degradation potential for both PLA and PET, representative biodegradable and non-biodegradable plastics, respectively, which dominate global plastic production volumes.^47^ We therefore collected PLA and PET fragments after 6 months of *in situ* enrichment in Xiazhu Lake (30.5156°E, 120.0483°N) to obtain ecologically primed plastisphere samples. These were subsequently inoculated into eight chemically defined media containing PLA or PET as the sole carbon source (Figure 6A). After 60 days, the type 4 cultured bacterial community, which was most significantly enriched in both PLA and PET groups, was used to verify the degradation ability of plastics. Changes in surface morphology and weight loss serve as key indicators of plastic degradation. Notably, significant weight reductions were observed in PLA and PET groups after inoculating the type 4 cultured bacterial community for 90 days, which presented weight losses of 37.1% and 33.1%, respectively (Figure 6B). Scanning electron microscopy analysis revealed the development of distinct surface features, such as pits, gullies, folds, and grooves, along with localized detachment on the surfaces of PLA and PET following bacterial treatment (Figure 6C). Meanwhile, atomic force microscopy revealed substantial increases in surface roughness in the treatment groups, with PLA and PET showing 15.6- and 6.2-fold increases, respectively, compared with those of the control group (Figures 6D and S14). Furthermore, the FTIR spectra of these plastics clearly revealed that the chemical bonds within the PLA and PET were broken down (Figure 6E). ple, FTIR analysis of PLA showed the shifts and intensity changes in the C=O stretch (1,735–1,750 cm^−1^) and C-O stretch (1,000–1,300 cm^−1^), indicating the hydrolysis of PLA.

### Excellent plastic-degrading strain *S. ficaria* HfyG-1 isolated from lakes

**Figure 7:**
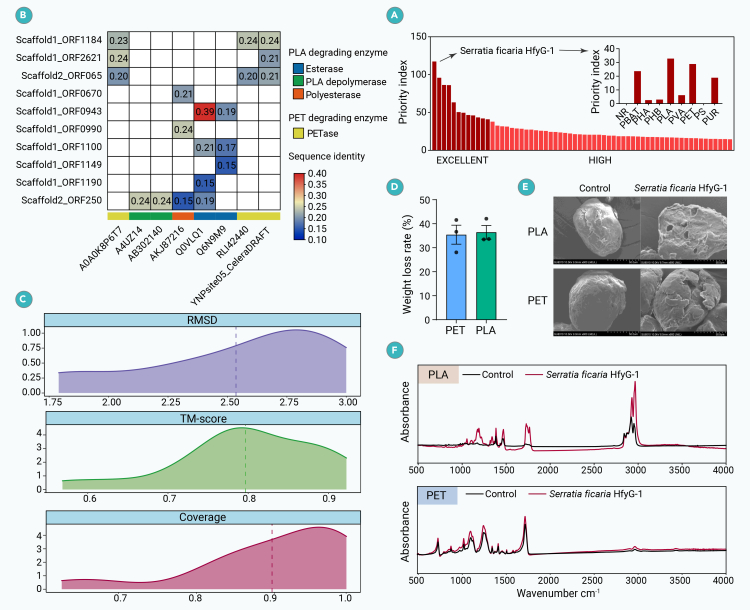
Genomic and experimental characterization of the plastic-degrading bacterium *S. ficaria* HfyG-1 (A) Identification of *S. ficaria* HfyG-1 as an EXCELLENT candidate plastic degrader, with strong predicted potential for polylactic acid (PLA) and polyethylene terephthalate (PET) degradation. (B) Sequence identity of 10 putative plastic-degrading enzymes encoded by *S. ficaria* HfyG-1 compared with reported reference enzymes. (C) Structural comparison between predicted enzymes and known plastic-degrading enzymes based on root mean-square deviation, TM score, and structural coverage. (D) Weight loss rates of PLA and PET after 90 days of incubation with *S. ficaria* HfyG-1. (E) Surface morphology of PLA and PET plastics after treatment with *S. ficaria* HfyG-1, showing clear evidence of microbial degradation. Scale bars, 50 μm. (F) Fourier transform infrared spectra of PLA and PET plastics before treatment (black curves, control) and after inoculation with *S. ficaria* HfyG-1 (red curves, treatment). TM, template modeling.

We obtained 22 and 31 independent bacterial colonies from type 4 communities incubated with PLA and PET particles, respectively, under conditions where plastics served as the sole carbon source (Figure S15). The dominant colonies with the most robust growth and degradation activity were selected for whole-genome sequencing, revealing a shared *S. ficaria* strain (HfyG-1). By evaluating four priority indices—plastic-degrading potential, ecological risk, adaptation capacity, and competition capacity—we identified *S. ficaria* HfyG-1 as an EXCELLENT plastic-degrading strain, particularly noted for its potential to degrade both PLA and PET (Figure 7A). Moreover, genomic interrogation identified 10 putative plastic-degrading enzymes, including esterases, PETases, and specialized PLA depolymerases, which shared generally low sequence identities with reported plastic-degrading enzymes, with an average identity of <30% (15%–39%). However, structural comparisons showed high overlap, with mean template modeling scores >0.8, coverage >0.9, and root mean-square deviation <3.0, indicating that the putative enzymes were structurally conserved but sequence divergent, strongly supporting their novelty (Figure 7B). Furthermore, metatranscriptomic surveys from six lakes across five countries showed that these putative enzymes were frequently detected (Figure S16), suggesting that they are actively expressed in natural environments and likely to play functional roles in plastic degradation *in situ*.

To investigate potential synergistic effects among microbial communities during plastic degradation, we analyzed the full-length 16S rRNA datasets from the PLA and PET degradation microcosms at 30 and 60 days. The results showed that *S*. *ficaria* was significantly enriched overtime on both PLA and PET surfaces (*p* < 0.05; Figure S17A). Co-occurrence network analysis further revealed that *S. ficaria* extensively interacted with other bacterial taxa, such as *Glutamicibacter*, *Achromobacter*, *Ralstonia*, and *Lactobacillus*, forming stable consortia that may facilitate substrate breakdown and nutrient recycling (Figures S17B and S17C). These findings demonstrate that plastic degradation in the microcosm is likely driven by synergistic microbial networks rather than by single degraders acting independently.

To further validate the degradation capacity of *S. ficaria* HfyG-1, we inoculated this strain into type 4 medium with PLA and PET as the sole carbon source, and the weight loss rate was up to 35.4% and 36.4%, respectively, after 90 days (Figure 7D). Scanning electron microscopy also revealed that the surfaces of PLA and PET contained obvious pits, grooves, and folds after treatment with degrading strains (Figure 7E). FTIR analysis revealed that the chemical groups of PLA and PET were damaged to varying degrees after being inoculated with plastic-degrading strains for 90 days (Figure 7F). Collectively, these results demonstrate that the isolated strain *S. ficaria* HfyG-1 exhibits robust viability and high environmental application potential, with demonstrated capability to degrade both PLA and PET. This evidence validates the effectiveness of our framework for identifying plastic-degrading bacteria.

## Discussion

Given its generally persistent nature, plastic pollution is possibly a hallmark of the Anthropocene.^22^ Lakes, as sentinels of environmental change,^48^ are suffering from heavy plastic pollution, and microbes have evolved strategies to cope with this contamination. This study describes the microbial potential for degrading plastics, including the majority of massively produced and globally polluting polymers, and predicts their geographical distribution across 182,661 lakes worldwide via machine learning. We demonstrated that plastic content is strongly associated with the plastic-degrading potential of microbes in lakes globally, suggesting that plastic pollution drives the accumulation of plastic-degrading potential. Therefore, mining ecological resources for plastic degradation helps to further understand the current situation of plastic pollution in lakes and is conducive to the subsequent development of plastic biodegradation technology.^49^

To the best of our knowledge, this is the first report of a MAG catalog with plastic-degrading potential for lake ecosystems, comprising 4,856 genomes of 1,370 species from 715 metagenomes. This catalog represents a step change compared with currently available studies on candidate plastic-degrading bacteria identified via metagenomes, which are limited by short sequence fragments, taxonomic annotation bias, small sample sizes, and uneven sequencing depths.^17^ Lakes host highly diverse and novel microbial populations, with 609 species-level genome bins representing new species, and approximately 99% of this catalog includes novel candidate plastic-degrading bacteria. The candidate plastic-degrading bacterial catalog provided in this study not only greatly expands the resources of plastic degradation but also complements microbial resources, such as the genomes from the LakePulse catalog,^50^ which is limited by the global coverage of lake ecosystems.

To understand the actual applicability of candidate plastic-degrading bacteria to the environment, it is necessary to consider their plastic-degrading potential, ecological risk, adaptation capacity, and likely competitiveness in the environment. Excellent candidate plastic-degrading bacteria should not only adapt to living on plastic surfaces and be able to compete for nutrients but also have high plastic degradation potential and pose little risk to humans, animals, plants, or the environment. Therefore, we developed a novel computational framework to assess the priority for potential environmental applications in the environment on the basis of these 4 factors. Using this framework, we classified 165 experimentally verified plastic-degrading strains into 4 priority ranks and found that most of these degraders were indeed high priority; however, some of these strains were identified as LOW priority or opportunistic pathogens, highlighting the usefulness of this framework for minimizing the ecological risk of any future application of plastic-degrading strains.

Importantly, our study demonstrated that only 3.7% of these plastic-degrading bacteria were readily cultivable by comparing 400,596 reference genomes from the RefSeq database and GenBank; thus, enriching, isolating, and culturing such strains is the greatest obstacle to their application. To address this dilemma, based on the genomic data of LPDMAGs and their priority index analysis, we provide an enrichment and isolation strategy for EXCELLENT candidate plastic-degrading bacteria. Several studies have indicated that the functional annotation of MAGs is critical for metabolic pathway reconstruction and can be used to help with their cultivation.^51^^,^^52^^,^^53^^,^^54^ For example, Renesto et al.^55^ revealed the metabolic deficiencies of *Tropheryma whipplei* MAGs in amino acid biosynthesis and isolated them by modifying the growth medium via the addition of nine necessary amino acids. Our study reconstructed the essential nutrient biosynthesis pathway of candidate plastic-degrading bacteria and demonstrated that most high-priority degraders were auxotrophs. This may be because auxotrophy is an adaptive trait, and it might be particularly advantageous when essential metabolites can be competitively obtained from the 1surrounding environment or from nearby cells.^55^ There is some evidence for auxotrophy-mediated nutrient exchange in microbial communities with hydrocarbon degradation potential,^42^ which further supports our findings.

Guided by the metabolic profiles of high-priority candidate degraders, we formulated eight template media and successfully enriched PET- and PLA-degrading bacteria from Xiazhu Lake. Reported degradation efficiencies vary widely among strains and conditions—for example, *Ideonella sakaiensis* 201-F6 almost completely degraded low-crystallinity PET films in 42 days,^12^ *Bacillus* strains achieved 30%–40% degradation within a month,^56^ and *Amycolatopsis* HT-32 degraded 60% PLA in 2 weeks, whereas natural soil degradation was extremely slow (0.23% in 12 months).^57^ In our assays, the mixed consortium degraded 33.1% PET and 37.1% PLA within 90 days, and the pure culture of *S. ficaria* HfyG-1 achieved 36.4% and 35.4% degradation, respectively. Although slightly less efficient than optimized laboratory strains, HfyG-1 is distinctive in degrading both PET and PLA under identical conditions and was identified as an excellent, environmentally resilient degrader suitable for safe and sustainable plastic management.

Moreover, although this study integrates large-scale metagenomic analyses with experimental validation, several limitations should be acknowledged. The identification of plastic-degrading microbes and enzymes is primarily based on genome-resolved predictions and computational screening, and therefore reflects putative degradation potential rather than definitive enzymatic activity. Future studies incorporating comprehensive biochemical characterization of individual enzymes and systematic validation of candidate strains will be essential to fully resolve their functional roles. Meanwhile, the current prioritization framework was developed mainly for millimeter- to centimeter-scale plastic fragments, which provide sufficient surface area for microbial colonization and measurable degradation. Smaller microplastics (<300 μm) and nanoplastics exhibit distinct physicochemical properties, including altered surface area-to-volume ratios, diffusion constraints, and enzymatic accessibility, and were beyond the scope of this study. Despite these limitations, our work provides the first global, genome-resolved catalog of candidate plastic-degrading microbes in lake ecosystems and establishes a data-driven framework that links degradation potential with ecological viability, offering a systematic and transferable strategy for future exploration and application of plastic-degrading microbial resources.

In conclusion, this study presents a global catalog of candidate plastic-degrading microbes and enzymes across lake ecosystems through large-scale metagenomic analysis. We developed a computational framework to categorize candidate degraders based on their genomic and ecological traits and reconstructed their essential nutrient biosynthesis pathways, facilitating targeted enrichment and cultivation from the plastisphere. By integrating genome-resolved analyses with experimental validation, we revealed that plastic degradation is likely driven by synergistic microbial networks rather than single degraders, both in natural communities and in microcosms. By designing customized media, we successfully isolated *S. ficaria* HfyG-1, which is capable of degrading PLA and PET. Together, these findings establish a proof-of-concept framework for identifying and harnessing plastic-degrading microbial consortia, offering a new perspective for microbial resource exploration in natural environments.

## Resource availability

### Materials availability

This study did not generate new unique materials.

### Data and code availability

• Raw metagenomics data are publicly available in the ENA (https://ebi.ac.uk/ena/) and NCBI (https://ncbi.nlm.nih.gov/sra/) databases.
• The raw metatranscriptomic datasets used in this study were retrieved from public repositories under accession numbers SRA: PRJNA691101, PRJNA1089960, PRJNA1161448, and ENA: PRJEB61515.
• Global spatial covariates, the links of which are presented in Table S10, are publicly available.
• The whole-genome sequencing data of an isolated plastic-degrading strain are available through the NCBI Sequence Read Archive under BioProject ID SRA: PRJNA1144214.
• All the data needed to evaluate the conclusions in the paper are presented in the paper and/or the supplemental information.
• All the scripts and codes for gene annotation, abundance calculation, machine learning, statistical analysis, and visualization used in this study are available online at https://github.com/QiZhang11/Lake-plastic-degraders.

## Funding and acknowledgments

This work was supported by the 10.13039/501100001809National Natural Science Foundation of China (22241603-3, 42307158, and 42507185), the Quzhou Joint Fund of the Zhejiang Provincial Natural Science Foundation of China (LQZSZ26B070002), the Project of the Scientific and Technological Program of Shaoxing (2024A13013), and the Ningbo Public Welfare Key Science and Technology Plan Project (2023S011). The funders had no role in study design, data collection and analysis, decision to publish, or preparation of the manuscript.

## Author contributions

Q.Z. and Zhenyan Zhang designed the study with guidance from H.F.Q. and Y.-G.Z. Q.Z. wrote the first draft of the manuscript and Y.-G.Z., H.F.Q., M.C.R., L.C., and D.Z. contributed substantially to revisions. Q.Z., T.Z.W., G.Y.Q., M.K.J., and B.F.C. contributed to the all functional annotation of metagenome-assemble genomes. Q.Z., T.Z.W., M.X.W., and Zhenyan Zhang performed all the metagenomic analysis. Q.Z., Zhenyan Zhang, and T.L. were responsible for the machine learning model construction and related data analysis. Q.Z. and Y.T.Y. perform all the microbial metabolic profiles reconstruction based on the genome analysis. Q.Z., G.Y.Q., and Ziyao Zhang experimented target enrichment plastic degraders. Q.Z. performed the visualization of all data and the artistic design of all figures. H.F.Q., Q.Z., Zhenyan Zhang, and D.Z. acquired funding for this project. All authors contributed to the manuscript and approved the final version.

## Declaration of interests

The authors declare no competing interests.

## Supplemental information

Data S1. Tables S1–S11

Document S2. Article plus supplemental information
