# Supplementary material for: Potential plastic biodegradation in lakes worldwide
Source: Innovation (Camb). 2026 Mar 3;7(8):101338. doi: 10.1016/j.xinn.2026.101338 (PMC13457188; doi:10.1016/j.xinn.2026.101338)
Supplement: Supplementary file 1 [file mmc1.pdf]

The Innovation, Volume *7*

Supplemental Information

Potential plastic biodegradation in lakes worldwide

Qi Zhang, Zhenyan Zhang, Ziyao Zhang, Guoyan Qin, Mingkang Jin, Bingfeng Chen, Yitian Yu, Tingzhang Wang, Meixia Wang, Tao Lu, Dong Zhu, Li Cui, Haifeng Qian, Matthias C. Rillig, and Yong-Guan Zhu

Supplemental Information

Supplemental methods

Datasets

We searched the European Nucleotide Archive (ENA; https://ebi.ac.uk/ena/) and NCBI Sequence Read Archive (https://ncbi.nlm.nih.gov/sra/) for metadata from 01-16-2023, using the terms "Lake," "Metagenome," and "Freshwater." We used the following metagenome selection criteria to minimize possible bias: (i) pond samples, such as artificial or natural ponds, were excluded; (ii) samples were not collected from aquatic organisms and sediments or lakes adjacent to pollution sources, extreme climates, treatments, and inaccurate coordinate records; (iii) only paired-end sequencing reads in FASTQ format were included, and the average read length of the metagenomes exceeded 100 base pairs. We collected 715 metagenomic samples from 95 lakes located across five continents and 19 countries.

Metagenomic assemblies and reconstruction of LPDMAGs

Metagenomic assemblies were reconstructed via MEGAHIT v1.2.9 with metawrap assembly and metawrap binning commands.[1] The reads were mapped to contigs via Bowtie 2 (v.2.3.5.1) with default parameters,[2] and the resulting alignment files were sorted and indexed via SAMtools (v1.9).[3] The sorted BAM file was used to bind the contigs via a combination of CONCOCT [4] (v.1.0.0), MaxBin 2.0 (v.2.2.6),[5] and metaBAT2 (v.2.12.1) with default parameters to identify the MAGs.[6] During refinement, the completeness and contamination of all MAGs were estimated via the lineage-specific workflow via CheckM (v1.0.11) to generate MAGs with $\geq$ 50% completeness and < 10% contamination.[7] This quality filtering step was applied to minimize the influence of fragmented assemblies and chimeric bins, which can introduce noise into functional prediction when short or misassembled sequences are considered. Hidden Markov model (HMM) searches were then performed across all retained MAGs. To further reduce false-positive functional assignments associated with short contigs and assembly artifacts, we restricted candidate hits to contigs longer than 10 kb. In addition, we required that the taxonomic affiliation of each contig harboring a candidate gene was consistent with the overall taxonomy of its

corresponding MAG, thereby ensuring taxonomic coherence between predicted functions and genome-level context. After filtering, we produced a total of 4,856 LPDMAGs and clustered them into 1,370 species-level genome bins on the basis of 95% ANI via dRep (v2.2.4).[8] The current methods for analyzing putative plastic-degrading enzymes result in similar problems of broadening the scope for identifying candidate plastic-degrading bacteria. To calculate the community assembly of these candidate degrading bacteria in the lake, we used CoverM (v0.7.0) to successfully identify a total of 765 candidate plastic-degrading taxa across all metagenomes.[9] Ribosomal and transfer RNAs were identified via the nhmmer function (part of HMMER 3) from Barrnap (v.0.9) and tRNAscan-SE (v.2.0.9) with default parameters, respectively.[10] The high-quality MAGs were defined as those with > 90% completeness, ≤ 5% contamination, ≥ 18/20 tRNA genes, and 5S, 16S, and 23S rRNA genes, and the remaining MAGs were classified as medium-quality MAGs.

Enzyme dataset and construction of HMMs

On the basis of published studies and databases,[11,12] we collected a dataset of 184 sequenced plastic-degrading enzyme-encoding genes spanning 10 plastic types (Dataset S11). Before model construction, each enzyme sequence was manually curated and verified by cross-checking its origin, substrate type, and supporting literature. Controversial or retracted entries were excluded to ensure reliability. All reference enzymes were further categorized into three functional groups according to polymer type: polyolefin-degrading enzymes (PE and PS); conventional polyester-degrading enzymes (PET and PU); and biodegradable plastic–degrading enzymes (PLA, PBAT, PHAs, PHB and PVA). To expand the sequence space for building the HMMs, we downloaded the UniProt TrEMBL database (on May 16, 2023) with more than 2.4 billion candidate protein sequences, which was used to obtain the representative sequences via BLAST (v2.6) with default settings, except for an E-value cutoff of 1e-10 and an identity cut off 90%.[13] We next extracted each sequence ID and removed redundant representative sequences via CD-HIT with default settings,[14,15] with the exception of a word size of 5, a cluster size of 5, and a sequence identity cutoff of 60%. Finally, we performed multisequence alignment of these

representative sequences via MAFFT and constructed a total of 413 HMMs (including all representative sequences) via HMMER v3.3 hmmbuild utility (http://hmmer.org/) with default settings.[16, 17]

We detected the genes from the predicted proteins of the metagenomes on the basis of the constructed HMM profiles via hmmsearch from HMMER v3.3, with an E value cut off below 1e-10. Only the lowest E value and bit score hits were retained for each gene in the metagenomes. To minimize potential false positives and enhance annotation accuracy, we first used the customized HMM models to scan all metagenomic contigs and identify candidate open reading frames (ORFs) that matched plastic-degrading enzyme profiles. The ORFs aligned to these HMM models were then further analyzed using the machine learning-based CLEAN model, which employs official open-source pretrained weights to predict Enzyme Commission (EC) numbers.[18] By comparing the predicted EC numbers with those of known plastic-degrading enzymes (Dataset S2), we validated functional consistency and refined the annotation. The combined HMM–machine learning pipeline ultimately identified 45,154 nonredundant enzyme hits across all metagenomes, covering ten major plastic types (biodegradation pathways, see Figure S18).

Annotation of functions in LPDMAGs and reference genomes

Antibiotic resistance genes in LPDMAGs (1,744 high-completeness MAGs) and bacterial genomes were annotated with CARD (v2020) using their recommended tool, RGI (v5.1.1),[19] with the default parameters for genomes. Pathogenic (hypervirulence) and virulence factors were annotated with the Pathogen-Host Interactions (www.phi-base.org/) and Virulence Factors of Bacterial Pathogens (mgc.ac.cn) databases using DIAMOND (v2.1.4; e-value ≤ 10^{-3}, identity: 70%, coverage: 60%).[20] Moreover, we annotated the potential for the biosynthesis and repair of biological membrane from LPDMAGs and reference genomes with a constructed database based on the UniprotKB database (http://www.uniprot.org/) with biological membrane-related genes. Furthermore, we manually compiled a comprehensive database of genes involved in carbon, nitrogen, phosphorus, and sulfur (CNPS) cycles from NCycDB,[21] SCycDB,[22] QMEC,[23] and GeoChip 5.0.[24] The potential ecological functions of the

LPDMAGs were annotated via the CNPS database created by BWA (v0.7.13).[3]

Reconstruction of the biosynthesis pathways of growth factors in nearly complete LPDMAGs

We manually reconstructed a total of 73 biosynthetic pathways with 299 proteins and 469 KO numbers, covering 41 essential growth factors (e.g., vitamins, amino acids, nucleotides, and coenzymes) for bacteria or archaea (Dataset S8). To ensure the integrity of the biosynthesis of each growth factor, we considered all pathways, such as cysteine biosynthesis, which was associated with three genes (M00021, M00338 and M00609), and biotin biosynthesis, which included the BioU, I, F and W pathways. Moreover, we also considered the biosynthesis of the intermediates required in the biosynthesis pathway of each growth factor, such as pimeloyl-ACP for biotin biosynthesis. We annotated 1,744 high-completeness MAGs with these biosynthetic pathways on the basis of the Kyoto Encyclopedia of Genes and Genomes database via DIAMOND (v2.1.4; e value ≤ 10^{-3}, identity: 70%, coverage: 60%).[20]

Calculation of a priority index of candidate plastic-degrading bacteria

We developed a novel computational framework to assess the priority of strains for potential application in the environment on the basis of four factors: plastic-degrading potential, ecological risk, adaptation capacity, and competition capacity. The number of HMM hits in each LPDMAG indicates the plastic-degrading potential. Biofilms, the predominant mode of microbial life, are considered successful evolutionary adaptation for bacteria. By growing in biofilms, plastic-degrading bacteria can withstand external stress (such as oxidative stress, nutrient deficiencies, etc.), improving their chances of survival and reproduction.[25] Once the degraders are adapted to the plastic surface environment, nutritional restriction becomes the biggest obstacle, and better potential degraders can effectively utilize nutrient elements.

First, we calculated the adaptation capacity of each candidate plastic-degrading bacteria as follows:

$$F_{(AC)} = \sum_{i=1}^{n} Nor(F_{(AC)}i) \quad (1)$$

where $F_{(AC)}$ is the adaptation capacity of candidate plastic-degrading MAGs.

We thus took the potential of participating in carbon, nitrogen, phosphorus, and sulfur cycles as an indicator of the competitive capacity of candidate plastic-degrading bacteria.

Next, we calculated the competitive capacity of each candidate plastic-degrading bacteria as follows:

$$F_{(CC)} = \sum_{i=1}^{n} Nor(F_{(CC)}i) \quad (2)$$

where $F_{(CC)}$ is the competitive capacity of candidate plastic-degrading MAGs.

In addition, pathogenic (hypervirulence) and virulence factors underpin the pathogenicity and toxicity of these candidate degraders to animals, plants, and humans,[26] and antibiotic resistance genes with high risk also indicate a health risk for humans,[27] all of which represented the ecological risk of candidate degraders.
We calculated the ecological risk of each candidate plastic-degrading bacteria as follows:

$$F_{(ER)} = \sum_{i=1}^{n} Nor(F_{(ER)}i) \quad (3)$$

Finally, in combination with the above three factors, we calculated the priority index (PI) of the candidate plastic-degrading bacteria as follows:

$$PI_{MAG} = DP \times 1/2F_{(AC)} \times 1/2F_{(CC)} \times F_{(ER)} \quad (4)$$

where DP is the count of putative plastic-degrading enzyme-encoding in each candidate plastic-degrading bacteria. High plastic degradation potential, no other risk burden to human beings, and the environment play key roles in the selection of candidate plastic-degrading bacteria. Accordingly, these were considered primary indicators in our computing framework (weight 100%), whereas the weights of the adaptation and competitive capacity indicators were reduced (50%), reflecting the reduced importance of these two factors. The priority index of the pathogen is directly set to 0. The K-means algorithm divided the candidate plastic-degrading bacteria (number not strictly even but dissimilar in each rank) into EXCELLENT (n = 11), HIGH (n = 50), MEDIUM (n = 186), and LOW (n = 1,497).

Enrichment and isolation of plastic-degrading bacteria

We deployed samples of PLA and PET plastic for *in situ* enrichment in Xiazhu

Lake, Huzhou, China (30.51581° N, 120.04847° E), for six months and subsequently retrieved these plastics for collecting the PLA and PET plastisphere samples. Each plastic sample was rinsed three times with deionized water to remove any unattached organisms. Plastisphere samples were then collected via a sterile brush and blade, transferred into sterile centrifuge tubes containing 10 mL of PBS, and vortexed for 5 minutes. The plastic debris was removed from the resulting solution, and the remaining suspension was used as a microbial inoculum. The inoculum was transferred to a 250-mL flask containing 0.1 g of pre-cleaned PLA and PET particles (irregularly spherical, diameter of 120–200 μm) and 100 mL of MSM-based customized liquid medium. The test plastics were purchased from Dongguan Kaixuan Plastic Technology Co., Ltd., sterilized in 75% ethanol for 20 minutes, washed three times with sterile water, and air-dried on a sterile workbench. After weighing approximately 0.1g of each sample, the particles were placed in small Petri dishes and dried in a laminar flow hood. Subsequently, they were sterilized under UV light for 3 hours. After incubating at 28°C for 3 days, no colonies were observed around the particles in the culture medium, making them suitable for use in subsequent experiments.

To isolate plastic-degrading microorganisms, the plastic sheets were cut into 2 cm × 2 cm pieces, which were then suspended in 10 mL PBS solution and vortexed for 5 minutes. After removing the plastic pieces from the mixture, the remaining suspension was used as the inoculum and transferred into a 250 mL flask containing approximately 0.1g of pre-treated PET and PLA particles and 100 mL mineral salt medium (MSM). The experimental group was supplemented with eight different specialized media containing various growth factors following the recommended dosages of each essential growth factor (Dataset S9),[28,29] while the control group consisted of MSM without added growth factors. The PET and PLA particles were sterilized in 75% ethanol for 20 minutes, washed three times with sterile water, and dried in a laminar flow hood. Before the experiment, the sterilized PET and PLA particles were weighed. The microorganisms were cultured for 60 days at 30 °C in a shaking incubator (150 rpm), with subculturing every 15 days. A 1 mL aliquot of the

mixed bacterial culture was transferred into 5 mL Luria-Bertani medium and incubated for 24 hours. Then, 100 µL of the Luria-Bertani culture was transferred into a 1.5 mL sterile centrifuge tube containing sterile water, mixed, and used to prepare serial dilutions (10^{-1}, 10^{-2}, 10^{-3}, 10^{-4}, 10^{-5}, and 10^{-6}) to reduce the bacterial concentration. An appropriate volume of the diluted solution was inoculated onto agar plates using sterile pipette tips, and the bacteria were spread using sterile glass beads. The inoculated plates were incubated at an appropriate temperature (30 °C) for 24 hours, and bacterial colony growth was observed. Colonies that were consistent in appearance, color, and size (e.g., shape, size, and color) were selected as independent single colonies. A total of 53 independent colonies were recorded and then subcultured onto fresh agar plates, repeating the process 2-3 times to ensure the isolation of pure colonies (Figure S15).

Whole-genome sequencing of an isolated plastic-degrading strain

High-molecular-weight genomic DNA was extracted from late-log-phase cultures using the Cetyl Trimethyl Ammonium Bromide method with lysozyme digestion (10 mg/mL, 37 °C, 1 h). DNA quality was verified by Nanodrop (OD260/280 > 1.8) and Qubit fluorometry (Thermo Fisher). Illumina libraries (insert size: 394–441 bp) were constructed using the NEBNext Ultra II DNA Library Prep Kit and sequenced on the Illumina NovaSeq 6000 platform (Majorbio BioPharm Technology Co., Ltd., Shanghai) with 150-bp paired-end reads targeting ≥100× coverage. Raw reads were quality controlled using FASTP v0.20.0 (parameters: -q 20 -u 30 -l 50).[30] De novo assembly was performed using SOAPdenovo v2.04 (kmer = 55) with GapCloser v1.12 for scaffold refinement [1]. Assembly quality was assessed via BUSCO v5.4.7 (Bacteria odb10 dataset; completeness > 98%).[31] For gene prediction, Prodigal v2.6.3 was used, and for tRNA/rRNA identification, Barrnap (v.0.9) and tRNAscan-SE (v.2.0.9) with default parameters were used.[10] The database and thresholds for function annotation are consistent with those in the section "Annotation of functions in LPDMAGs and reference genomes". The whole-genome features of *Serratia ficaria* HfyG-1 were shown in Figure S19.

Full-length 16S rRNA sequencing and bioinformatics analysis

For the PLA and PET degrader isolations, culture broths collected on Day 30 and Day 60 were used for DNA extraction with the FastDNA® Spin Kit for Soil (MP Biomedicals) following the manufacturer's protocol. DNA purity and concentration were assessed with a Qubit fluorometer. The nearly full-length 16S rRNA gene was amplified using primers 27F (5′-AGAGTTTGATCMTGGCTCAG-3′) and 1492R (5′-TACGGYTACCTTGTTACGACTT-3′), and libraries were subsequently sequenced on the Majorbio Cloud Platform (Shanghai, China). Raw reads were processed with the VSEARCH workflow: quality filtering, dereplication, chimera removal (UCHIME), and OTU clustering (typically at 97% sequence identity), yielding representative sequences and an OTU table. Taxonomic annotation was performed against the SILVA reference database (release 138), and the resulting species/OTU table was used for downstream community analyses.

Verification test of plastic degradation

Plastisphere samples from Xiazhu Lake in Huzhou, China, were cultured for 60 days using PLA and PET as the sole carbon sources. The type4 cultured bacterial community, which was the most significantly enriched, was used to verify the degradation ability of plastics. Subsequently, the cleaned PLA and PET particles were inoculated with the type4 cultured bacterial community and *S. ficaria* HfyG-1 strain solution (four treatment groups, three biological replicates) and sterile water (four control groups, three biological replicates) for a duration of 90 days. After 90 days, the PLA and PET particles were washed three times with sterile water and air-dried in a sterile environment. The weight of each plastic sample was then recorded. Weight loss, the most straightforward indicator of plastic degradation, was calculated via Equations (5) and:[31]

$$\text{Weight loss} = \frac{W_{initial(g)} - W_{post(g)}}{W_{initial(g)}} \times 100\% \quad (5)$$

where $W_{\text{initial(g)}}$ represents the initial weight and $W_{\text{post(g)}}$ represents the weight after treatment. Three pieces of plastic were weighed and averaged.

Furthermore, SEM images provide a close-up view of the plastic film surfaces; atomic force microscopy was used to observe the plastic surface topographical

changes and calculate the roughness.[32] Fourier transform infrared spectroscopy was used to assess the degradation of plastics by measuring the absorption of infrared radiation at specific wavelengths, which corresponds to the vibration modes of the chemical bonds.

Statistical analysis and visualization

The data are presented as the means ± standard errors of the means. We tested for differences via multiple methods, as described in the main text. The Mann–Whitney U test and Kruskal–Wallis test were used for pairwise comparisons between two or more groups via GraphPad Prism 8. Principal coordinate analyses and Adonis tests were performed via R (v3.6.3), and heatmaps were generated via TBtools (v1.113) and R (v3.6.3). The classification and regression models were built, and the accuracy was assessed *via random forest regression* and *forest.score* functions in the 'sklearn' Python package (v1.1.3). Venn diagrams were generated via EVenn (http://www.ehbio.com/test/venn; v1.0). Data figures were created via GraphPad Prism 8 and R (v3.6.3), and Adobe Illustrator 2020 was used to format the figures. The phylogeny was built via the 400 universal PhyloPhlAn markers via PhyloPhlAn (v3.0.60),[33] FastTree (v2.1.10),[34] and RAxML (v8.1.12) with parameters.[35] The best tree refined by RAxML is generated via ggtree (v3.2.1) and iTOL (v6.8.1).[36] All schematic diagrams and art elements were drawn via BioRender (https://app.biorender.com/; v1.0) with full publishing rights.

Supplemental figures

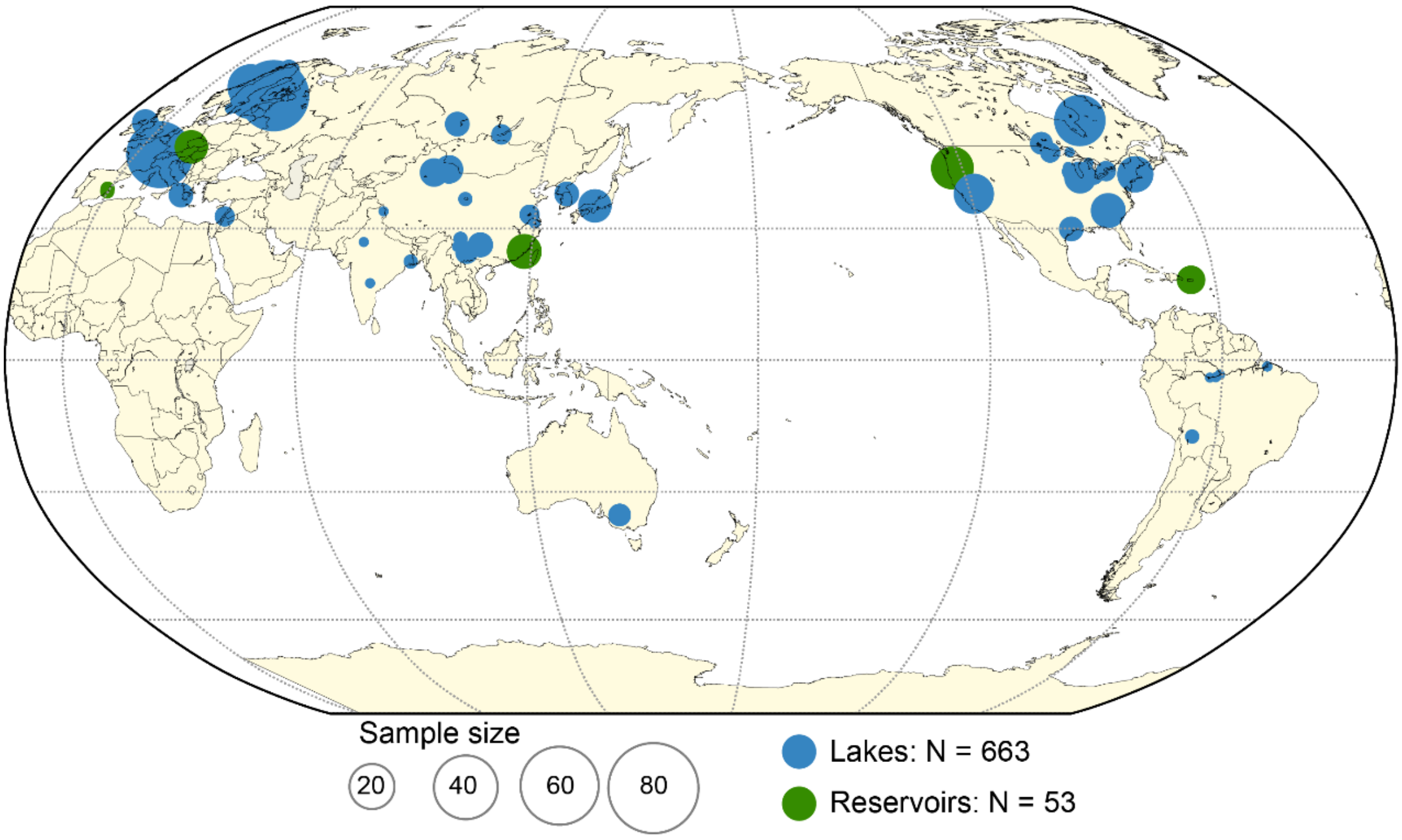

Figure S1. Geographical distribution of metagenomic sequencing data Circle size indicates sample numbers. Blue and green circles indicate the lake and reservoir samples, respectively. More details can be found in Dataset S1.

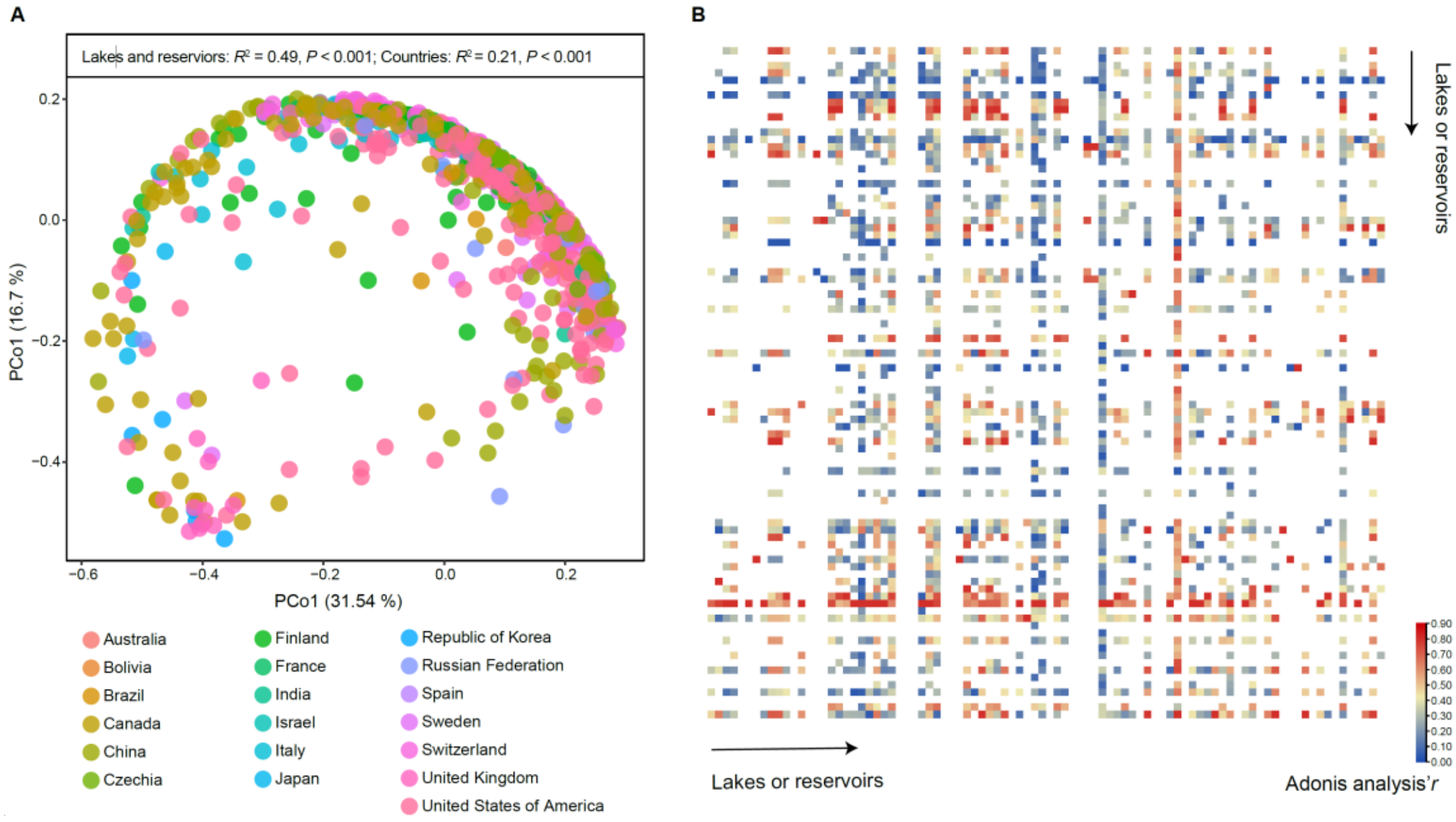

Figure S2. Geographical distribution of microbial plastic-degrading potential of all samples (A) Principal co-ordinates analysis with Bray-Curtis distance showed higher variations of microbial plastic-degrading potential between lakes and reservoirs than countries. (B) Pairwise Adonis analysis showed the distribution of candidate plastic-degrading enzyme-coding genes significantly different among all lakes and reservoirs. The gradient colour scale from red to blue indicates the Adonis analysis' *r* from high to low.

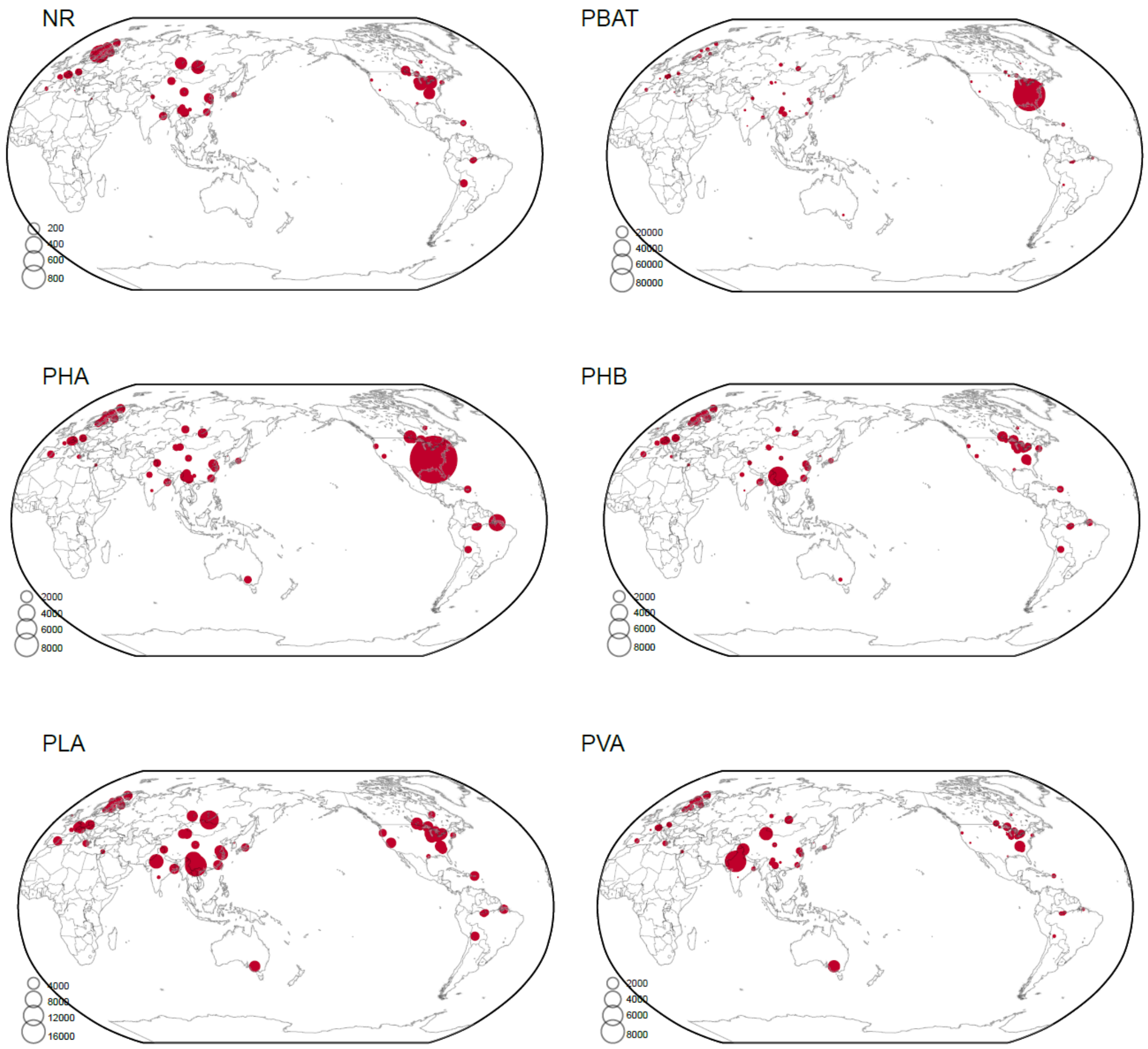

Figure S3. Geographical distribution of biodegradable plastic-degrading potential of microbes in lakes NR, natural rubber; PBAT, polybutylene adipate terephthalate; PHAs, poly-hydroxyalkanoate; PHB, polyhydroxybutyrate; PLA, polylactic acid; PVA, polyvinyl alcohol. The size of the circle indicates the abundance of candidate plastic-degrading enzyme-coding genes.

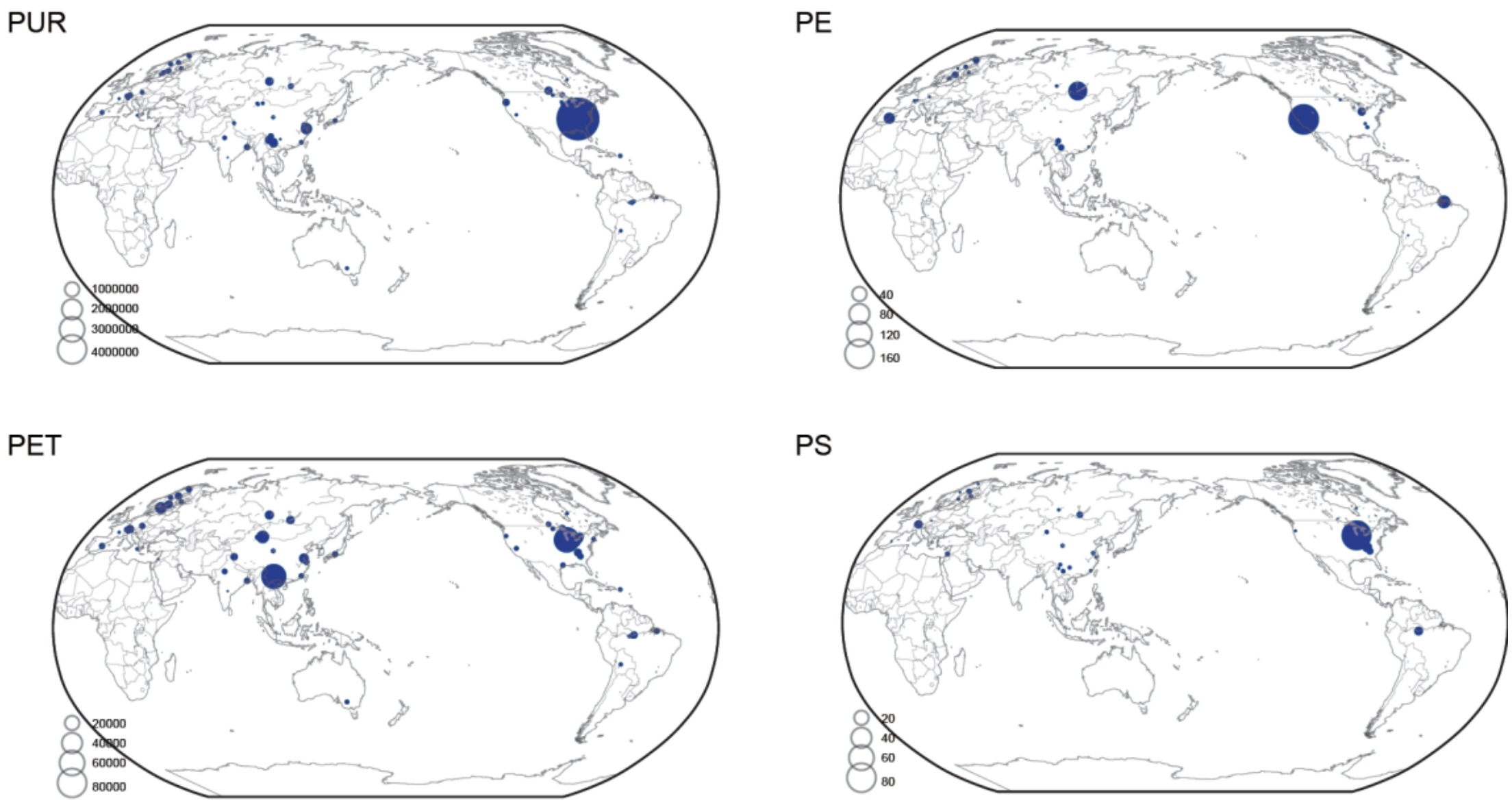

Figure S4. Geographical distribution of plastic-degrading potential of microbes in polyolefin and conventional polyester plastics in lakes The size of the circle indicates the abundance of candidate plastic-degrading enzyme-coding genes. PUR: polyurethane; PET: polyethylene terephthalate; PS: polystyrene; PE: polyethylene.

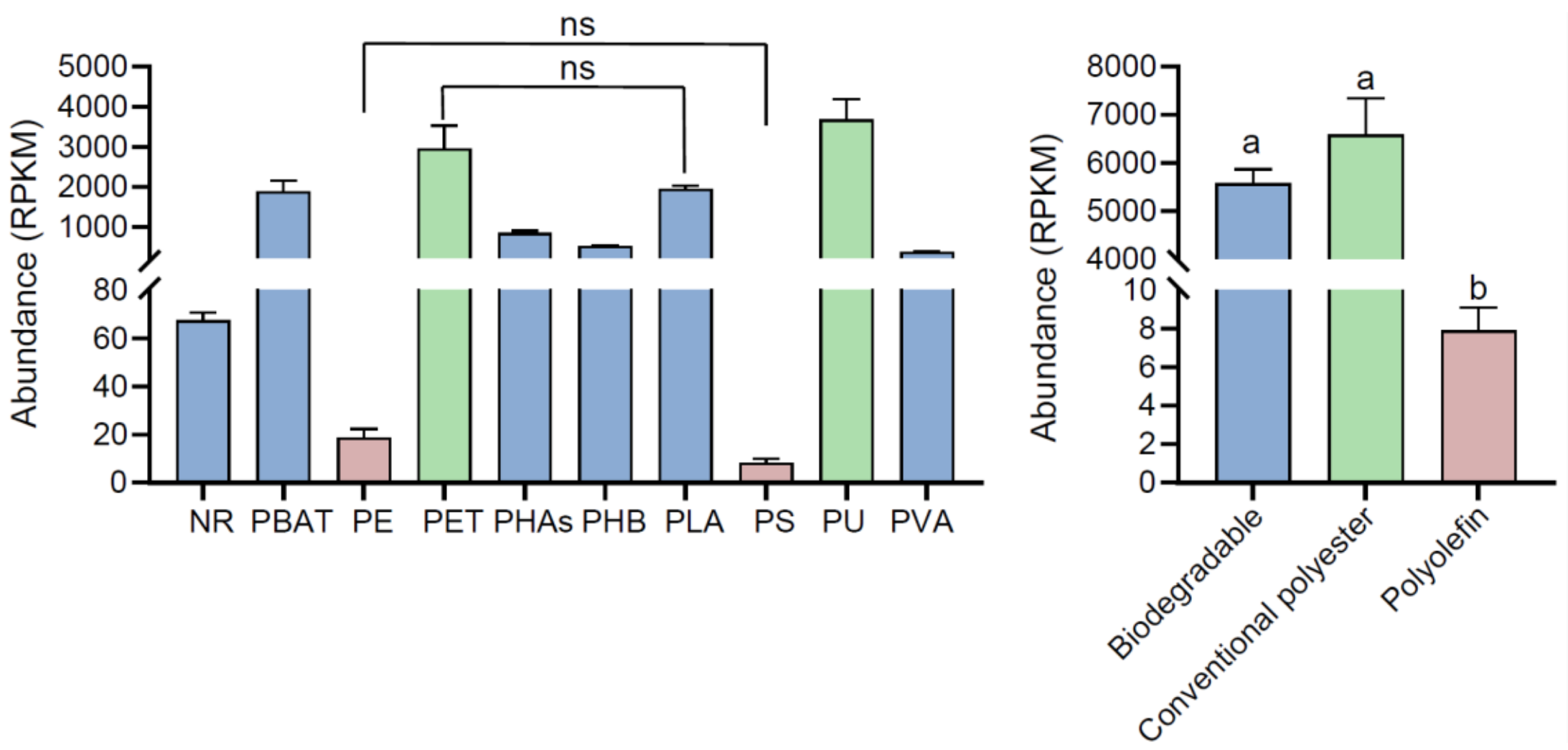

Figure S5. The abundance of plastic-degrading potential in lakes Abundance (RPKM) of various candidate plastic-degrading enzyme-coding genes. Different letters indicate the significant difference among groups (Kruskal-Wallis test, $p < 0.05$). RPKM: Reads Per Kilobase per Million mapped reads. Data are represented as mean ± SEM.

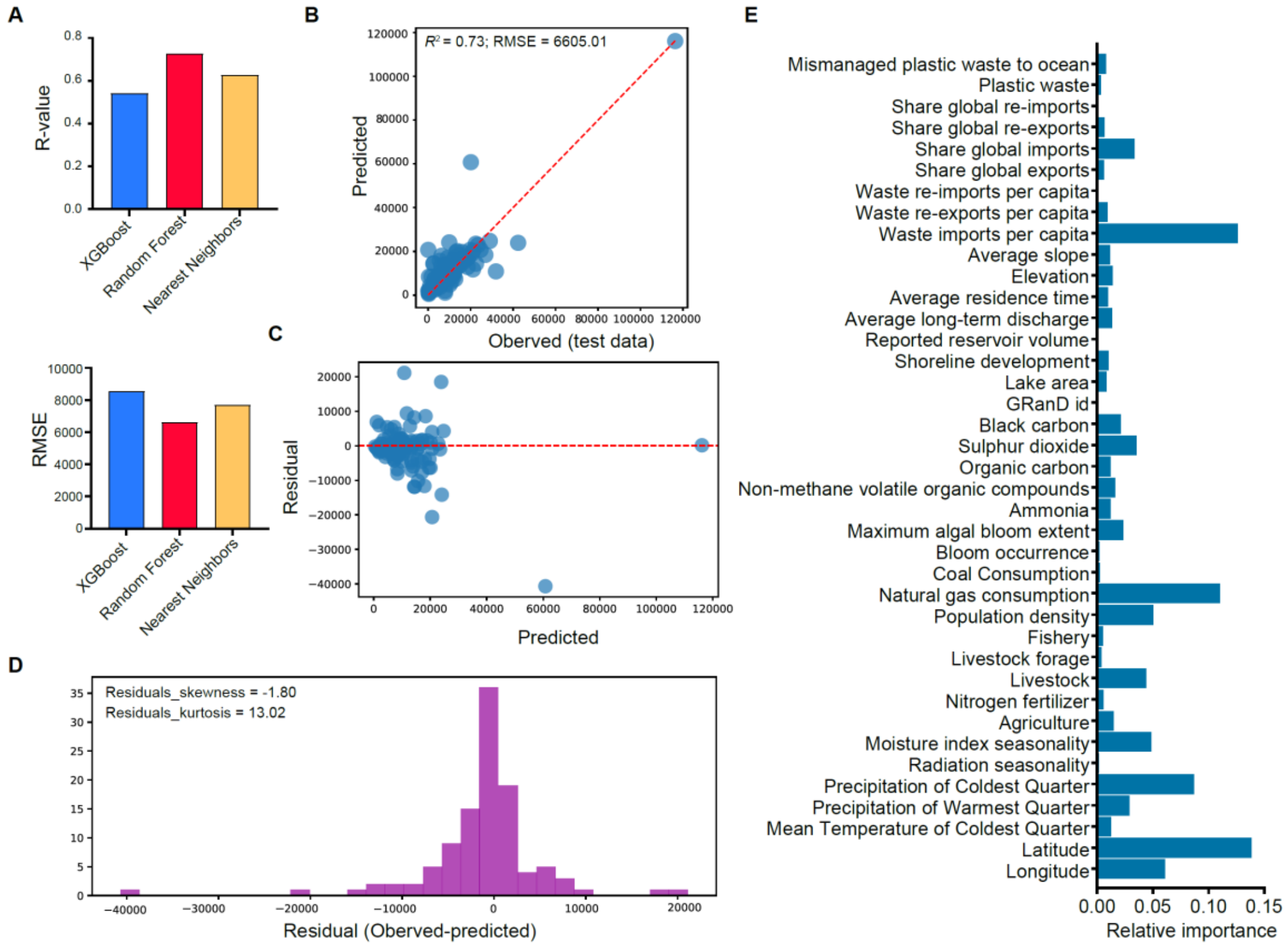

Figure S6. Machine learning prediction of microbial plastic-degradation potential in global lakes (A) Model performance comparison among XGBoost, Random Forest, and K-Nearest Neighbors algorithms based on R^2 and RMSE values. (B) Observed versus predicted plastic-degradation potential in the test dataset using the XGBoost model (R^2 = 0.73; RMSE = 6605.01). (C) Residual plot showing the distribution of prediction errors across samples. (D) Histogram of residuals illustrating skewness and kurtosis values. (E) Relative importance of 39 environmental and anthropogenic predictors in shaping microbial plastic-degradation potential, highlighting the dominant influence of plastic-related and climatic variables.

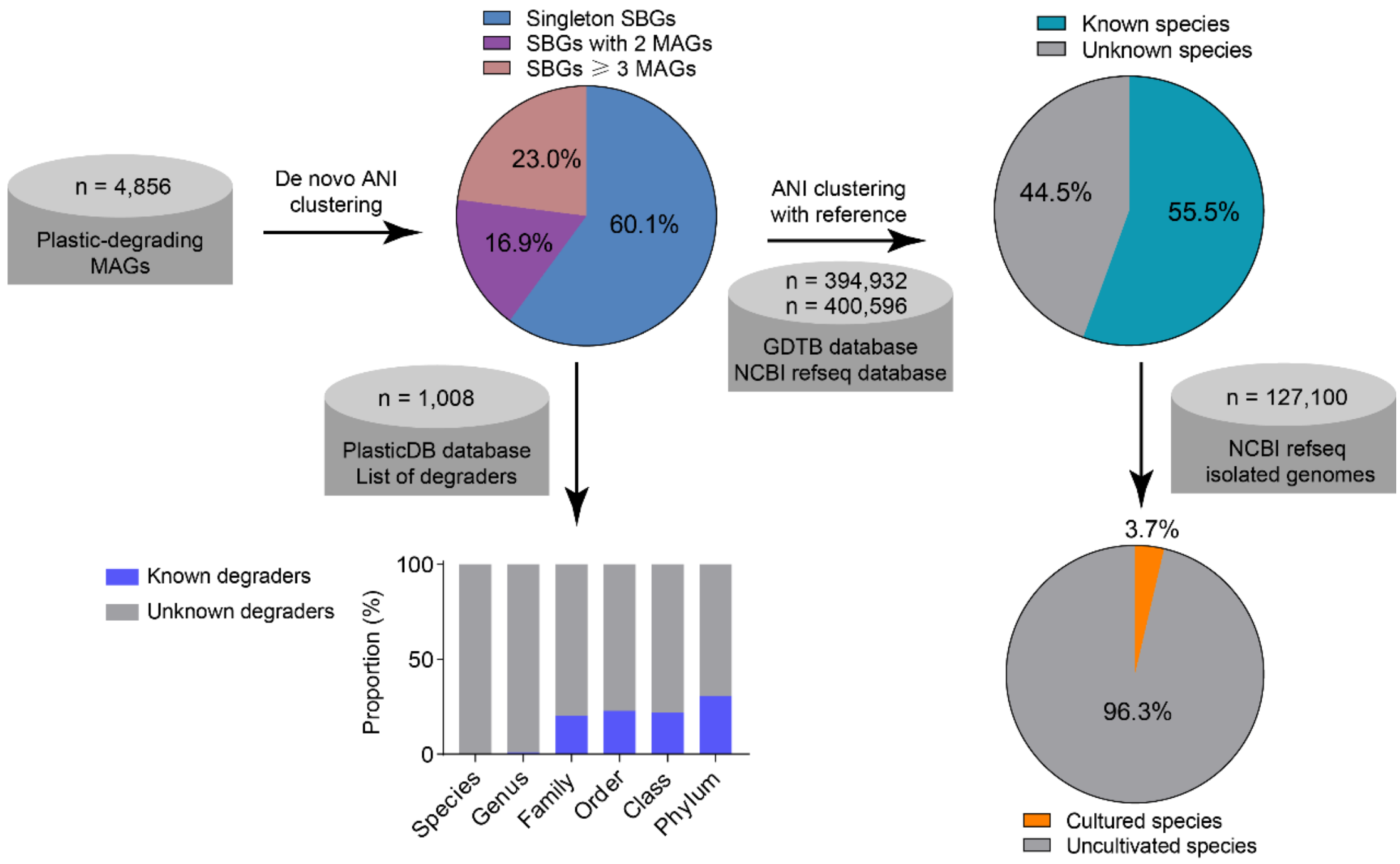

Figure S7. 1,370 genomes (41%) from 4,856 candidate plastic-degrading MAGs were assigned to the plastic degraders We clustered the 4,856 MAGs into 1,370 species-level genome bins (SBGs, denoted as plastic degraders) based on 95% whole-genome average nucleotide identity (ANI).

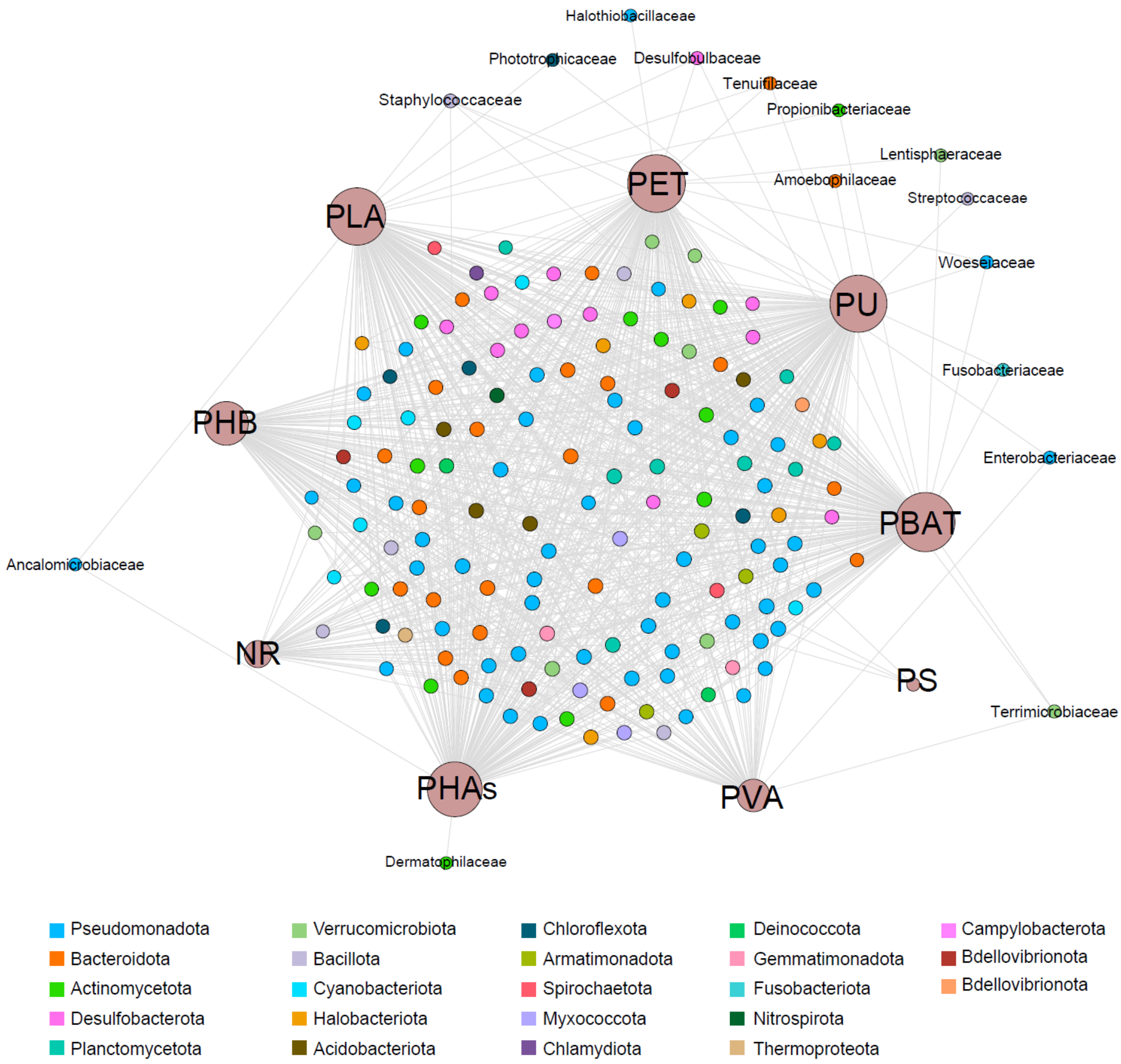

Figure S8. Taxonomic network linking microbial families and plastic types Network visualization shows the associations between candidate plastic-degrading microbial families and ten representative plastic polymers (PLA, PET, PU, PBAT, PHAs, PHB, PVA, PS, NR). Each node represents a microbial family, colored by its corresponding phylum, and edges indicate shared degradation potential inferred from enzyme annotation and co-occurrence patterns. Larger nodes denote plastic types, and node proximity reflects functional similarity in degradation profiles. The network reveals extensive taxonomic overlaps across polymers, suggesting broad enzymatic versatility among plastic-degrading lineages.

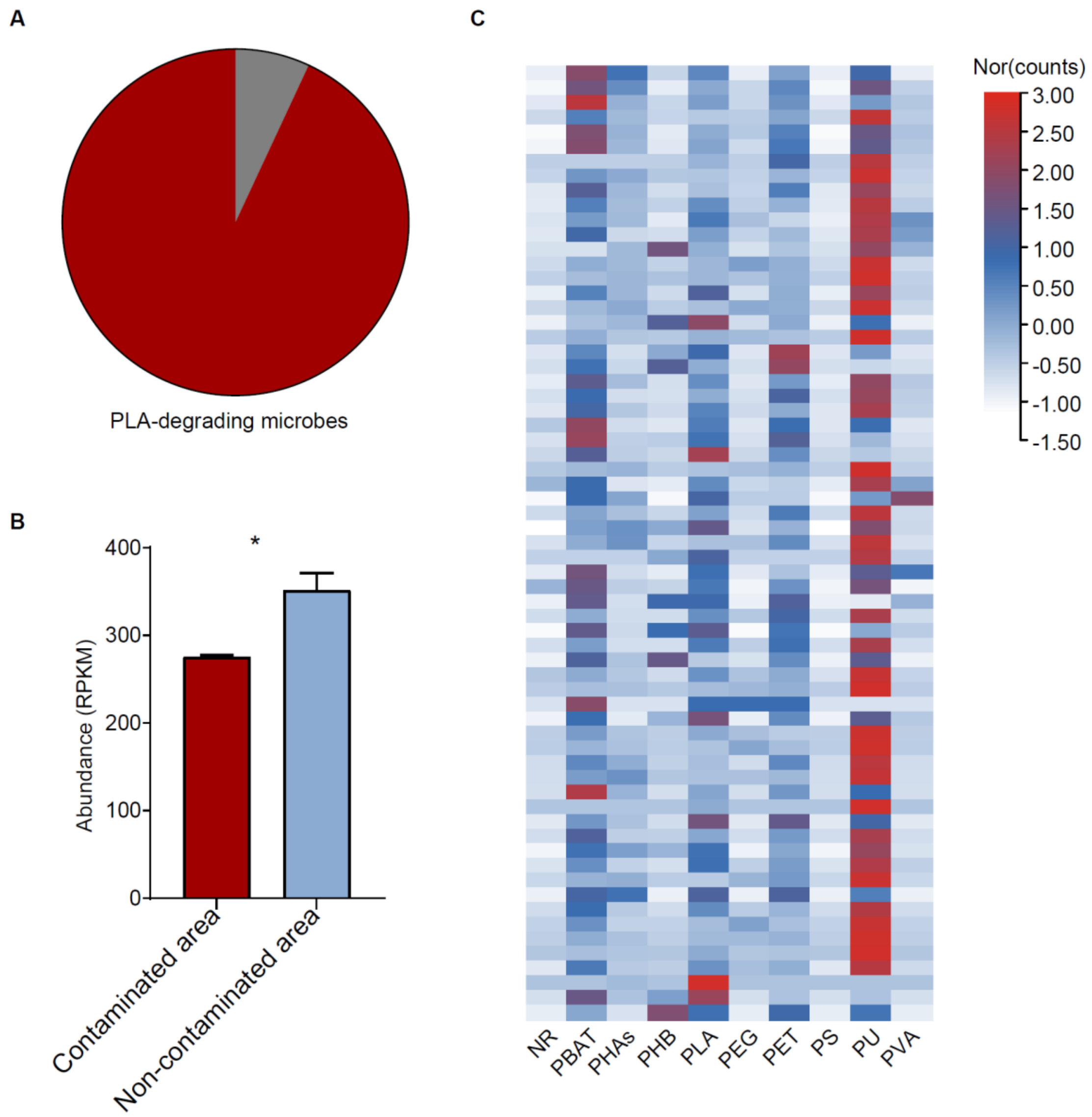

Figure S9. Distribution and functional versatility of candidate PLA-degrading microbes in contaminated versus non-contaminated areas (A) Proportion of candidate PLA-degrading microbes among all candidate degraders detected in the PLA-contaminated site, showing that 93% were PLA degraders. (B) Abundance (RPKM) of candidate PLA-degrading microbes in contaminated and non-contaminated areas, with significantly higher abundance in the contaminated site. Data are represented as mean ± SEM. "*" indicates significance at $P < 0.05$ (one-sided t-test). (C) Heatmap of normalized counts of enzymes encoded by candidate PLA-degrading microbes, showing their functional repertoire across different plastic types.

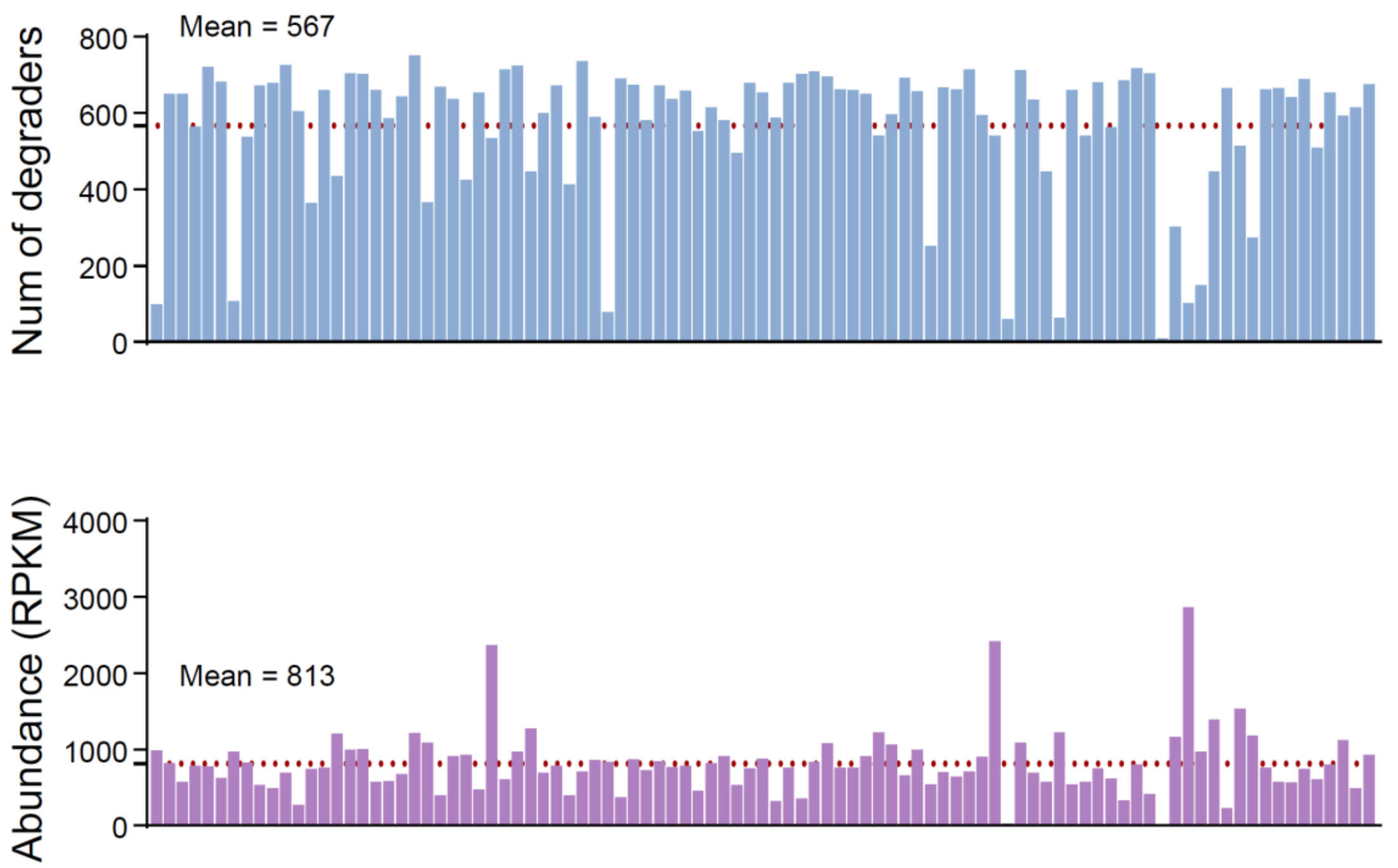

Figure S10. Number and abundance of candidate plastic-degrading bacteria in lake metagenomes We detected an average of 567 candidate plastic-degrading taxa per sample, with a mean abundance of 813 (RPKM).

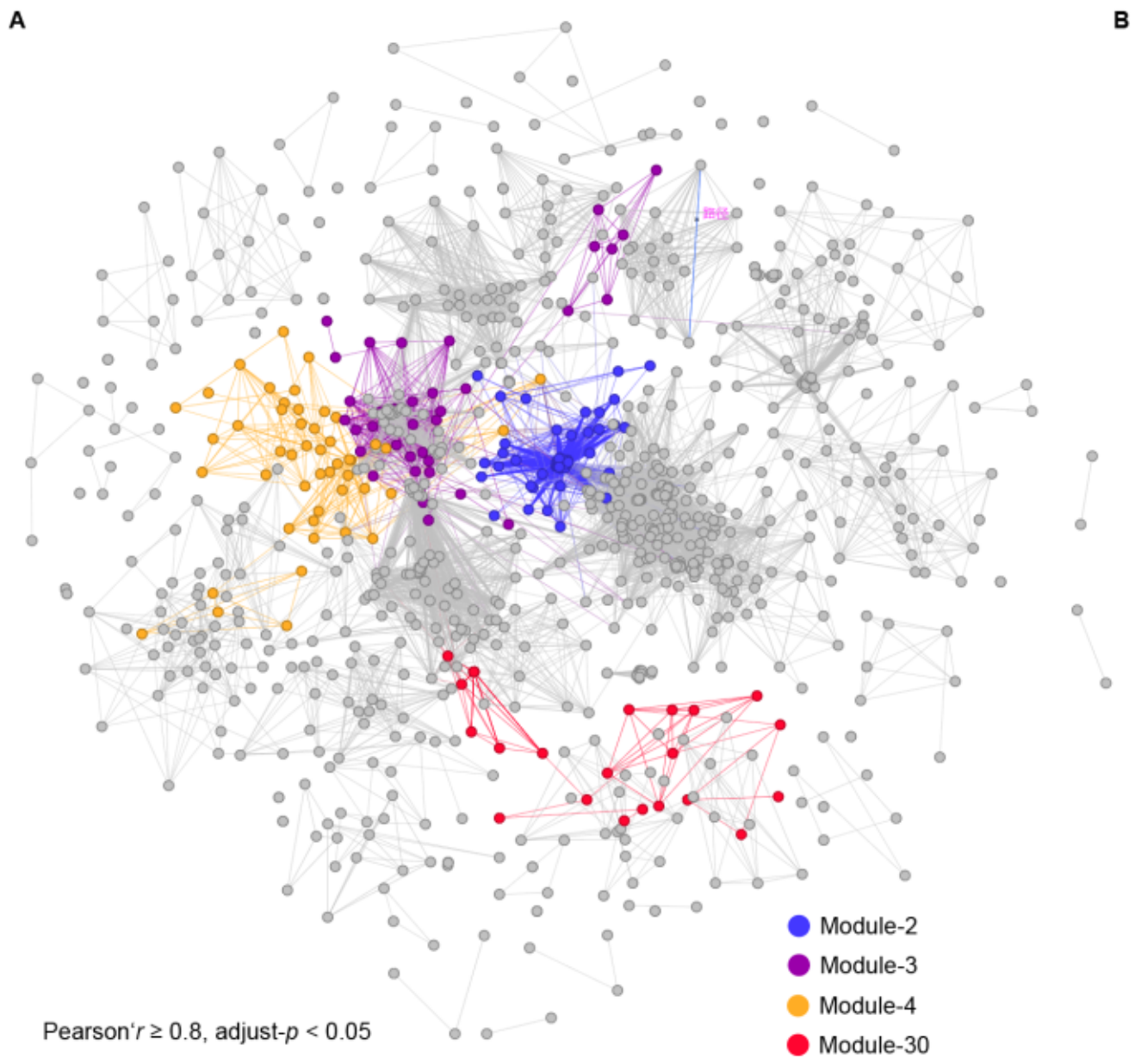

Figure S11. Co-occurrence modules associated with candidate plastic-degrading bacteria in lake metagenomes (A) Network showing microbial taxa significantly correlated with candidate plastic-degrading bacteria (Pearson's $r \geq 0.8$, adjusted $p < 0.05$). Colored nodes represent four major modules. (B) Taxonomic composition of the four modules, highlighting dominant non-degrading taxa potentially cooperating with degraders in nutrient cycling and metabolic interactions.

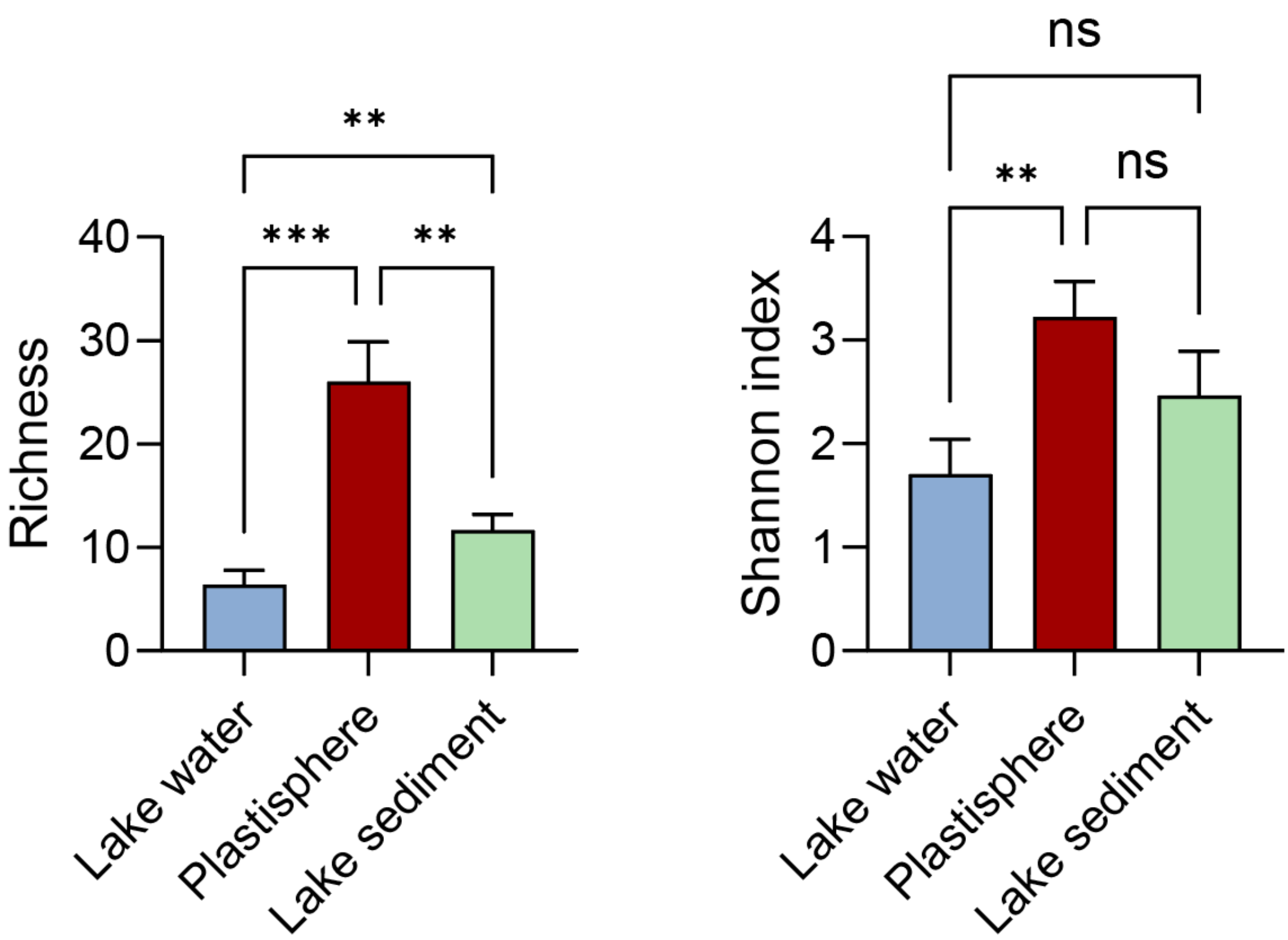

Figure S12. Diversity of candidate plastic-degraders in plateau karst lakes The plastisphere in lakes is a hot zone for enriching and isolating plastic-degrading bacteria. Data are represented as mean ± SEM. "**"and "***" indicate significance at $P < 0.01$ and $P < 0.001$, respectively (one-way ANOVA with Bartlett's test).

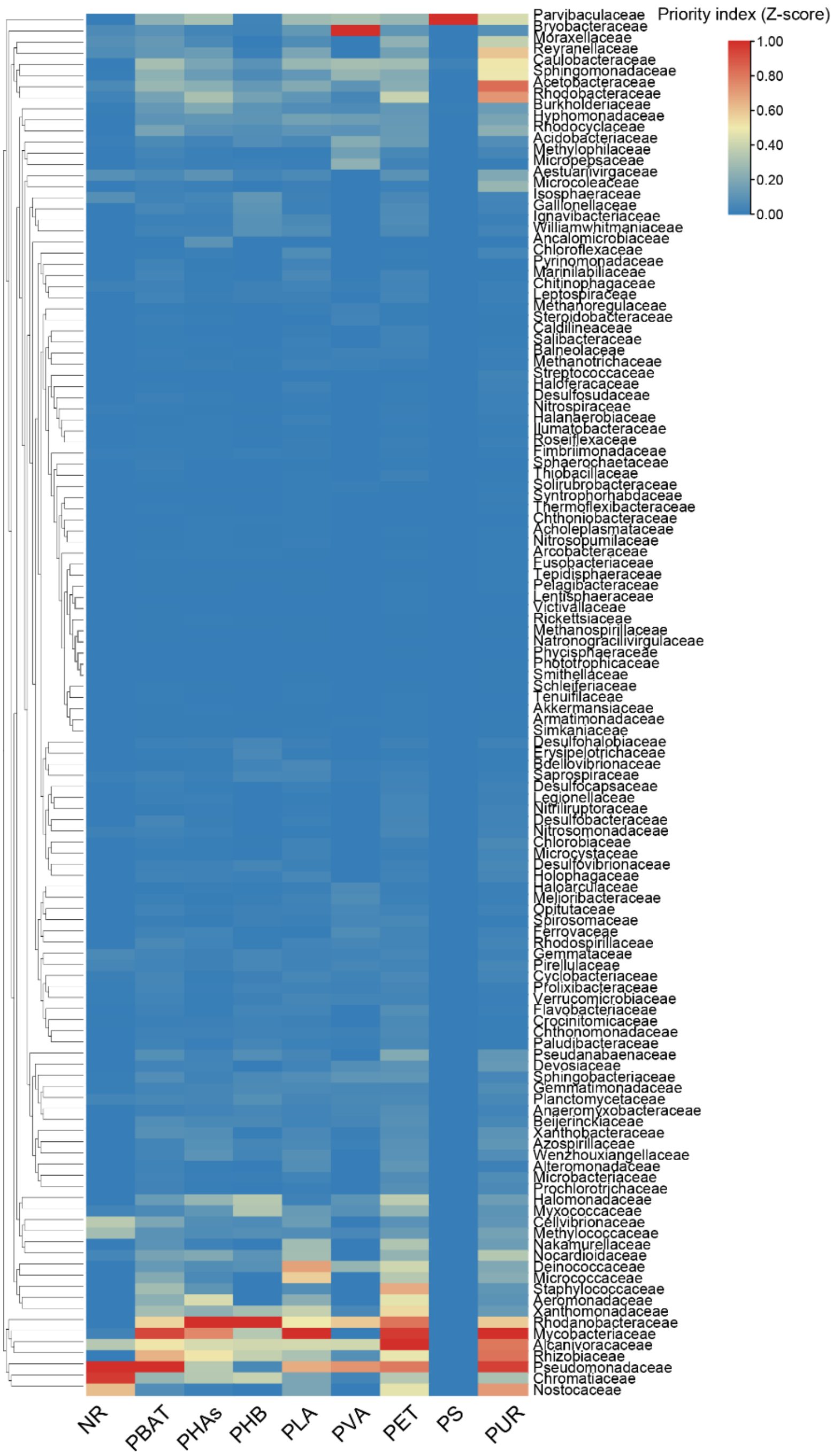

Figure S13. The priority index of candidate degraders in various plastics Color gradient indicates the Z-score of priority index from high (red) to low (blue).

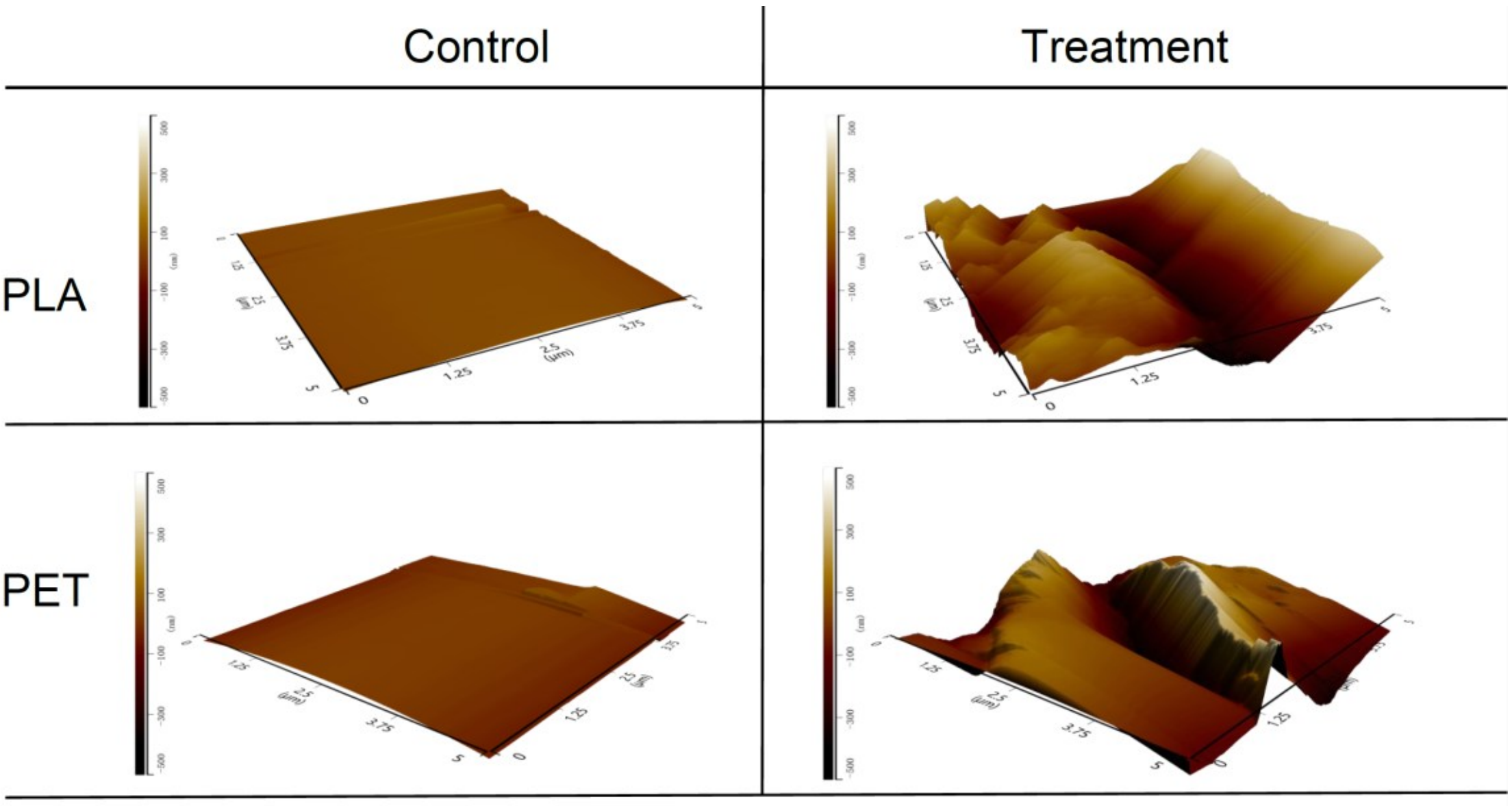

Figure S14. The topography of plastic surface Topographic map of a 5*5 μm microregion shows that the roughness of plastic surface was significantly increased in treatment groups, especially the PLA and PET.

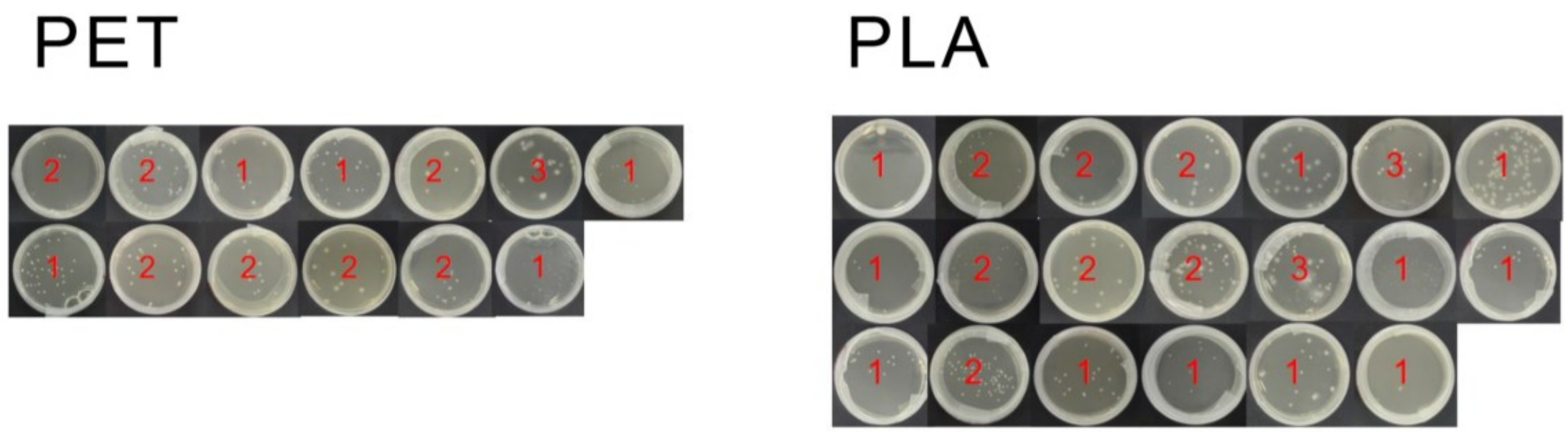

Figure S15. Candidate plastic-degrading bacterial colonies A total of 53 candidate plastic-degrading bacterial colonies were isolated from eight customized media containing PLA and PET particles.

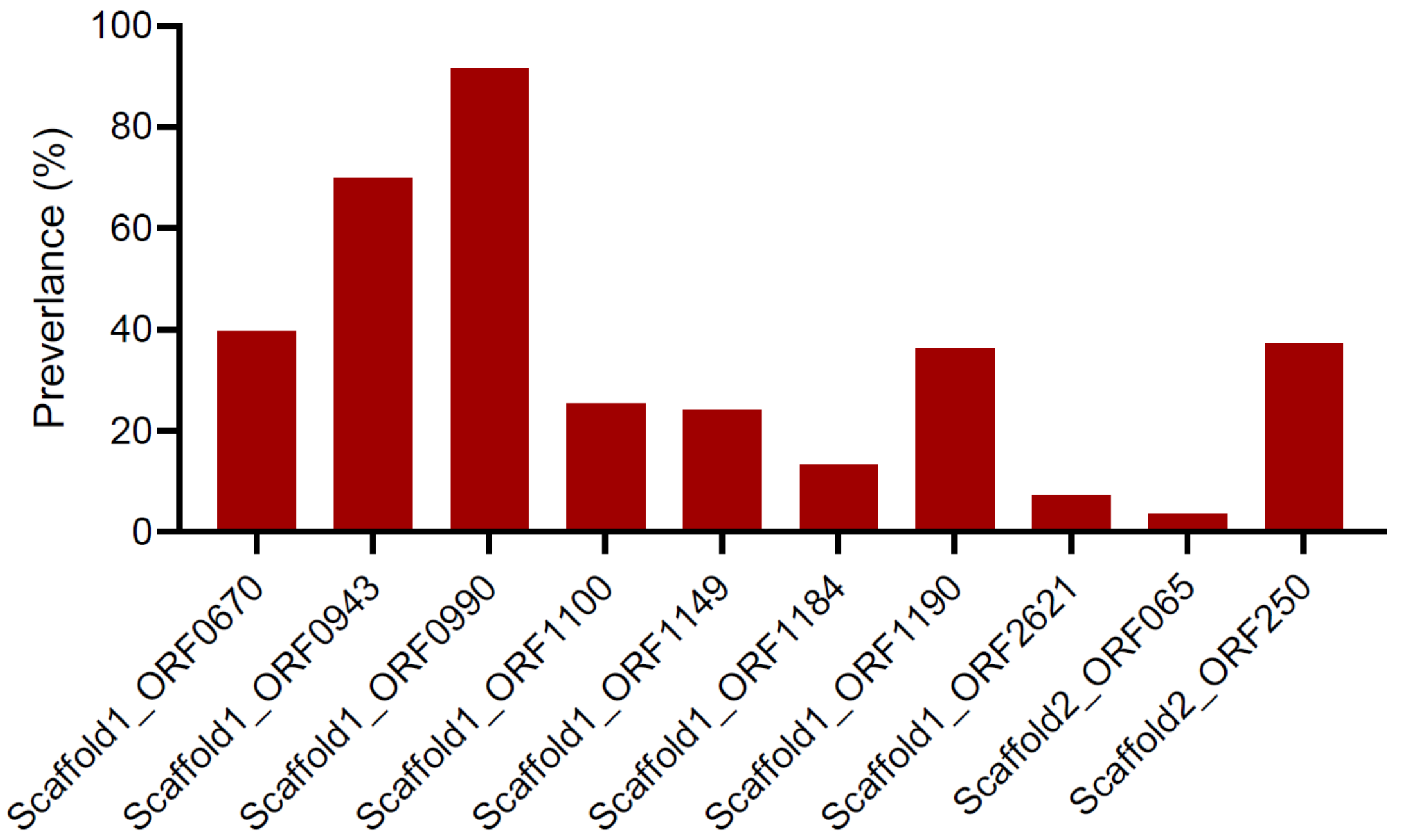

Figure S16. The detection rate of putative plastic-degrading enzymes in the lakes

Metatranscriptomic surveys showed that these putative enzymes were frequently detected, suggesting they are actively expressed in natural environments and thus likely to play functional roles in plastic degradation *in situ*.

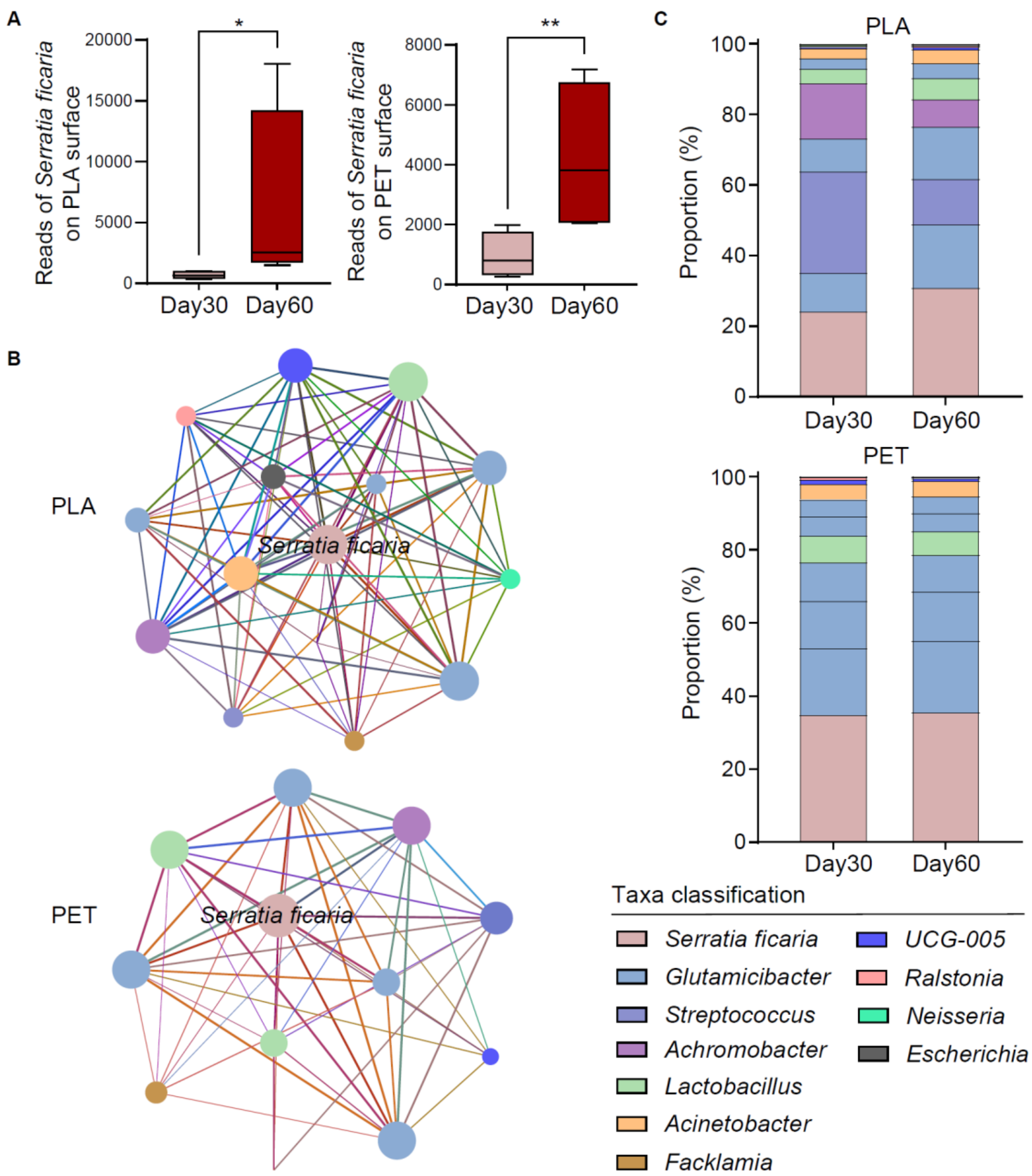

Figure S17. Synergistic microbial interactions during PLA and PET degradation

(A) Temporal enrichment of *Serratia ficaria* on PLA and PET surfaces during 30- and 60-day incubation, showing a significant increase in relative abundance over time. Data are represented as mean ± SEM. “*”and “**” indicate significance at $P < 0.05$ and $P < 0.01$, respectively (one-sided *t*-test). (B) Co-occurrence networks of microbial consortia associated with *S. ficaria* on PLA and PET surfaces. Node size represents relative abundance, and edge color indicates positive correlations among taxa. (C) Community composition at the genus level, showing the increasing dominance of *S. ficaria* and its cooperative taxa (e.g., *Glutamicibacter*, *Achromobacter*, *Ralstonia*, *Lactobacillus*) over time.

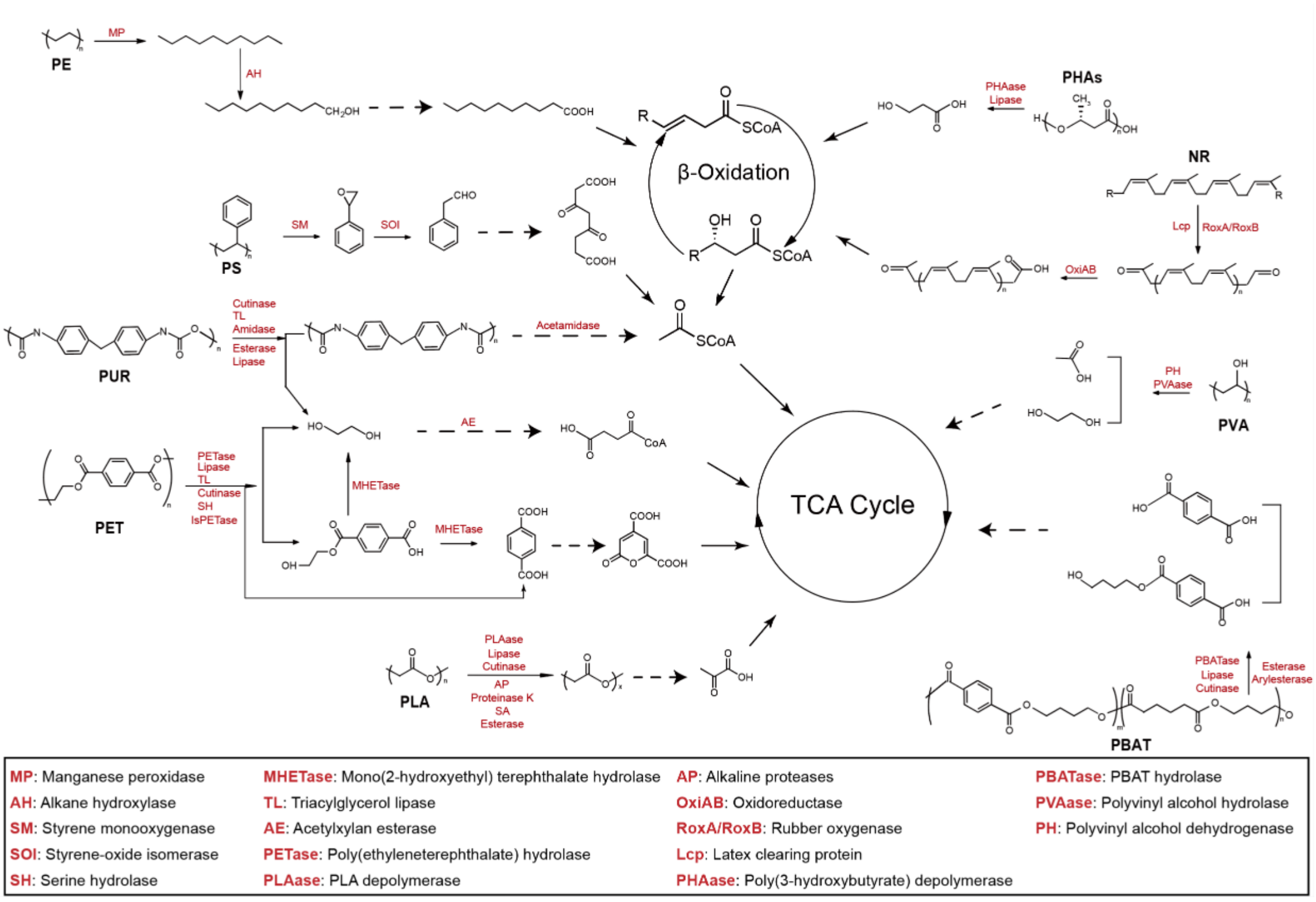

Figure S18. Biodegradation pathways of various plastics PVA, polyvinyl alcohol; PHA, poly-hydroxyalkanoate; PHB, polyhydroxybutyrate; PUR, polyurethane; PET, polyethylene terephthalate; PBAT, polybutylene adipate terephthalate; PS, polystyrene; PLA, polylactic acid; PE, polyethylene; NR, natural rubber. The degradation pathways and enzymes of PS and PE are still in the exploratory stage in the current research, and some enzymes may not be widely verified.

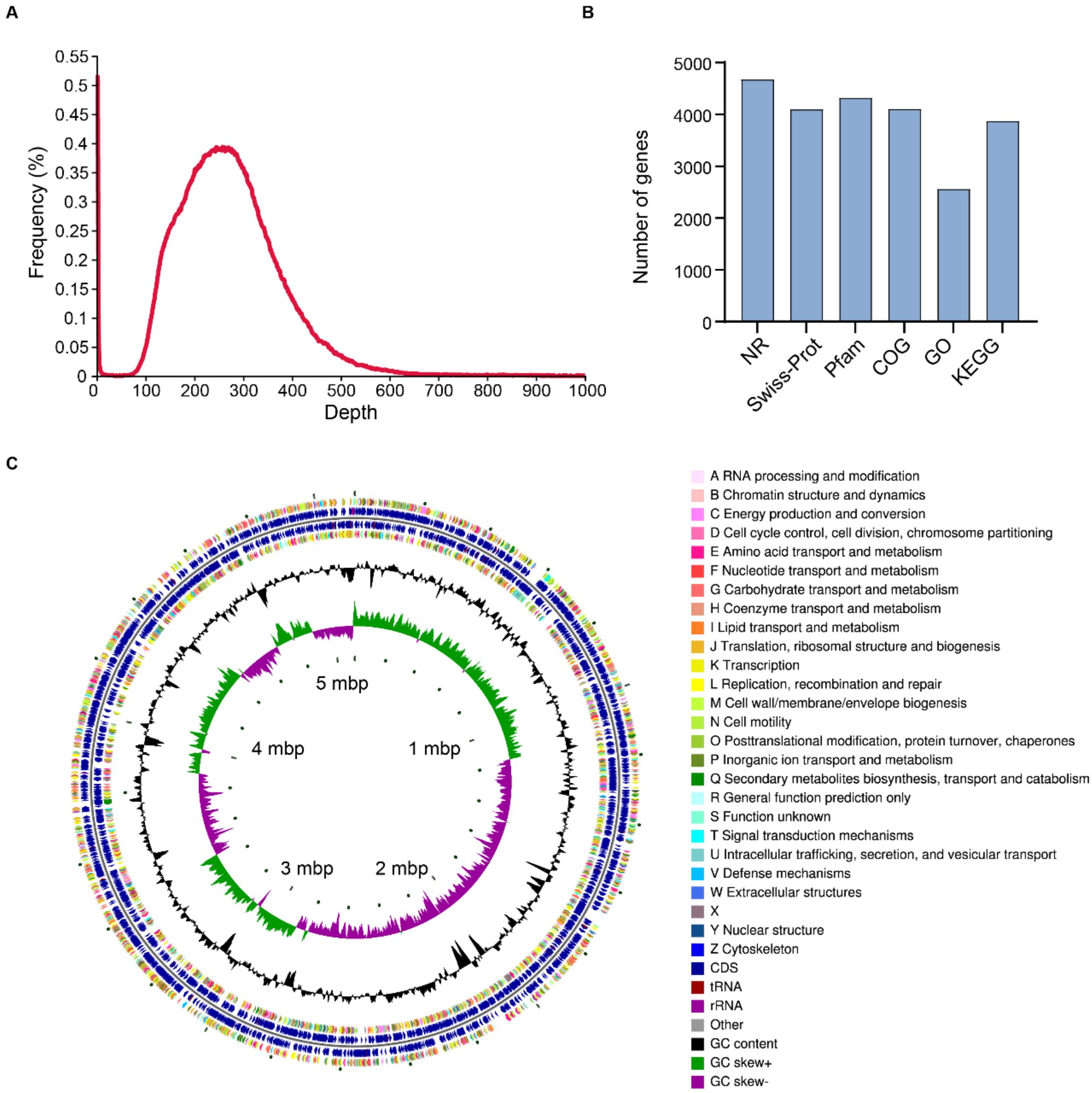

Figure S19. Whole-genome features of *Serratia ficaria* HfyG-1. (A) K-mer frequency distribution showing a peak at ~25× sequencing depth, indicating uniform coverage. (B) Functional annotation counts across databases: NR (highest), Swiss-Prot, COG, GO, and KEGG. (C) Circular genome map (4 Mbp scale) displaying: Outer ring: Functional COG categories (A: RNA processing; S: Unknown function largest segment). Middle rings: Gene features (CDS/tRNA/rRNA). Inner rings: GC content (black) and GC skew (±, blue).

Supplemental references

1. Li D., Liu C. M., Luo R., et al. (2015). MEGAHIT: an ultra-fast single-node solution for large and complex metagenomics assembly via succinct de Bruijn graph. *Bioinformatics* **31**:1674-1676. DOI:10.1093/bioinformatics/btv033
2. Langmead B. and Salzberg S. L. (2012). Fast gapped-read alignment with Bowtie 2. *Nat. Methods* **9**:357-359. DOI:10.1038/nmeth.1923
3. Li H., Handsaker B., Wysoker A. et al. (2009). The Sequence Alignment/Map format and SAMtools. *Bioinformatics* **25**:2078-2079. DOI:10.1093/bioinformatics/btp352
4. Alneberg J., Bjarnason B. S., de Bruijn I. et al. (2014). Binning metagenomic contigs by coverage and composition. *Nat. Methods* **11**:1144-1146. DOI:10.1038/nmeth.3103
5. Wu Y. W., Simmons B. A. and Singer S. W. (2016). MaxBin 2.0: an automated binning algorithm to recover genomes from multiple metagenomic datasets. *Bioinformatics* **32**:605-607. DOI:10.1093/bioinformatics/btv638
6. Kang D. D., Li F., Kirton E. et al. (2019). MetaBAT 2: an adaptive binning algorithm for robust and efficient genome reconstruction from metagenome assemblies. *PeerJ* **7**:e7359. DOI:10.7717/peerj.7359
7. Parks D. H., Imelfort M., Skennerton C. T. et al. (2015). CheckM: assessing the quality of microbial genomes recovered from isolates, single cells, and metagenomes. *Genome Res.* **25**:1043-1055. DOI:10.1101/gr.186072.114
8. Olm M. R., Brown C. T., Brooks B. et al. (2017). dRep: a tool for fast and accurate genomic comparisons that enables improved genome recovery from metagenomes through de-replication. *ISME J.* **11**:2864-2868. DOI:10.1038/ismej.2017.126
9. Aroney S. T. N., Newell R. J. P., Nissen J. N. et al. (2025). CoverM: read alignment statistics for metagenomics. *Bioinformatics* **41**. DOI:10.1093/bioinformatics/btaf147
10. Chan Patricia P., Lin Brian Y., Mak Allysia J. et al. (2021). tRNAscan-SE 2.0: improved detection and functional classification of transfer RNA genes. *Nucleic*

Acids Res. **49**:9077-9096. DOI:10.1093/nar/gkab688

11. Buchholz P. C. F., Feuerriegel G., Zhang H. et al. (2022). Plastics degradation by hydrolytic enzymes: The plastics-active enzymes database-PAZy. *Proteins* **90**:1443-1456. DOI:10.1002/prot.26325
12. Gambarini V., Pantos O., Kingsbury J. M. et al. (2022). PlasticDB: a database of microorganisms and proteins linked to plastic biodegradation. *Database (Oxford)* **2022**. DOI:10.1093/database/baac008
13. Altschul S. F., Gish W., Miller W., et al. (1990). Basic local alignment search tool. *J. Mol. Biol.* **215**:403-410. DOI:10.1016/S0022-2836(05)80360-2
14. Fu L., Niu B., Zhu Z. et al. (2012). CD-HIT: accelerated for clustering the next-generation sequencing data. *Bioinformatics* **28**:3150-3152. DOI:10.1093/bioinformatics/bts565
15. Li W. and Godzik A. (2006). Cd-hit: a fast program for clustering and comparing large sets of protein or nucleotide sequences. *Bioinformatics* **22**:1658-1659. DOI:10.1093/bioinformatics/btl158
16. Katoh K. and Standley D. M. (2016). A simple method to control over-alignment in the MAFFT multiple sequence alignment program. *Bioinformatics* **32**:1933-1942. DOI:10.1093/bioinformatics/btw108
17. Eddy S. R. (1998). Profile hidden Markov models. *Bioinformatics* **14**:755-763. DOI:10.1093/bioinformatics/14.9.755
18. Yu T., Cui H., Li J. C. et al. (2023). Enzyme function prediction using contrastive learning. *Science* **379**:1358-1363. DOI:10.1126/science.adf2465
19. Alcock B. P., Raphenya A. R., Lau T. T. Y. et al. (2020). CARD 2020: antibiotic resistome surveillance with the comprehensive antibiotic resistance database. *Nucleic Acids Res.* **48**:D517-d525. DOI:10.1093/nar/gkz935
20. Buchfink B., Reuter K. and Drost H. G. (2021). Sensitive protein alignments at tree-of-life scale using DIAMOND. *Nat. Methods* **18**:366-368. DOI:10.1038/s41592-021-01101-x
21. Tu Q., Lin L., Cheng L. et al. (2019). NCycDB: a curated integrative database for fast and accurate metagenomic profiling of nitrogen cycling genes.

Bioinformatics **35**:1040-1048. DOI:10.1093/bioinformatics/bty741

22. Yu X., Zhou J., Song W. et al. (2021). SCycDB: A curated functional gene database for metagenomic profiling of sulphur cycling pathways. *Mol. Ecol. Resour.* **21**:924-940. DOI:10.1111/1755-0998.13306
23. Zheng B. J. Zhu Y., Sardans J. et al. (2018). QMEC: a tool for high-throughput quantitative assessment of microbial functional potential in C, N, P, and S biogeochemical cycling. **61**:1451-1462. DOI: 10.1007/s11427-018-9364-7
24. Shi Z., Yin H., Van Nostrand J. D. et al. (2019). Functional Gene Array-Based Ultrasensitive and Quantitative Detection of Microbial Populations in Complex Communities. *mSystems* **4**. DOI:10.1128/mSystems.00296-19
25. Nadell C. D., Drescher K. and Foster K. R. (2016). Spatial structure, cooperation and competition in biofilms. *Nat. Rev. Microbiol.* **14**:589-600. DOI:10.1038/nrmicro.2016.84
26. Du Toit A. (2018). Increasing virulence factors. *Nat. Rev. Microbiol.* **16**:658-658. DOI:10.1038/s41579-018-0091-3
27. Zhang Z., Zhang Q., Wang T. et al. (2022). Assessment of global health risk of antibiotic resistance genes. *Nat. Commun.* **13**:1553. DOI:10.1038/s41467-022-29283-8
28. Atlas R. (2005). Handbook of media for environmental microbiology, second edition. Q. Rev. Biol. 10.1201/9781420037487.
29. Cross K. L., Campbell J. H., Balachandran M. et al. (2019). Targeted isolation and cultivation of uncultivated bacteria by reverse genomics. *Nat. Biotechnol.* **37**:1314-1321. DOI:10.1038/s41587-019-0260-6
30. Chen S., Zhou Y., Chen Y. et al. (2018). fastp: an ultra-fast all-in-one FASTQ preprocessor. *Bioinformatics* **34**:i884-i890. DOI:10.1093/bioinformatics/bty560
31. Seppey M., Manni M. and Zdobnov E. M. (2019). BUSCO: Assessing Genome Assembly and Annotation Completeness. *Methods Mol. Biol.* **1962**:227-245. DOI:10.1007/978-1-4939-9173-0_14
32. Iñiguez M. E., Conesa J. A. and Fullana A. (2018). Recyclability of four types

of plastics exposed to UV irradiation in a marine environment. *Waste Manag.* **79**:339-345. DOI:10.1016/j.wasman.2018.08.006

33. Asnicar F., Thomas A. M., Beghini F. et al. (2020). Precise phylogenetic analysis of microbial isolates and genomes from metagenomes using PhyloPhlAn 3.0. *Nat. Commun.* **11**:2500. DOI:10.1038/s41467-020-16366-7
34. Price M. N., Dehal P. S. and Arkin A. P. (2010). FastTree 2–Approximately Maximum-Likelihood Trees for Large Alignments. *PLoS One* **5**:e9490. DOI:10.1371/journal.pone.0009490
35. Stamatakis A. (2014). RAxML version 8: a tool for phylogenetic analysis and post-analysis of large phylogenies. *Bioinformatics* **30**:1312-1313. DOI:10.1093/bioinformatics/btu033
36. Letunic I. and Bork P. (2021). Interactive Tree Of Life (iTOL) v5: an online tool for phylogenetic tree display and annotation. *Nucleic Acids Res.* **49**:W293-W296. DOI:10.1093/nar/gkab301
