# Supplementary material for: Potential plastic biodegradation in lakes worldwide
Source: Innovation (Camb). 2026 Mar 3;7(8):101338. doi: 10.1016/j.xinn.2026.101338 (PMC13457188; doi:10.1016/j.xinn.2026.101338)
Supplement: Supplementary file 2 [file mmc3.pdf]

ARTICLE | Environment & Ecology

OPEN ACCESS

www.the-innovation.org

Potential plastic biodegradation in lakes worldwide

Qi Zhang,[1,7] Zhenyan Zhang,[1,7] Ziyao Zhang,[2] Guoyan Qin,[2] Mingkang Jin,[5] Bingfeng Chen,[2] Yitian Yu,[3] Tingzhang Wang,[6] Meixia Wang,[6] Tao Lu,[2] Dong Zhu,[3] Li Cui,[3] Haifeng Qian,[1,*] Matthias C. Rillig,[4] and Yong-Guan Zhu[3,5,*]

*Correspondence: hfqian@usx.edu.cn (H.Q.); ygzhu@rcees.ac.cn (Y.-G.Z.)

Received: June 28, 2025; Accepted: February 26, 2026; Published Online: March 3, 2026; https://doi.org/10.1016/j.xinn.2026.101338

© 2026 The Author(s). Published by Elsevier Inc. on behalf of Youth Innovation Co., Ltd. This is an open access article under the CC BY license (http://creativecommons.org/licenses/by/4.0/).

GRAPHICAL ABSTRACT

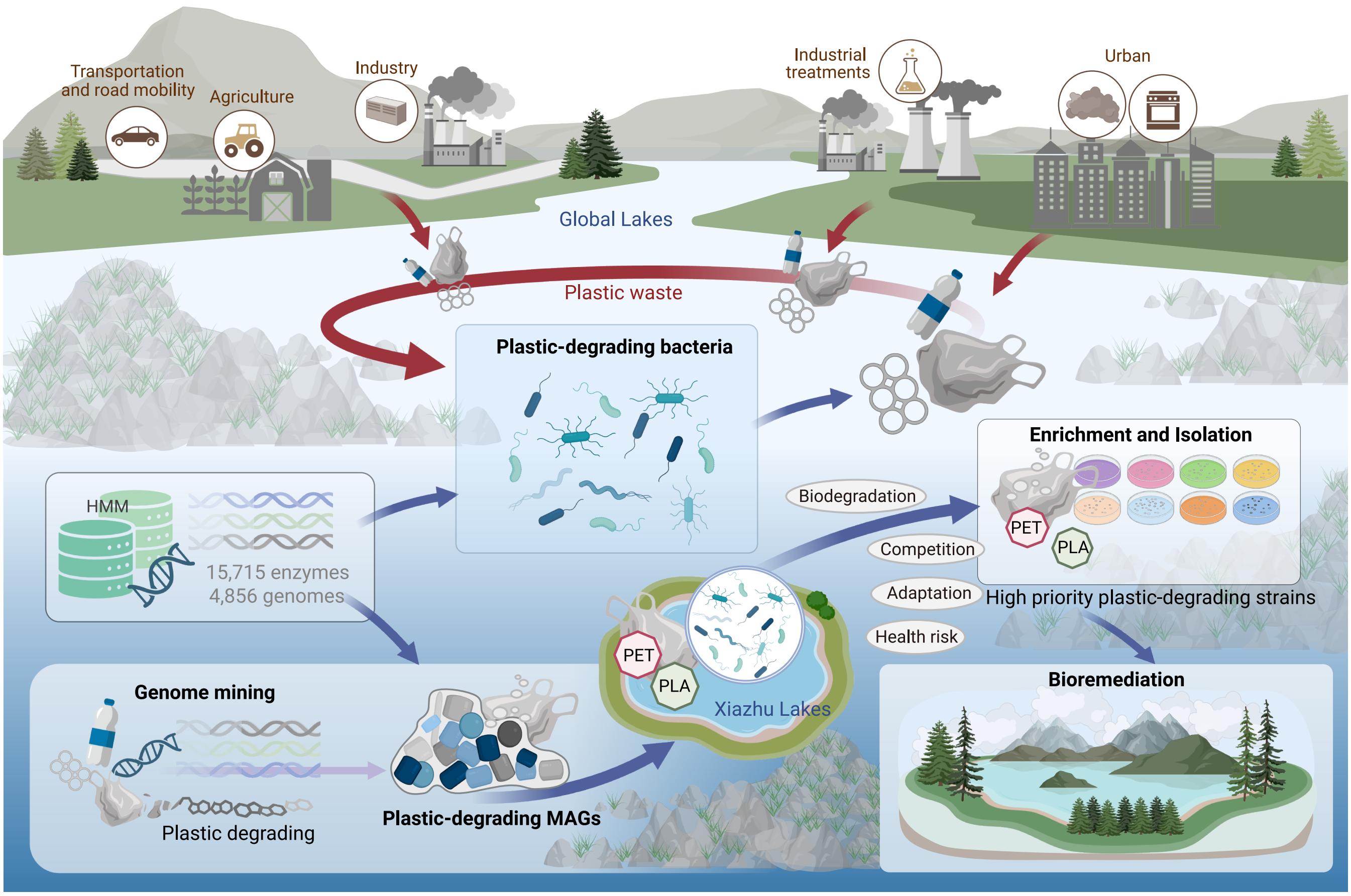

PUBLIC SUMMARY

- Plastic waste contributes to the geographical distribution of global lake plastic-degrading potential.
- We develop a computational framework to assess environmental application potential of plastic degraders.
- Most plastic-degrading bacteria lack complete essential growth factor pathways.
- We provide a proof-of-concept framework to identify and harness novel plastic degraders.

OPEN ACCESS

Environment & Ecology ARTICLE

Potential plastic biodegradation in lakes worldwide

Qi Zhang,[1,7] Zhenyan Zhang,[1,7] Ziyao Zhang,[2] Guoyan Qin,[2] Mingkang Jin,[5] Bingfeng Chen,[2] Yitian Yu,[3] Tingzhang Wang,[6] Meixia Wang,[6] Tao Lu,[2] Dong Zhu,[3] Li Cui,[3] Haifeng Qian,[1,*] Matthias C. Rillig,[4] and Yong-Guan Zhu[3,5,*]

[1]Institute for Advanced Study, Shaoxing University, Shaoxing 312000, China

[2]College of Environment, Zhejiang University of Technology, Hangzhou 310032, China

[3]Key Laboratory of Urban Environment and Health, Institute of Urban Environment, Chinese Academy of Sciences, Xiamen 361021, China

[4]Institute of Biology, Freie Universität Berlin, Berlin, Germany

[5]State Key Laboratory of Urban and Regional Ecology, Research Center for Eco-environmental Sciences, Chinese Academy of Sciences, Beijing 100085, China

[6]Key Laboratory of Microbial Technology and Bioinformatics of Zhejiang Province, Hangzhou 310012, China

[7]These authors contributed equally

*Correspondence: hfqian@usx.edu.cn (H.Q.); ygzhu@rcees.ac.cn (Y.-G.Z.)

Received: June 28, 2025; Accepted: February 26, 2026; Published Online: March 3, 2026; https://doi.org/10.1016/j.xinn.2026.101338

© 2026 The Author(s). Published by Elsevier Inc. on behalf of Youth Innovation Co., Ltd. This is an open access article under the CC BY license (http://creativecommons.org/licenses/by/4.0/).

Citation: Zhang Q., Zhang Z., Zhang Z., et al., (2026). Potential plastic biodegradation in lakes worldwide. The Innovation **7(8)**, 101338.

Plastic pollution is ubiquitous, yet the biodegradation of plastic waste remains poorly understood due to limited knowledge of microbial plastic degradation potential. Here, we demonstrate that plastic waste shapes the global distribution of plastic-degrading potential across 182,661 lakes worldwide using integrated metagenomic and machine learning analyses and identify a tipping point ($\geq$7.44 particles/m^3) for effective *in situ* bioremediation. We constructed, for the first time, a catalog of candidate plastic-degrading bacteria, including 15,715 nonredundant enzyme homologs and 4,856 metagenome-assembled genomes. To facilitate future applications, we developed a computational approach to categorizing candidate plastic-degrading bacteria according to their degradation potential, ecological risk, environmental adaptation, and competition capacity. Furthermore, we customized eight template culture media based on the growth factor biosynthesis profiles of high-priority candidate plastic-degrading bacteria. Using these media, we successfully enriched the plastic-degrading microbial communities and isolated a high-priority strain, *Serratia ficaria* HfyG-1, from Xiazhu Lake, which harbors a wide variety of previously uncharacterized putative degrading enzymes that effectively degrade polylactic acid and polyethylene terephthalate. Our study provides a molecular resource for the bioremediation of plastic-polluted environments worldwide and highlights a proof-of-concept framework for identifying, investigating, and exploiting unknown functional microorganisms for practical applications.

INTRODUCTION

Plastic pollutants have emerged as one of the greatest challenges of our time.[1,2] While most attention has focused on the ocean as a terminal aquatic sink,[3] freshwater ecosystems have been comparatively neglected. A recent standardized survey across 38 lakes revealed that plastic concentrations in some lakes can exceed those in oceanic gyres.[4] Owing to their limited water exchange and low oxygen levels, lakes have reduced self-purifying capacity and therefore accumulate various pollutants that are adsorbed on the surface of plastic debris.[5] Lakes also provide more direct exposure pathways for plastic debris to humans than many other ecosystems do because lakes are, for example, used for irrigation, drinking water, or recreation.[6] A growing body of evidence indicates that plastic contamination profoundly alters microbial community composition and ecological functions in freshwater systems.[7–10] Such continuous restructuring of lake microbiomes leads to changes in nutrient cycling, biofilm dynamics, and pathogen transmission, thereby threatening ecosystem stability and human health. Notably, persistent plastic contamination drives a bidirectional interaction between pollutants and the microbiota: while plastics reshape the microbial community structure, microorganisms adapt by evolving and enriching plastic-degrading traits. However, the true breadth and efficiency of microbial plastic degradation in lakes remain poorly understood.

Microbial degradation delivers dual environmental benefits: mitigating pervasive microplastic pollution while catalyzing a circular economy through waste upcycling.[11] Environmental microorganisms, which are chronically exposed to plastic pollution, have evolved unique enzymatic degradation systems that represent promising targets for enzyme discovery. Yoshida et al. first reported *Ideonella sakaiensis*—a polyethylene terephthalate (PET)-degrading bacterium—and its secreted enzyme PETase, which depolymerizes PET at 30°C, unveiling the metabolic potential of environmental microbes toward plastics.[12] Subsequent efforts have established curated repositories of validated plastic-degrading enzymes, including the PAZy[13] and PlasticsDB.[14] Nevertheless, cultivation-dependent approaches create a bottleneck for obtaining reliable insights into plastic degraders, including plastic-degrading microorganisms and their associated enzymes, thereby limiting the discovery of novel functional elements.[15] Meanwhile, analyses focused on individual locations limits our knowledge of the distribution of plastic degraders across lakes worldwide, as they vary as a function of various environmental and anthropogenetic factors.[15] Moreover, because of the wide variety of forms and types of plastics in lakes, the identification and screening of specific plastic degraders is particularly challenging.[15] Although recent studies have attempted to predict candidate plastic degraders on the basis of metagenomes from different habitats, their accuracy is limited by sequencing noise, coverage depth, and fragment length.[16,17] Genome-resolved metagenomics can be used to construct composite genomes from microbial populations,[18] providing reference genomes for uncultivated taxa and revealing enormous amounts of microbial dark matter.[19] Therefore, it is essential to construct a catalog that represents plastic-degrading bacteria and their related enzymes in lakes worldwide.

The central challenge confronting the environmental deployment of plastic-degrading bacteria resides in their limited capacity for sustained surface colonization and persistence on nutrient-depauperate plastic substrates.[20] Determining the environmental adaptability and competitiveness of plastic-degrading bacteria on a large scale is extremely challenging. Moreover, the ecological risk of the addition of plastic-degrading bacteria to the environment cannot be ignored; otherwise, unintended consequences may arise. Numerous studies have shown that plastic-degrading bacteria are potential pathogens or harbor ecological risk factors such as antibiotic resistance genes and virulence factors.[21–23] Some known plastic-degrading bacteria are potential pathogens, such as *Pseudomonas aeruginosa*,[24] *Klebsiella pneumoniae*,[25] and *Burkholderia cepacian*.[26] However, this issue has not yet received sufficient attention.

In this study, we systematically investigated the degradation potential, distribution, ecological risks, and metabolic functions of candidate plastic-degrading bacteria across lakes worldwide. We first applied hidden Markov models (HMMs) and machine learning to delineate the distribution and key environmental factors of plastic degradation potential in 182,661 lakes worldwide. Next, we constructed a metagenome-assembled genome (MAG) catalog of microbes with plastic-degrading potential in lakes worldwide and resolved their phylogenetic relationships. In addition, we developed a computational framework to quantitatively categorize candidate microbes according to their capacity for biodegradation, health risk, environmental adaptation, and resource competition. Additionally, we constructed biosynthetic profiles of essential growth factors in candidate plastic-degrading bacteria and designed customized culture media specifically to enrich these microorganisms. On the basis of these media, we enriched and isolated plastic-degrading bacterial strains from Xiazhu Lake (Huzhou, China) and rigorously verified their degradation abilities. Overall, we established a general workflow for identifying, investigating, and exploiting novel

candidate plastic-degrading bacteria for the biodegradation and bioremediation of plastic waste in lakes worldwide.

MATERIALS AND METHODS

Detailed descriptions of datasets, computational pipelines, experimental procedures, and statistical analyses are provided in the methods in the supplemental information.

Metagenomic datasets

Public freshwater lake metagenomes were retrieved from the European Nucleotide Archive and National Center for Biotechnology Information (NCBI) Sequence Read Archive (metadata as of January 16, 2023) using the keywords "Lake," "Metagenome," and "Freshwater." To minimize sampling bias, we excluded pond samples, non-water-column samples (e.g., sediments or aquatic organisms), lakes adjacent to point-source pollution or extreme environments, samples with inaccurate coordinates, and datasets lacking paired-end FASTQ reads or with mean read lengths below 100 bp. After filtering, 715 metagenomes from 95 lakes across 5 continents and 19 countries were retained.

Metagenomic assemblies and reconstruction of lake plastic-degrading MAGs

Metagenomic assemblies were generated using MEGAHIT within the MetaWRAP pipeline.[27] Reads were mapped to contigs with Bowtie2,[28] and resulting alignments were processed using SAMtools.[29] MAGs were reconstructed using a consensus binning strategy combining CONCOCT,[30] MaxBin2,[31] and MetaBAT2.[32] MAG quality was evaluated with CheckM,[33] retaining bins with ≥50% completeness and <10% contamination. To reduce functional false positives, downstream analyses were restricted to contigs longer than 10 kb and with taxonomic assignments consistent with their parent MAGs. Dereplication at 95% average nucleotide identity yielded 4,856 lake plastic-degrading MAGs (LPDMAGs), clustered into 1,370 species-level bins.

Enzyme dataset and construction of HMMs

A curated reference set of 184 experimentally validated plastic-degrading enzymes spanning 10 plastic types was compiled from published literature and databases.[13,14] These enzymes were grouped by polymer class and used to construct HMMs.[34] To expand sequence diversity, homologous sequences were retrieved from the UniProt TrEMBL database, dereplicated, aligned, and used to build 413 HMM profiles. Predicted proteins from all metagenomes were scanned using hmmsearch, retaining only the best hit per gene. Candidate hits were further validated using the machine learning-based CLEAN framework to predict enzyme commission numbers, ensuring functional consistency with known plastic-degrading activities.[35] This combined HMM-machine learning pipeline identified 45,154 nonredundant enzyme homologs across all lake metagenomes.

Functional annotation of LPDMAGs

Antibiotic resistance genes were annotated using the Comprehensive Antibiotic Resistance Database with RGI. Virulence- and pathogenicity-related genes were identified using the PHI-base (www.phi-base.org/) and Virulence Factor Database of Pathogenic Bacteria databases (mgc.ac.cn). To characterize ecological functionality, LPDMAGs were annotated for carbon-, nitrogen-, phosphorus-, and sulfur-cycling potential using a curated integration of NCycDB, SCycDB, QMEC, and GeoChip databases.[36–39] These annotations provided the basis for assessing metabolic versatility, ecological adaptation, and potential risk.

Reconstruction of growth factor biosynthesis pathways

To evaluate nutritional independence, biosynthesis pathways for 41 essential growth factors were manually reconstructed, encompassing 73 pathways and 469 Kyoto Encyclopedia of Genes and Genomes orthologs. Nearly complete LPDMAGs were annotated against the Kyoto Encyclopedia of Genes and Genomes database to determine pathway completeness, accounting for alternative routes and required intermediates. This analysis quantified the extent to which candidate plastic degraders rely on external nutrient supplementation.

Priority index for candidate plastic-degrading bacteria

We developed a computational framework to rank candidate plastic-degrading bacteria by integrating plastic-degrading potential, ecological risk, environmental adaptation capacity, and competitive capacity. Plastic-degrading potential was defined as the abundance of enzyme homologs per MAG. Adaptation and competitive capacities were estimated from functional traits related to stress tolerance, biofilm formation,[40] and elemental cycling. Ecological risk was quantified based on pathogenicity, virulence, and antibiotic resistance profiles.[41,42] These components were combined into a priority index, emphasizing high degradation potential and minimal ecological risk. Candidates were classified into four priority tiers using unsupervised clustering.

Enrichment, isolation, and genomic characterization

Plastic colonization experiments were conducted in Xiazhu Lake (Huzhou, China) using polylactic acid (PLA) and PET substrates for *in situ* enrichment. Plastisphere communities were collected and transferred into mineral salt media supplemented with tailored growth factors. After long-term incubation, bacterial isolates were obtained through serial dilution and plating. A representative high-priority isolate, *Serratia ficaria* HfyG-1, was subjected to whole-genome sequencing, assembly, and annotation following standard quality control and functional annotation pipelines.

Verification test of plastic degradation

Plastic degradation assays were performed using enriched communities and isolated strains incubated with PLA or PET as the sole carbon source. Degradation efficiency was assessed by mass loss measurements and surface characterization using scanning electron microscopy, atomic force microscopy, and Fourier transform infrared (FTIR) spectroscopy.

Statistical analysis and visualization

Statistical analyses were performed using nonparametric tests and multivariate ordination methods as described in the main text. Data visualization and phylogenetic analyses were conducted using standard R, Python, and graphical software tools.

RESULTS

Geographical distribution of microbial plastic-degrading potential in lakes

First, we compiled a catalog of 15,715 nonredundant candidate enzyme homologs with the potential to degrade 10 different plastics. We then characterized the distribution of microbial plastic-degrading potential on the basis of gene abundance across 715 metagenomes derived from 95 lakes (Figure S1; Table S1). Using HMMs integrated with machine learning, we identified a total of 45,154 putative plastic-degrading enzyme hits across all samples, spanning 10 representative polymer types (Table S2), and the pattern of plastic-degrading potential differed significantly across countries (Adonis analysis, R^2 = 0.21, p < 0.001; Figure S2A), especially among lakes (Adonis analysis, R^2 = 0.49, p < 0.001). Pairwise Adonis analysis based on Bray-Curtis dissimilarity revealed a different distribution of plastic-degrading potential among almost all the lakes (p < 0.001; Figure S2B), indicating a unique lake landscape with plastic-degrading potential.

The total plastic-degrading potential was higher in mid-latitude lakes (R^2 = 0.012, p < 0.05; Figure 1A), reflecting the combined influence of moderate climatic conditions and higher anthropogenic plastic inputs that favor microbial adaptation and enzymatic activity. The proportion of degrading potential for biodegradable, polyolefin, and conventional polyester plastics varied geographically (Figure 1B) due to differences in abundance of putative plastic-degrading enzyme-encoding genes across 95 lakes spanning 19 countries and 5 continents (Figures 1C, S3, and S4). We found that the microbial degradation potential varied substantially among the different plastic types across all the lake metagenomes (Figure S5). The genes encoding putative enzymes for PHA, PET, and polyurethane degradation were the most enriched, whereas those associated with polyolefin degradation showed the lowest abundance. Overall, the degradation potential of biodegradable and conventional polyester plastics was comparable and markedly higher than that of polyolefins, indicating that ester-linked polymers are more readily metabolized by lake microbial communities.

Plastic pollution contributes to plastic-degrading potential in lake microbiomes

To examine the geographical mechanisms that drive the spatial patterns of plastic-degrading potential in lake microbiomes, we integrated 37 environmental and anthropogenic predictors constraints to map the microbial plastic degradation potential of 182,661 lakes worldwide (>1 km^2) via machine learning (Figure 2A; Table S3). Among these predictors, national-level plastic waste generation and management statistics were used as proxies for contamination pressure, providing a large-scale environmental context rather than lake-specific pollution levels. The Random Forest model exhibited the best predictive

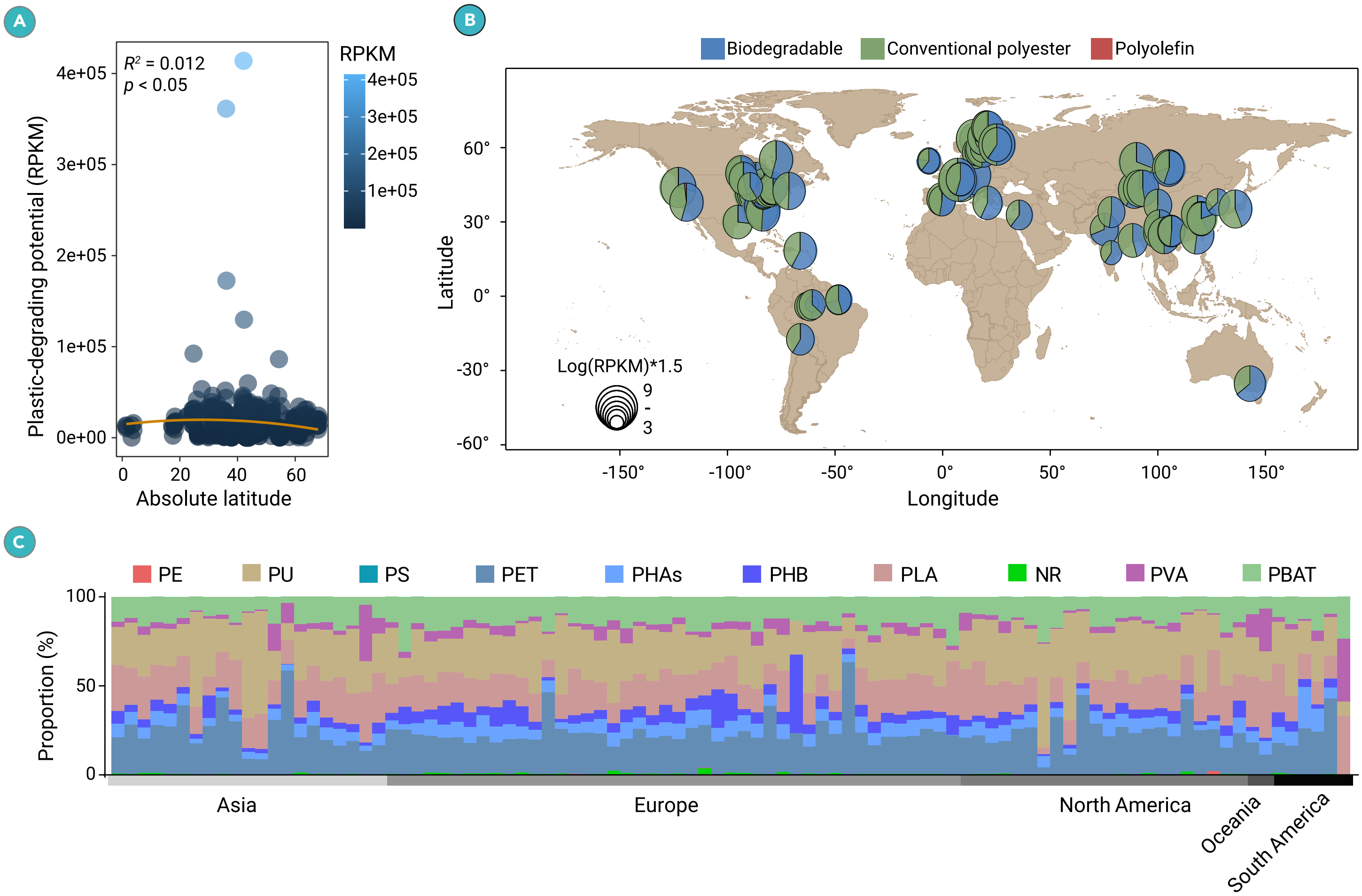

Figure 1. Composition and global distribution of microbial plastic-degrading potential in lakes (A) Total plastic-degrading potential is higher in mid-latitude lakes. The blue color gradient indicates the abundance of putative plastic-degrading enzyme-encoding genes. (B) Distribution of microbial plastic-degrading potential across 95 lakes. Blue, green, and red denote biodegradable, polyolefin, and conventional polyester plastics, respectively. Circle size represents the abundance of putative plastic-degrading enzyme-encoding genes [log(RPKM) × 1.5]. (C) Composition of putative plastic-degrading enzyme-encoding genes across 95 lakes spanning Asia, Europe, North America, Oceania, and South America, covering major plastic types, including PVA (polyvinyl alcohol), PHAs (polyhydroxyalkanoates), PHB (polyhydroxybutyrate), PU (polyurethane), PET (polyethylene terephthalate), PBAT (polybutylene adipate terephthalate), PS (polystyrene), PLA (polylactic acid), PE (polyethylene), and NR (natural rubber). RPKM, reads per kilobase per million mapped reads.

performance, achieving the highest accuracy (R^2 = 0.73) and the lowest root mean-square error (6605.01) among all the tested algorithms (Figure S6A), and residual analysis confirmed its robustness, with residuals tightly centered around zero (Figures S6B–S6D). In addition to geographic variation, plastic-related factors, especially national waste imports per capita, and climatic variables, such as moisture index seasonality and precipitation in the coldest quarter, were the strongest predictors of microbial plastic degradation potential (Figures 2B and S6E). These findings underscore the interplay between anthropogenic inputs and climate: plastic waste defines exposure intensity, whereas water balance and seasonality regulate microbial metabolism, jointly shaping the ecological niches of candidate plastic-degrading bacteria.

To further substantiate these model-derived patterns, empirical monitoring data of plastic abundance (particles/m^3) from 25 lakes spanning 21 countries and 5 continents were obtained following the standardized protocol of Nava et al.[4] A significant positive correlation (Figure 2C; generalized additive model: R^2 = 0.55, $p < 0.05$; partial least squares: R^2 = 0.19, $p < 0.05$) was observed between plastic abundance and microbial plastic degradation potential. The generalized additive model revealed a nonlinear tipping point at 7.44 particles/m^3: below this threshold, the degradation potential increased sharply with increasing plastic load, whereas beyond this threshold the potential plateaued despite increasing pollution levels. This pattern suggests that excessive contamination, especially under unfavorable climatic regimes, may drive ecosystems toward functional instability and reduce microbial resilience.

Collectively, these findings demonstrate that plastic waste and climatic conditions interactively govern microbial degradation capacity in lake ecosystems. Recognizing this coupling provides critical insight for climate-adaptive management, emphasizing the need for targeted monitoring and stricter plastic control in regions where climatic variability limits microbial self-remediation potential while maintaining pollution below ecological tipping points to prevent irreversible functional disruptions.

LPDMAG catalog

To reconstruct previously unexplored genomes of candidate plastic-degrading bacteria, we performed a large-scale single-sample metagenomic assembly on all samples and retained only those datasets in which the taxonomic affiliation (at the family level) of any hits found in candidate plastic-degrading contigs was consistent with the overall taxonomy of the MAGs (Figure 3A). After identification and filtration, we generated a total of 4,856 MAGs with plastic-degrading potential that met or exceeded the minimum information about a metagenome-assembled genome medium-quality standard (completion ≥50%, contamination ≤5%),[43] with a mean completeness of 82.20% (±14.43%) and a mean contamination rate of 3.15% (±2.38%), which we refer to as the LPDMAG catalog. The N50 size of these MAGs ranged from 1,370 to 1,032,389 bp, and the GC content varied from 25% to 75% (Figure 3B; Table S4). Approximately 341 (7.02%) LPDMAGs were identified as high-quality genomes with completeness >90%, contamination <5%, the presence of 23S, 16S, and 5S rRNA genes and at least 18 tRNAs according to recent guidelines. Moreover, 1,744 (35.91%) of the LPDMAGs had completeness ≥90% and contamination ≤5% but lacked a complete set of rRNA genes or had fewer than 18 tRNAs, further suggesting that recovering a full complement of rRNA gene remains challenging for nearly complete MAGs.[43] In addition, LPDMAGs covered 83% of the collected samples spanning 86 lakes, indicating that this catalog is a substantial but incomplete fraction of the diversity of candidate plastic-degrading bacteria from lakes worldwide.

www.the-innovation.org

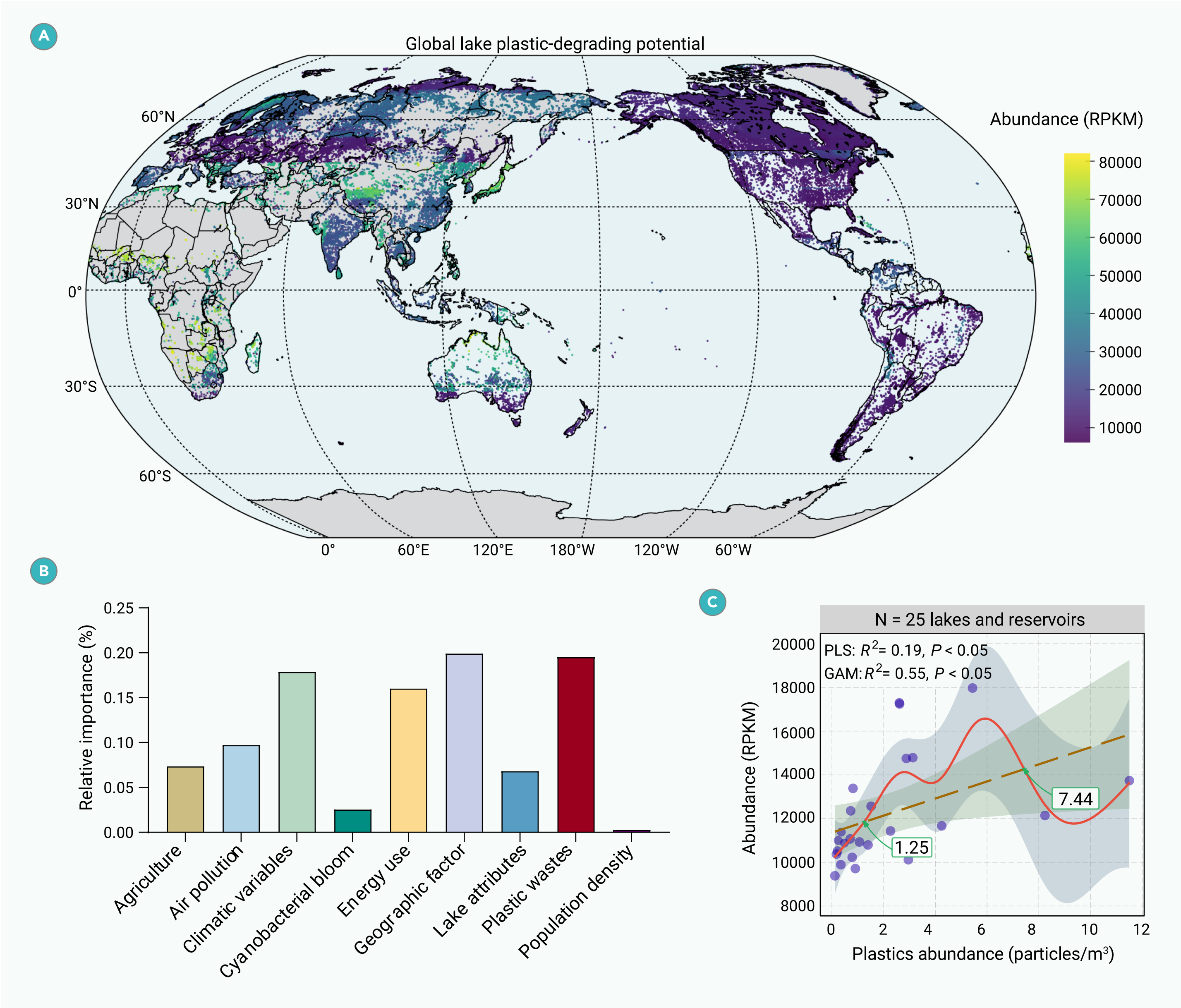

Figure 2. Global mapping of microbial plastic-degrading potential in lakes (A) Predicted plastic-degrading potential across 182,661 lakes worldwide (>1 km^2). The color gradient from yellow to purple indicates the abundance of putative plastic-degrading enzyme-encoding genes [log(RPKM)], from high to low. (B) Relative importance of geographical variables highlights plastic pollution as the dominant driver shaping microbial plastic-degrading potential. (C) Plastic abundance (particles m^{-3}) is positively associated with microbial plastic-degrading potential in lakes. A threshold (≥7.44 particles m^{-3}) indicates a tipping point beyond which ecological disruption is influenced by factors exceeding simple resource availability. RPKM, reads per kilobase per million mapped reads; GAM, generalized additive model; PLS, partial least squares.

MAG analyses identify 1,370 species-level candidate plastic-degrading bacteria in lakes

To explore the taxonomic components in the LPDMAG catalog, we clustered the 4,856 MAGs into 1,370 species-level genome bins (denoted as candidate plastic-degrading bacteria) on the basis of a 95% whole-genome average nucleotide identity threshold (Figure S7). These candidate degraders were taxonomically annotated into 21 bacterial phyla and 2 archaeal phyla on the basis of the Genome Taxonomy Database, which is widely used.[44] Next, we constructed a phylogenetic tree of all the LPDMAGs (Figure 3C), indicating that the diversity of candidate plastic-degrading bacteria across the Tree of Life was expanded by this catalog. By comparing 400,596 reference genomes, including 127,100 genomes from the RefSeq database (November 2022) and 273,496 genomes from GenBank (November 2022), we identified 609 unknown and 761 known species at the threshold of 95% average nucleotide identity. The proportion of singleton MAGs among unknown MAGs (73.9%) was substantially greater than that among known plastic-degrading bacteria (49.0%), indicating the critical contribution of the LPDMAG catalog to recovering rare species of lake microbiomes. Moreover, we compared these species against nearly 1,008 previously reported species-level plastic-degrading bacteria from PlasticDB (https://plasticdb.org/) and other literature sources (Table S5). In total, we identified 99.9% species-level, 99.3% genus-level, 79.9% family-level, 77.2% order-level, 78.1% class-level, and 69.6% phylum-level novel candidate plastic-degrading bacteria within the LPDMAG catalog (Table S6), substantially expanding the molecular resources of candidate plastic-degrading bacteria.

Most candidate degraders exhibited activity toward multiple plastic types, indicating substantial functional overlap rather than strict substrate separation (Figure S8). For instance, in the PLA-contaminated sites, 93% of the identified candidate degraders encoded enzymes targeting not only PLA but also other polymers, and their relative abundance was significantly higher than that observed in uncontaminated sites (Figure S9). These patterns suggest that degradation preferences are primarily governed by the enzyme repertoire rather than by taxonomic boundaries. Nonetheless, a few lineages showed narrow specialization, such as *Dermatophilaceae*, which appeared to be restricted to polyhydroxyalkanoates, and *Streptococcaceae*, which is restricted

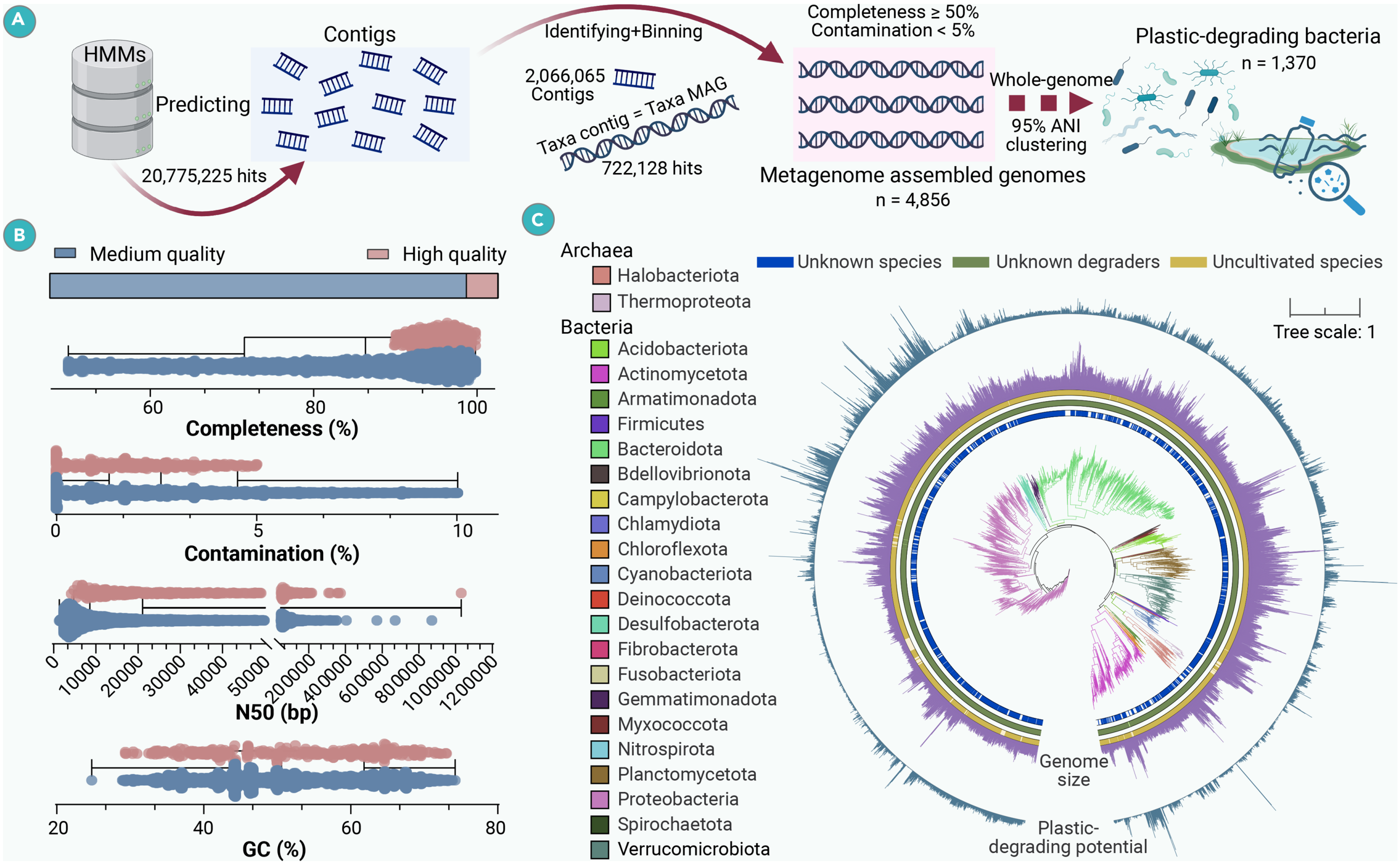

Figure 3. Identification of candidate plastic-degrading bacteria across global lakes (A) Overview of the computational framework developed to assign putative plastic-degrading enzyme-encoding genes to their microbial hosts based on MAGs. (B) Distribution of quality metrics across 4,856 MAGs; blue and red denote medium- and high-quality MAGs, respectively. (C) Phylogenetic tree of 4,856 candidate plastic-degrading MAGs recovered from lake ecosystems worldwide. HMMs, hidden Markov models; MAGs, metagenome-assembled genomes.

to polyurethane. Overall, these results reveal that functional convergence and enzymatic versatility are widespread among candidate plastic-degrading microbes, with specialization confined to a limited subset of taxa.

Across all the metagenomes, an average of 567 candidate plastic-degrading taxa per sample was identified, with a mean abundance of 813 reads per kilobase per million mapped reads (Figure S10). Although degrader diversity remained relatively consistent among the lakes, their abundance exhibited pronounced spatial heterogeneity, with several sites showing marked enrichment. These patterns suggest that local environmental conditions and contamination pressure jointly shape the biogeography of candidate plastic-degrading microorganisms in freshwater ecosystems. To further examine ecological interactions, we constructed a co-occurrence network based on abundance correlations between LDPMAGs and non-degrading taxa (Figure S11). The network comprised four major modules (modules 2, 3, 4, and 30) dominated by non-degraders such as *Comamonas*, *Magnetospirillum*, *Polynucleobacter*, and *Granulicella*, which were strongly associated with candidate plastic degraders. These associations indicate potential metabolic complementarity and ecological cooperation, whereby non-degraders may contribute to nutrient cycling or biofilm stabilization, supporting the persistence and activity of candidate degraders under varying lake conditions.

Hot zones of candidate plastic-degrading bacteria in lakes

To identify the ecological hot zones of candidate plastic-degrading bacteria, we analyzed metagenomic data from a plateau karst lake encompassing lake water, plastisphere, and sediment samples with quantified plastic debris concentrations.[10] The plastic debris concentration ranged from 20.27 to 58.80 n L^{-1} in the lake water and 583.33 to 996.67 n kg^{-1} in the sediments, indicating measurable plastic pollution across all sampling sites. The plastisphere harbored the highest microbial richness and diversity, significantly exceeding those of the lake water and sediments (Figure S12). Moreover, lakes with relatively high plastic pollution levels presented correspondingly high abundances of candidate plastic-degrading bacteria in sediments, suggesting that contamination intensity promotes the enrichment of functional degraders. These findings reveal that both the plastisphere and lake sediments act as key ecological reservoirs (hot zones) for candidate plastic-degrading microorganisms. The plastisphere provides a selective and nutrient-rich interface that facilitates microbial colonization and metabolic cooperation, whereas sediments serve as long-term sinks where plastic-associated degraders accumulate and persist.

Promising candidate plastic-degrading bacteria for environmental application

To better understand the functional characteristics of these candidate plastic-degrading bacteria, we predicted full-length putative protein sequences from the 1,744 high-completeness MAGs (>90% completeness, <5% contamination) of the LDPMAG catalog. We developed a novel computational framework to assess the environmental applicability of these candidate degraders in the environment, compiling their four functional characteristics: plastic-degrading potential, ecological risk, adaptation capacity, and competitive capacity (Figure 4A). In addition to their plastic-degrading potential, the key step is that candidate degraders can survive on plastic surfaces under nutrient limitation and exogenous stress.[22] Biofilm formation potential represents the capacity of candidate plastic-degrading bacteria to adapt to a new environment. Next, the potential for recycling core nutrient elements (carbon, nitrogen, phosphorus, and sulfur) was used to assess the competitive capacity of candidate degraders in resource-poor environments. In addition, we assessed the ecological health risk of these candidate degraders on the basis of commonly used risk factors,[42,45] antibiotic resistance genes (with high human health risk), pathogenic factors (hypervirulence-related genes), and virulence factors. Overall, we calculated the priority index of the candidate plastic-degrading bacteria (for details, see materials and methods). Using the K-means algorithm, a well-known and widely used clustering method,[46] we divided the candidate degraders into

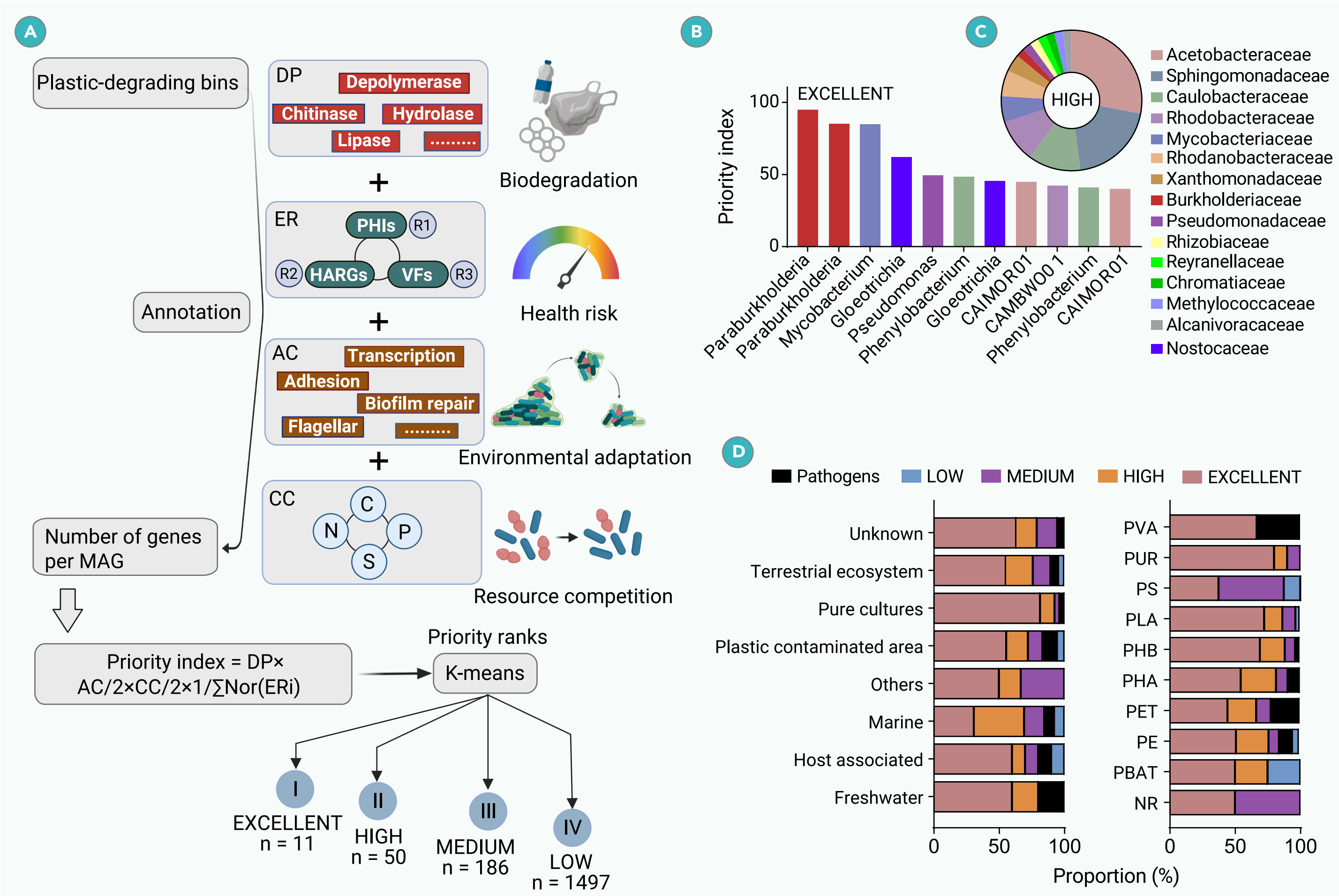

Figure 4. Priority index of candidate plastic-degrading bacteria (A) Framework for calculating the priority index of 1,744 candidate plastic-degrading MAGs with near-complete genomes, based on four factors: plastic-degrading potential (DP), ecological risk (ER), adaptation capacity (AC), and competition capacity (CC) (see materials and methods for details). (B) Priority index and taxonomic composition of candidates classified as EXCELLENT degraders. (C) Taxonomic proportions of candidates classified as HIGH degraders. (D) Priority index of experimentally validated plastic-degrading strains. MAGs, metagenome-assembled genomes; PHI, pathogen-host interaction (hypervirulence); HARGs, high-risk antibiotic resistance genes; VFs, virulence factors; C, carbon; N, nitrogen; P, phosphorus; S, sulfur.

EXCELLENT (n = 11), HIGH (n = 50), MEDIUM (n = 186), and LOW (n = 1,068) categories. The most important EXCELLENT candidate degrader, *Paraburkholderia*, showed strong environmental application potential, with the highest degradation potential, adaptation, and competition capacity, and the lowest risk (Figure 4B). *Acetobacteraceae*, *Sphingomonadaceae*, and *Caulobacteraceae* were the main HIGH-priority candidate plastic-degrading bacteria (Figure 4C). In addition, the priority score of candidate plastic-degrading bacteria varied among different plastic types; for example, *Acetobacteraceae* was a higher priority when applied to polyethylene glycol, polyurethane, and phthalic acid pollution than when applied to other types of pollution (Figure S13). Therefore, in practical applications of this framework, the type of polluting plastic should be explicitly identified and considered.

To illustrate the applicability and necessity of this priority assessment framework, we calculated the priority indices of 165 experimentally validated plastic-degrading strains (with available species-level reference genomes) (Table S7). These strains were mainly isolated from pure cultures, terrestrial water, and plastic-contaminated water, whereas a few strains were isolated from freshwater. As shown in Figure 4D, most plastic-degrading strains fall into the EXCELLENT and HIGH categories, although a small fraction is clustered in the LOW and pathogen categories. Importantly, degraders of polyester-based plastics such as PLA, PET, and polybutylene adipate terephthalate were classified into the EXCELLENT and HIGH categories, reflecting their relatively well-characterized enzymatic pathways and higher biodegradation potential. In contrast, degraders of more recalcitrant plastics, such as polyethylene, and polystyrene, were more often assigned to the LOW or MEDIUM categories, indicating limited efficiency under current conditions.

Enrichment strategies for high-priority candidate plastic-degrading bacteria

To guide future enrichment, isolation, and culture, we manually restructured a total of 73 biosynthetic pathways with 299 proteins and 469 KO numbers, covering 41 essential growth factors (e.g., vitamins, amino acids, nucleotides, and coenzymes) for bacteria or archaea, on the basis of the Kyoto Encyclopedia of Genes and Genomes database (Table S8). As expected, many EXCELLENT and HIGH candidate plastic-degrading bacteria lacked complete biosynthetic pathways for some of these essential nutrients (Figure 5). The biosynthesis profiles of essential growth factors corresponding to each candidate degrader were not unique; thus, we formulated eight template media according to the metabolic profile of high-priority degrading bacteria to enrich and isolate more previously unknown degrading bacteria (Table S9).

Genomic analysis revealed that the EXCELLENT candidate plastic-degrading bacteria exhibit high degradation potential for both PLA and PET, representative biodegradable and non-biodegradable plastics, respectively, which dominate global plastic production volumes.[47] We therefore collected PLA and PET fragments after 6 months of *in situ* enrichment in Xiazhu Lake (30.5156°E, 120.0483°N) to obtain ecologically primed plastisphere samples. These were subsequently inoculated into eight chemically defined media containing PLA or PET as the sole carbon source (Figure 6A). After 60 days, the type 4 cultured bacterial community, which was most significantly enriched in both PLA and PET groups, was used to verify the degradation ability of plastics. Changes in surface morphology and weight loss serve as key indicators of plastic degradation. Notably, significant weight reductions were observed in PLA and PET groups after inoculating the type 4 cultured bacterial community for 90 days, which presented weight losses of 37.1% and 33.1%, respectively (Figure 6B).

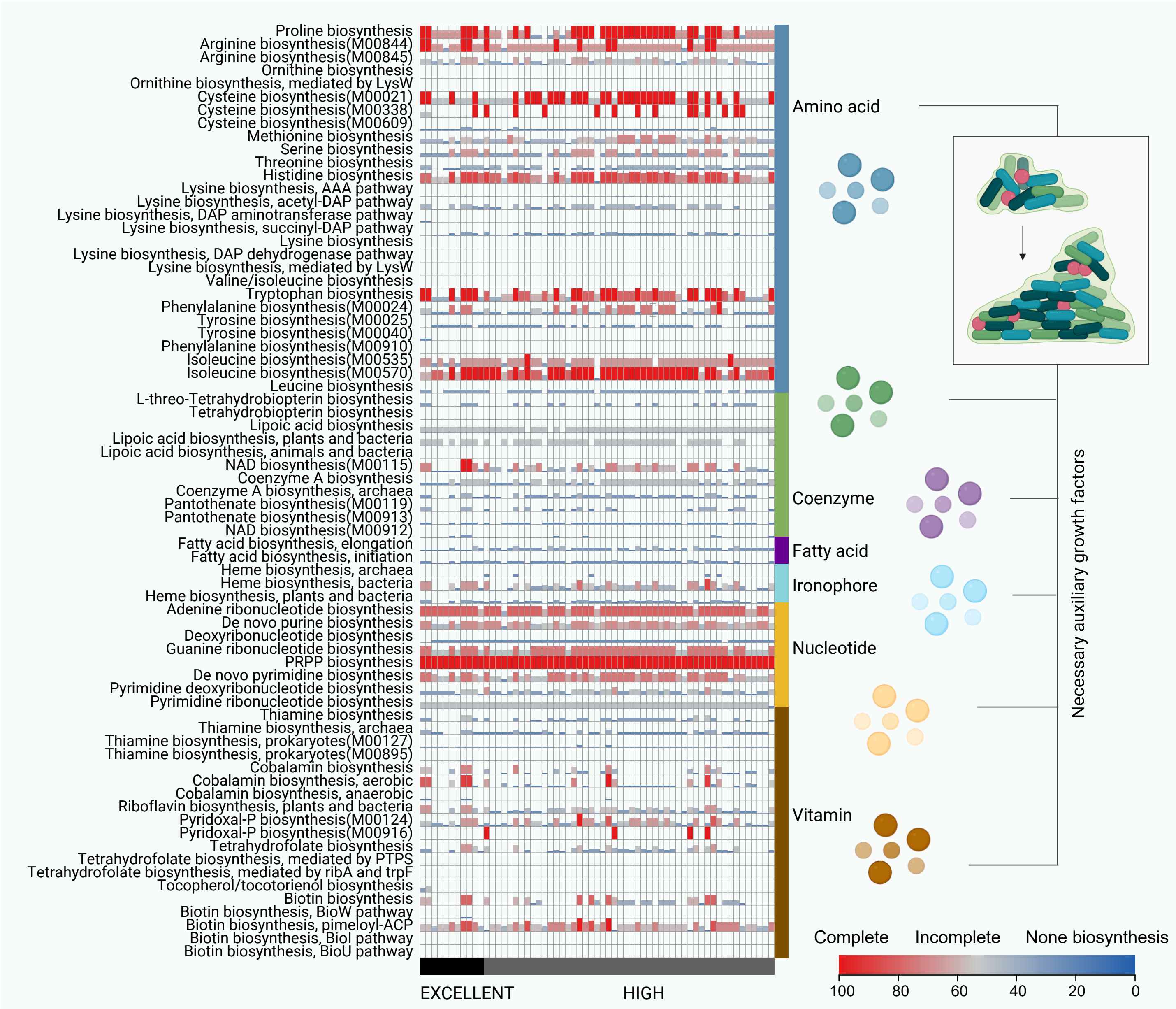

Figure 5. Biosynthetic profiles of essential growth factors in high-priority candidate plastic-degrading bacteria Heatmap showing the completeness of biosynthetic pathways for essential growth factors across EXCELLENT and HIGH-priority candidate plastic-degrading bacteria. Pathways are grouped into major functional categories, including amino acids, coenzymes, fatty acids, ironophores, nucleotides, and vitamins. Each row represents a specific biosynthetic pathway, and each column represents an individual candidate degrader. Color intensity indicates pathway completeness, with red denoting complete pathways, light colors indicating partial pathways, and blue indicating the absence of biosynthesis. The schematic on the right illustrates the conceptual framework linking growth factor auxotrophy to microbial community assembly and enrichment under nutrient-limited conditions, highlighting the role of necessary auxiliary growth factors in supporting the colonization and persistence of plastic-degrading bacteria.

Scanning electron microscopy analysis revealed the development of distinct surface features, such as pits, gullies, folds, and grooves, along with localized detachment on the surfaces of PLA and PET following bacterial treatment (Figure 6C). Meanwhile, atomic force microscopy revealed substantial increases in surface roughness in the treatment groups, with PLA and PET showing 15.6- and 6.2-fold increases, respectively, compared with those of the control group (Figures 6D and S14). Furthermore, the FTIR spectra of these plastics clearly revealed that the chemical bonds within the PLA and PET were broken down (Figure 6E). ple, FTIR analysis of PLA showed the shifts and intensity changes in the C=O stretch (1,735–1,750 cm^{-1}) and C-O stretch (1,000–1,300 cm^{-1}), indicating the hydrolysis of PLA.

Excellent plastic-degrading strain *S. ficaria* HfyG-1 isolated from lakes

We obtained 22 and 31 independent bacterial colonies from type 4 communities incubated with PLA and PET particles, respectively, under conditions where plastics served as the sole carbon source (Figure S15). The dominant colonies with the most robust growth and degradation activity were selected for whole-genome sequencing, revealing a shared *S. ficaria* strain (HfyG-1). By evaluating four priority indices—plastic-degrading potential, ecological risk, adaptation capacity, and competition capacity—we identified *S. ficaria* HfyG-1 as an EXCELLENT plastic-degrading strain, particularly noted for its potential to degrade both PLA and PET (Figure 7A). Moreover, genomic interrogation identified 10 putative plastic-degrading enzymes, including esterases, PETases, and specialized PLA depolymerases, which shared generally low sequence identities with reported plastic-degrading enzymes, with an average identity of <30% (15%–39%). However, structural comparisons showed high overlap, with mean template modeling scores >0.8, coverage >0.9, and root mean-square deviation <3.0, indicating that the putative enzymes were structurally conserved but sequence divergent, strongly supporting their novelty (Figure 7B). Furthermore, metatranscriptomic surveys from six lakes across five countries showed that these putative enzymes were

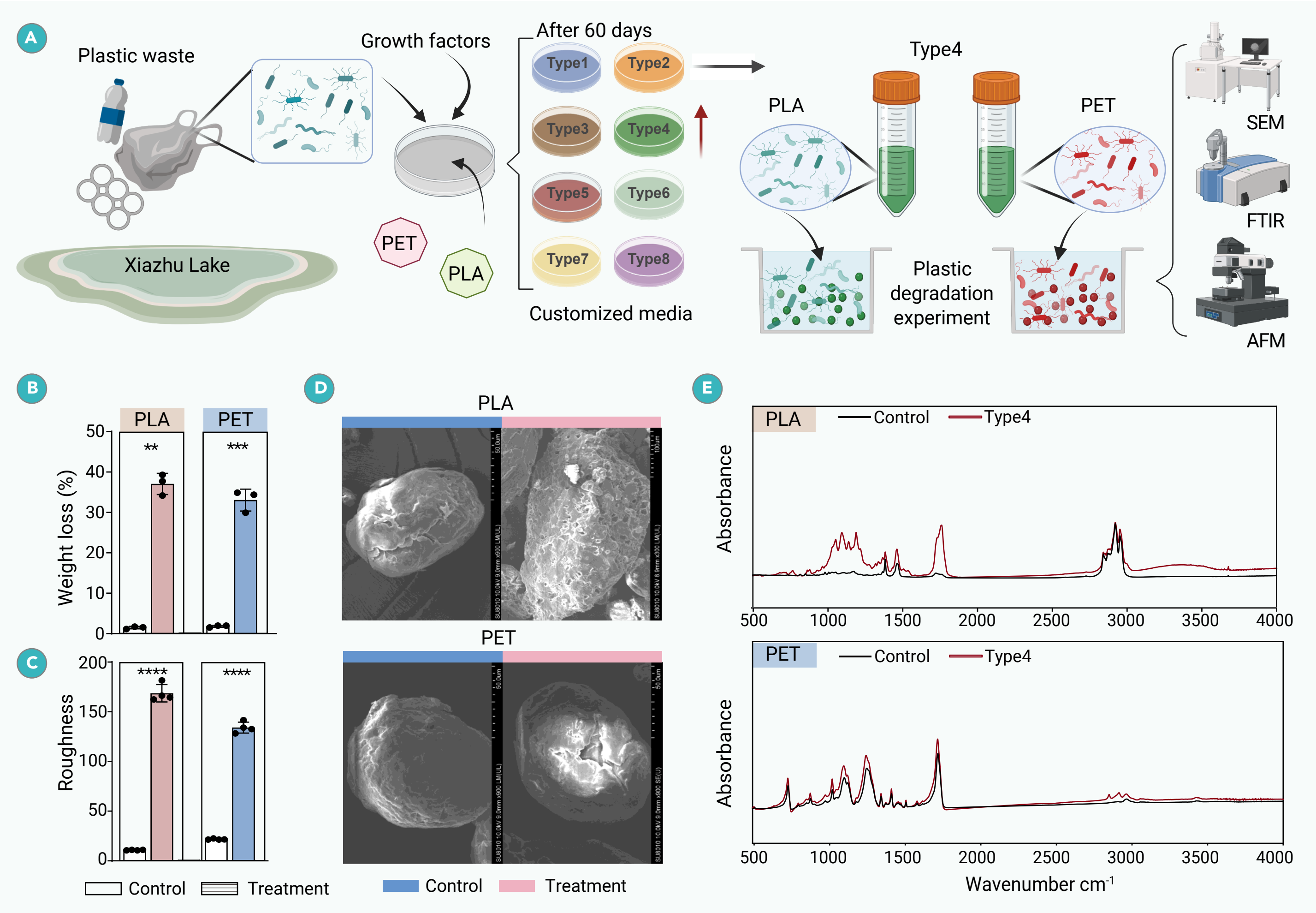

Figure 6. Enrichment and experimental validation of potential plastic-degrading bacterial communities (A) Microbial communities associated with plastic waste from Xiazhu Lake were enriched in customized media containing PLA or PET as the sole carbon source. (B) Weight loss of PLA and PET plastics after incubation with enriched microbial communities, showing significantly higher degradation in treatment groups compared with controls. Data are presented as mean ± SEM. (C) Surface morphology of PLA and PET after microbial treatment, revealing clear signs of microbial degradation. Scale bars, 50 μm. (D) Quantification of surface roughness of PLA and PET plastics following microbial treatment. (E) Fourier transform infrared spectra of PLA and PET plastics before treatment (black curves, control) and after incubation with the type 4 enriched bacterial community (red curves, treatment).

frequently detected (Figure S16), suggesting that they are actively expressed in natural environments and likely to play functional roles in plastic degradation *in situ*.

To investigate potential synergistic effects among microbial communities during plastic degradation, we analyzed the full-length 16S rRNA datasets from the PLA and PET degradation microcosms at 30 and 60 days. The results showed that *S. ficaria* was significantly enriched overtime on both PLA and PET surfaces ($p < 0.05$; Figure S17A). Co-occurrence network analysis further revealed that *S. ficaria* extensively interacted with other bacterial taxa, such as *Glutamicibacter*, *Achromobacter*, *Ralstonia*, and *Lactobacillus*, forming stable consortia that may facilitate substrate breakdown and nutrient recycling (Figures S17B and S17C). These findings demonstrate that plastic degradation in the microcosm is likely driven by synergistic microbial networks rather than by single degraders acting independently.

To further validate the degradation capacity of *S. ficaria* HfyG-1, we inoculated this strain into type 4 medium with PLA and PET as the sole carbon source, and the weight loss rate was up to 35.4% and 36.4%, respectively, after 90 days (Figure 7D). Scanning electron microscopy also revealed that the surfaces of PLA and PET contained obvious pits, grooves, and folds after treatment with degrading strains (Figure 7E). FTIR analysis revealed that the chemical groups of PLA and PET were damaged to varying degrees after being inoculated with plastic-degrading strains for 90 days (Figure 7F). Collectively, these results demonstrate that the isolated strain *S. ficaria* HfyG-1 exhibits robust viability and high environmental application potential, with demonstrated capability to degrade both PLA and PET. This evidence validates the effectiveness of our framework for identifying plastic-degrading bacteria.

DISCUSSION

Given its generally persistent nature, plastic pollution is possibly a hallmark of the Anthropocene.[22] Lakes, as sentinels of environmental change,[48] are suffering from heavy plastic pollution, and microbes have evolved strategies to cope with this contamination. This study describes the microbial potential for degrading plastics, including the majority of massively produced and globally polluting polymers, and predicts their geographical distribution across 182,661 lakes worldwide via machine learning. We demonstrated that plastic content is strongly associated with the plastic-degrading potential of microbes in lakes globally, suggesting that plastic pollution drives the accumulation of plastic-degrading potential. Therefore, mining ecological resources for plastic degradation helps to further understand the current situation of plastic pollution in lakes and is conducive to the subsequent development of plastic biodegradation technology.[49]

To the best of our knowledge, this is the first report of a MAG catalog with plastic-degrading potential for lake ecosystems, comprising 4,856 genomes of 1,370 species from 715 metagenomes. This catalog represents a step change compared with currently available studies on candidate plastic-degrading bacteria identified via metagenomes, which are limited by short sequence fragments, taxonomic annotation bias, small sample sizes, and uneven sequencing depths.[17] Lakes host highly diverse and novel microbial populations, with 609 species-level genome bins

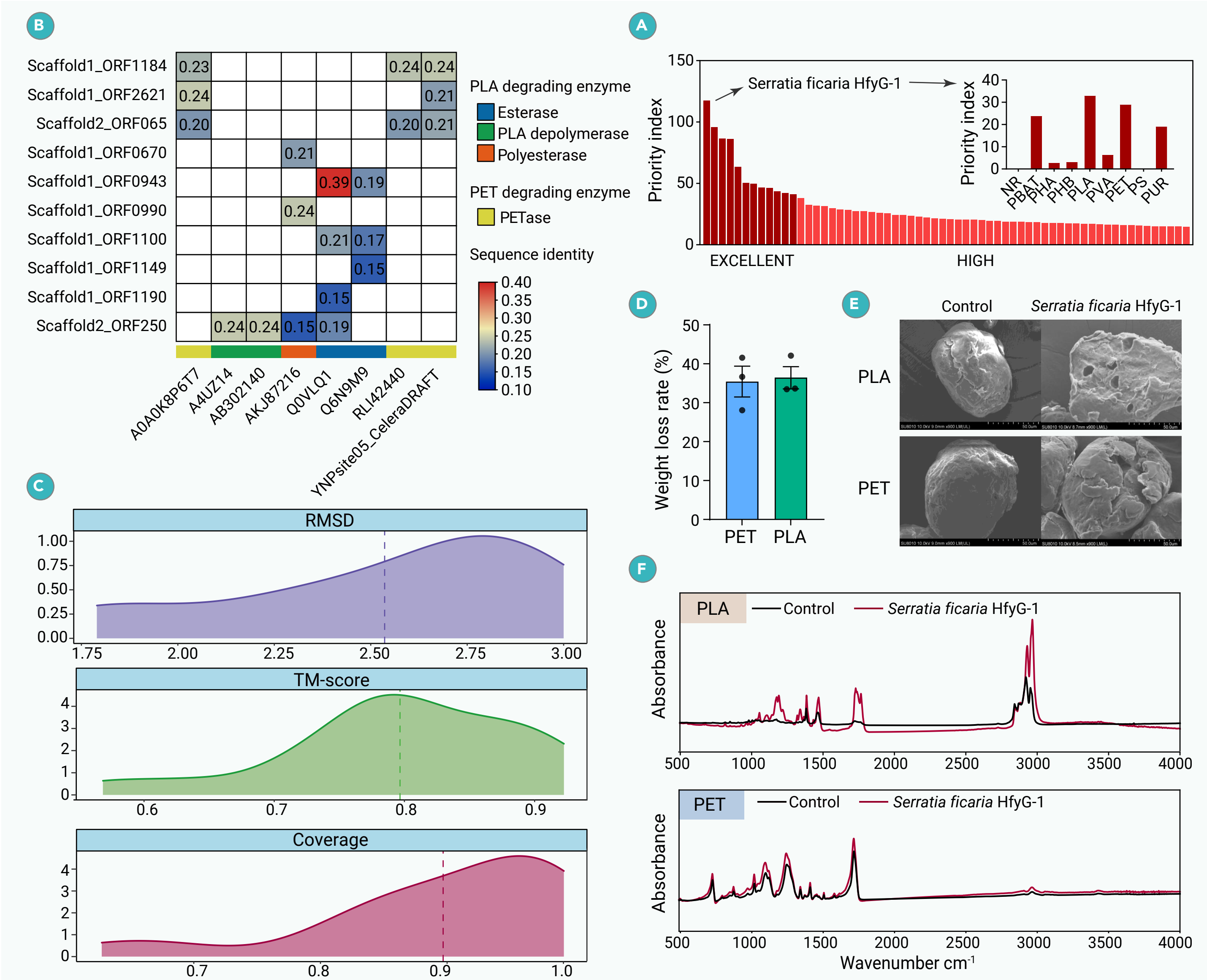

Figure 7. Genomic and experimental characterization of the plastic-degrading bacterium *S. ficaria* HfyG-1 (A) Identification of *S. ficaria* HfyG-1 as an EXCELLENT candidate plastic degrader, with strong predicted potential for polylactic acid (PLA) and polyethylene terephthalate (PET) degradation. (B) Sequence identity of 10 putative plastic-degrading enzymes encoded by *S. ficaria* HfyG-1 compared with reported reference enzymes. (C) Structural comparison between predicted enzymes and known plastic-degrading enzymes based on root mean-square deviation, TM score, and structural coverage. (D) Weight loss rates of PLA and PET after 90 days of incubation with *S. ficaria* HfyG-1. (E) Surface morphology of PLA and PET plastics after treatment with *S. ficaria* HfyG-1, showing clear evidence of microbial degradation. Scale bars, 50 μm. (F) Fourier transform infrared spectra of PLA and PET plastics before treatment (black curves, control) and after inoculation with *S. ficaria* HfyG-1 (red curves, treatment). TM, template modeling.

representing new species, and approximately 99% of this catalog includes novel candidate plastic-degrading bacteria. The candidate plastic-degrading bacterial catalog provided in this study not only greatly expands the resources of plastic degradation but also complements microbial resources, such as the genomes from the LakePulse catalog,[50] which is limited by the global coverage of lake ecosystems.

To understand the actual applicability of candidate plastic-degrading bacteria to the environment, it is necessary to consider their plastic-degrading potential, ecological risk, adaptation capacity, and likely competitiveness in the environment. Excellent candidate plastic-degrading bacteria should not only adapt to living on plastic surfaces and be able to compete for nutrients but also have high plastic degradation potential and pose little risk to humans, animals, plants, or the environment. Therefore, we developed a novel computational framework to assess the priority for potential environmental applications in the environment on the basis of these 4 factors. Using this framework, we classified 165 experimentally verified plastic-degrading strains into 4 priority ranks and found that most of these degraders were indeed high priority; however, some of these strains were identified as LOW priority or opportunistic pathogens, highlighting the usefulness of this framework for minimizing the ecological risk of any future application of plastic-degrading strains.

Importantly, our study demonstrated that only 3.7% of these plastic-degrading bacteria were readily cultivable by comparing 400,596 reference genomes from the RefSeq database and GenBank; thus, enriching, isolating, and culturing such strains is the greatest obstacle to their application. To address this dilemma, based on the genomic data of LPDMAGs and their priority index analysis, we provide an enrichment and isolation strategy for EXCELLENT candidate plastic-degrading bacteria. Several studies have indicated that the functional annotation of MAGs is critical for metabolic pathway reconstruction and can be used to help with their cultivation.[51–54] For example, Renesto et al.[55] revealed the metabolic deficiencies of *Tropheryma whipplei* MAGs in amino acid biosynthesis and isolated them by modifying the growth medium via the addition of nine necessary amino acids. Our study reconstructed the essential nutrient biosynthesis pathway of candidate plastic-degrading bacteria and demonstrated that most high-priority degraders were auxotrophs. This may be because auxotrophy is an adaptive trait, and it might be particularly advantageous when essential metabolites can be competitively obtained from the 1surrounding environment or from nearby cells.[55] There is some evidence for

www.the-innovation.org

auxotrophy-mediated nutrient exchange in microbial communities with hydrocarbon degradation potential,[42] which further supports our findings.

Guided by the metabolic profiles of high-priority candidate degraders, we formulated eight template media and successfully enriched PET- and PLA-degrading bacteria from Xiazhu Lake. Reported degradation efficiencies vary widely among strains and conditions—for example, *Ideonella sakaiensis* 201-F6 almost completely degraded low-crystallinity PET films in 42 days,[12] *Bacillus* strains achieved 30%–40% degradation within a month,[56] and *Amycolatopsis* HT-32 degraded 60% PLA in 2 weeks, whereas natural soil degradation was extremely slow (0.23% in 12 months).[57] In our assays, the mixed consortium degraded 33.1% PET and 37.1% PLA within 90 days, and the pure culture of *S. ficaria* HfyG-1 achieved 36.4% and 35.4% degradation, respectively. Although slightly less efficient than optimized laboratory strains, HfyG-1 is distinctive in degrading both PET and PLA under identical conditions and was identified as an excellent, environmentally resilient degrader suitable for safe and sustainable plastic management.

Moreover, although this study integrates large-scale metagenomic analyses with experimental validation, several limitations should be acknowledged. The identification of plastic-degrading microbes and enzymes is primarily based on genome-resolved predictions and computational screening, and therefore reflects putative degradation potential rather than definitive enzymatic activity. Future studies incorporating comprehensive biochemical characterization of individual enzymes and systematic validation of candidate strains will be essential to fully resolve their functional roles. Meanwhile, the current prioritization framework was developed mainly for millimeter- to centimeter-scale plastic fragments, which provide sufficient surface area for microbial colonization and measurable degradation. Smaller microplastics (<300 μm) and nanoplastics exhibit distinct physicochemical properties, including altered surface area-to-volume ratios, diffusion constraints, and enzymatic accessibility, and were beyond the scope of this study. Despite these limitations, our work provides the first global, genome-resolved catalog of candidate plastic-degrading microbes in lake ecosystems and establishes a data-driven framework that links degradation potential with ecological viability, offering a systematic and transferable strategy for future exploration and application of plastic-degrading microbial resources.

In conclusion, this study presents a global catalog of candidate plastic-degrading microbes and enzymes across lake ecosystems through large-scale metagenomic analysis. We developed a computational framework to categorize candidate degraders based on their genomic and ecological traits and reconstructed their essential nutrient biosynthesis pathways, facilitating targeted enrichment and cultivation from the plastisphere. By integrating genome-resolved analyses with experimental validation, we revealed that plastic degradation is likely driven by synergistic microbial networks rather than single degraders, both in natural communities and in microcosms. By designing customized media, we successfully isolated *S. ficaria* HfyG-1, which is capable of degrading PLA and PET. Together, these findings establish a proof-of-concept framework for identifying and harnessing plastic-degrading microbial consortia, offering a new perspective for microbial resource exploration in natural environments.

RESOURCE AVAILABILITY

Materials availability

This study did not generate new unique materials.

Data and code availability

- Raw metagenomics data are publicly available in the ENA (https://ebi.ac.uk/ena/) and NCBI (https://ncbi.nlm.nih.gov/sra/) databases.
- The raw metatranscriptomic datasets used in this study were retrieved from public repositories under accession numbers SRA: PRJNA691101, PRJNA1089960, PRJNA1161448, and ENA: PRJEB61515.
- Global spatial covariates, the links of which are presented in Table S10, are publicly available.
- The whole-genome sequencing data of an isolated plastic-degrading strain are available through the NCBI Sequence Read Archive under BioProject ID SRA: PRJNA1144214.
- All the data needed to evaluate the conclusions in the paper are presented in the paper and/or the supplemental information.
- All the scripts and codes for gene annotation, abundance calculation, machine learning, statistical analysis, and visualization used in this study are available online at https://github.com/QiZhang11/Lake-plastic-degraders.

FUNDING AND ACKNOWLEDGMENTS

This work was supported by the National Natural Science Foundation of China (22241603-3, 42307158, and 42507185), the Quzhou Joint Fund of the Zhejiang Provincial Natural Science Foundation of China (LQZSZ26B070002), the Project of the Scientific and Technological Program of Shaoxing (2024A13013), and the Ningbo Public Welfare Key Science and Technology Plan Project (2023S011). The funders had no role in study design, data collection and analysis, decision to publish, or preparation of the manuscript.

AUTHOR CONTRIBUTIONS

Q.Z. and Zhenyan Zhang designed the study with guidance from H.F.Q. and Y.-G.Z. Q.Z. wrote the first draft of the manuscript and Y.-G.Z., H.F.Q., M.C.R., L.C., and D.Z. contributed substantially to revisions. Q.Z., T.Z.W., G.Y.Q., M.K.J., and B.F.C. contributed to the all functional annotation of metagenome-assemble genomes. Q.Z., T.Z.W., M.X.W., and Zhenyan Zhang performed all the metagenomic analysis. Q.Z., Zhenyan Zhang, and T.L. were responsible for the machine learning model construction and related data analysis. Q.Z. and Y.T.Y. perform all the microbial metabolic profiles reconstruction based on the genome analysis. Q.Z., G.Y.Q., and Ziyao Zhang experimented target enrichment plastic degraders. Q.Z. performed the visualization of all data and the artistic design of all figures. H.F.Q., Q.Z., Zhenyan Zhang, and D.Z. acquired funding for this project. All authors contributed to the manuscript and approved the final version.

DECLARATION OF INTERESTS

The authors declare no competing interests.

SUPPLEMENTAL INFORMATION

It can be found online at https://doi.org/10.1016/j.xinn.2026.101338.

REFERENCES

1. MacLeod, M., Arp, H.P.H., Tekman, M.B. et al. (2021). The global threat from plastic pollution. *Science* **373**:61–65. DOI:10.1126/science.abg5433
2. Rochman, C.M. and Hoellein, T. (2020). The global odyssey of plastic pollution. *Science* **368**:1184–1185. DOI:10.1126/science.abc4428
3. Mennekes, D. and Nowack, B. (2023). Predicting microplastic masses in river networks with high spatial resolution at country level. *Nat. Water* **1**:523–533. DOI:10.1038/s44221-023-00090-9
4. Nava, V., Chandra, S., Aherne, J. et al. (2023). Plastic debris in lakes and reservoirs. *Nature* **619**:317–322. DOI:10.1038/s41586-023-06168-4
5. Haney, J. and Rochman, C.M. (2025). Plastic pollution has the potential to alter ecological and evolutionary processes in aquatic ecosystems. *Nat. Ecol. Evol.* **9**:762–768. DOI:10.1038/s41559-025-02678-8
6. Fan, J., Zou, L., Duan, T. et al. (2022). Occurrence and distribution of microplastics in surface water and sediments in China's inland water systems: A critical review. *J. Clean. Prod.* **331**:129968. DOI:10.1016/j.jclepro.2021.129968
7. Lear, G., Kingsbury, J.M., Franchini, S. et al. (2021). Plastics and the microbiome: impacts and solutions. *Environ. Microbiome* **16**:2. DOI:10.1186/s40793-020-00371-w
8. Guo, F., Liu, B., Zhao, J. et al. (2024). Effects of polyethylene, polylactic acid, and tire particles on the sediment microbiome and metabolome at high and low temperatures. *Appl. Environ. Microbiol.* **90**:e02016-23. DOI:10.1128/aem.02016-23
9. Naying, L., Xintong, M., Bo, Z. et al. (2025). Potential ecological risk of microplastics contamination to environment in protect area lakes. *J. Hazard. Mater.* **485**:136863. DOI:10.1016/j.jhazmat.2024.136863
10. Zhou, M., Luo, C., Zhang, J. et al. (2025). Potential risk of microplastics in plateau karst lakes: Insights from metagenomic analysis. *Environ. Res.* **270**:120984. DOI:10.1016/j.envres.2025.120984
11. Ghasemlou, M., Adhikari, B., Naebe, M. et al. (2025). Bioengineering strategies to promote plastic circularity. *Nat. Rev. Bioeng.* **3**:353–354. DOI:10.1038/s44222-025-00287-5
12. Yoshida, S., Hiraga, K., Takehana, T. et al. (2016). A bacterium that degrades and assimilates poly(ethylene terephthalate). *Science* **351**:1196–1199. DOI:10.1126/science.aad6359
13. Buchholz, P.C.F., Feuerriegel, G., Zhang, H. et al. (2022). Plastics degradation by hydrolytic enzymes: The plastics-active enzymes database-PAZy. *Proteins* **90**:1443–1456. DOI:10.1002/prot.26325
14. Gambarini, V., Pantos, O., Kingsbury, J.M. et al. (2022). PlasticDB: a database of microorganisms and proteins linked to plastic biodegradation. *Database* **2022**:baac008. DOI:10.1093/database/baac008
15. Ferrer, M., Martínez-Martínez, M., Bargiela, R. et al. (2016). Estimating the success of enzyme bioprospecting through metagenomics: current status and future trends. *Microb. Biotechnol.* **9**:22–34. DOI:10.1111/1751-7915.12309
16. Chow, J., Perez-Garcia, P., Dierkes, R. et al. (2023). Microbial enzymes will offer limited solutions to the global plastic pollution crisis. *Microb. Biotechnol.* **16**:195–217. DOI:10.1111/1751-7915.14135
17. Zrimec, J., Kokina, M., Jonasson, S. et al. (2020). Plastic-degrading potential across the global microbiome correlates with recent pollution trends. *mBio* **12**:e0215521. DOI:10.1101/2020.12.13.422558

18. Tyson, G.W., Chapman, J., Hugenholtz, P. et al. (2004). Community structure and metabolism through reconstruction of microbial genomes from the environment. *Nature* **428**:37–43. DOI:10.1038/nature02340
19. Hug, L.A., Baker, B.J., Anantharaman, K. et al. (2016). A new view of the tree of life. *Nat. Microbiol.* **1**:16048. DOI:10.1038/nmicrobiol.2016.48
20. Sun, Y., Wu, M., Zang, J. et al. (2023). Plastisphere microbiome: Methodology, diversity, and functionality. *iMeta* **2**:e101. DOI:10.1002/imt2.101
21. Wang, J., Qin, X., Guo, J. et al. (2020). Evidence of selective enrichment of bacterial assemblages and antibiotic resistant genes by microplastics in urban rivers. *Water Res.* **183**:116113. DOI:10.1016/j.watres.2020.116113
22. Rillig, M.C., Kim, S.W. and Zhu, Y.-G. (2024). The soil plastisphere. *Nat. Rev. Microbiol.* **22**:64–74. DOI:10.1038/s41579-023-00967-2
23. Zhu, D., Ma, J., Li, G. et al. (2022). Soil plastispheres as hotspots of antibiotic resistance genes and potential pathogens. *ISME J.* **16**:521–532. DOI:10.1038/s41396-021-01103-9
24. Gupta, K.K. and Devi, D. (2020). Characteristics investigation on biofilm formation and biodegradation activities of Pseudomonas aeruginosa strain ISJ14 colonizing low density polyethylene (LDPE) surface. *Heliyon* **6**:e04398. DOI:10.1016/j.heliyon.2020.e04398
25. Zhang, X., Feng, X., Lin, Y. et al. (2023). Degradation of polyethylene by Klebsiella pneumoniae Mk-1 isolated from soil. *Ecotoxicol. Environ. Saf.* **258**:114965. DOI:10.1016/j.ecoenv.2023.114965
26. Azura Azami, N., Ira Aryani, W., Aik-Hong, T. et al. (2019). Purification and characterization of new bio-plastic degrading enzyme from *Burkholderia cepacia* DP1. *Protein Expr. Purif.* **155**:35–42. DOI:10.1016/j.pep.2018.10.008
27. Li, D., Liu, C.M., Luo, R. et al. (2015). MEGAHIT: an ultra-fast single-node solution for large and complex metagenomics assembly via succinct de Bruijn graph. *Bioinformatics* **31**:1674–1676. DOI:10.1093/bioinformatics/btv033
28. Langmead, B. and Salzberg, S.L. (2012). Fast gapped-read alignment with Bowtie 2. *Nat. Methods* **9**:357–359. DOI:10.1038/nmeth.1923
29. Li, H., Handsaker, B., Wysoker, A. et al. (2009). The Sequence Alignment/Map format and SAMtools. *Bioinformatics* **25**:2078–2079. DOI:10.1093/bioinformatics/btp352
30. Alneberg, J., Bjarnason, B.S., de Bruijn, I. et al. (2014). Binning metagenomic contigs by coverage and composition. *Nat. Methods* **11**:1144–1146. DOI:10.1038/nmeth.3103
31. Wu, Y.W., Simmons, B.A. and Singer, S.W. (2016). MaxBin 2.0: an automated binning algorithm to recover genomes from multiple metagenomic datasets. *Bioinformatics* **32**:605–607. DOI:10.1093/bioinformatics/btv638
32. Kang, D.D., Li, F., Kirton, E. et al. (2019). MetaBAT 2: an adaptive binning algorithm for robust and efficient genome reconstruction from metagenome assemblies. *PeerJ* **7**:e7359. DOI:10.7717/peerj.7359
33. Parks, D.H., Imelfort, M., Skennerton, C.T. et al. (2015). CheckM: assessing the quality of microbial genomes recovered from isolates, single cells, and metagenomes. *Genome Res.* **25**:1043–1055. DOI:10.1101/gr.186072.114
34. Eddy, S.R. (1998). Profile hidden Markov models. *Bioinformatics* **14**:755–763. DOI:10.1093/bioinformatics/14.9.755
35. Yu, T., Cui, H., Li, J.C. et al. (2023). Enzyme function prediction using contrastive learning. *Science* **379**:1358–1363. DOI:10.1126/science.adf2465
36. Tu, Q., Lin, L., Cheng, L. et al. (2019). NCycDB: a curated integrative database for fast and accurate metagenomic profiling of nitrogen cycling genes. *Bioinformatics* **35**:1040–1048. DOI:10.1093/bioinformatics/bty741
37. Yu, X., Zhou, J., Song, W. et al. (2021). SCycDB: A curated functional gene database for metagenomic profiling of sulphur cycling pathways. *Mol. Ecol. Resour.* **21**:924–940. DOI:10.1111/1755-0998.13306
38. Zheng, B., Zhu, Y., Sardans, J. et al. (2018). QMEC: a tool for high-throughput quantitative assessment of microbial functional potential in C, N, P, and S biogeochemical cycling. *Sci. China Life Sci.* **61**:1451–1462. DOI:10.1007/s11427-018-9364-7
39. Shi, Z., Yin, H., Van Nostrand, J.D. et al. (2019). Functional Gene Array-Based Ultrasensitive and Quantitative Detection of Microbial Populations in Complex Communities. *mSystems* **4**:e00296-19. DOI:10.1128/mSystems.00296-19
40. Nadell, C.D., Drescher, K. and Foster, K.R. (2016). Spatial structure, cooperation and competition in biofilms. *Nat. Rev. Microbiol.* **14**:589–600. DOI:10.1038/nrmicro.2016.84
41. Du Toit, A. (2018). Increasing virulence factors. *Nat. Rev. Microbiol.* **16**:658. DOI:10.1038/s41579-018-0091-3
42. Zhang, Z., Zhang, Q., Wang, T. et al. (2022). Assessment of global health risk of antibiotic resistance genes. *Nat. Commun.* **13**:1553. DOI:10.1038/s41467-022-29283-8
43. Bowers, R.M., Kyrpides, N.C., Stepanauskas, R. et al. (2017). Minimum information about a single amplified genome (MISAG) and a metagenome-assembled genome (MIMAG) of bacteria and archaea. *Nat. Biotechnol.* **35**:725–731. DOI:10.1038/nbt.3893
44. Almeida, A., Nayfach, S., Boland, M. et al. (2021). A unified catalog of 204,938 reference genomes from the human gut microbiome. *Nat. Biotechnol.* **39**:105–114. DOI:10.1038/s41587-020-0603-3
45. Bartell, J.A., Blazier, A.S., Yen, P. et al. (2017). Reconstruction of the metabolic network of Pseudomonas aeruginosa to interrogate virulence factor synthesis. *Nat. Commun.* **8**:14631. DOI:10.1038/ncomms14631
46. Lopez, M. (2020). Unsupervised K-Means Clustering Algorithm. *IEEE Access* **8**:80716–80727. DOI:10.1109/ACCESS.2020.2988796
47. OECD (2022). Global Plastics Outlook (OECD). stats.oecd.org/viewhtml.aspx?datasetcode=PLASTIC_USE_10&lang=en
48. Williamson, C.E., Dodds, W., Kratz, T.K. et al. (2008). Lakes and streams as sentinels of environmental change in terrestrial and atmospheric processes. *Front. Ecol. Environ.* **6**:247–254. DOI:10.1890/070140
49. Zhou, L., Sang, S., Li, J. et al. (2023). From waste to resource: Metagenomics uncovers the molecular ecological resources for plastic degradation in estuaries of South China. *Water Res.* **242**:120270. DOI:10.1016/j.watres.2023.120270
50. Garner, R.E., Kraemer, S.A., Onana, V.E. et al. (2023). A genome catalogue of lake bacterial diversity and its drivers at continental scale. *Nat. Microbiol.* **8**:1920–1934. DOI:10.1038/s41564-023-01435-6
51. Liu, S., Moon, C.D., Zheng, N. et al. (2022). Opportunities and challenges of using metagenomic data to bring uncultured microbes into cultivation. *Microbiome* **10**:76. DOI:10.1186/s40168-022-01272-5
52. Tyson, G.W., Lo, I., Baker, B.J. et al. (2005). Genome-directed isolation of the key nitrogen fixer Leptospirillum ferrodiazotrophum sp. nov. from an acidophilic microbial community. *Appl. Environ. Microbiol.* **71**:6319–6324. DOI:10.1128/aem.71.10.6319-6324.2005
53. Pope, P.B., Smith, W., Denman, S.E. et al. (2011). Isolation of Succinivibrionaceae implicated in low methane emissions from *Tammar wallabies*. *Science* **333**:646–648. DOI:10.1126/science.1205760
54. Lugli, G.A., Milani, C., Duranti, S. et al. (2019). Isolation of novel gut bifidobacteria using a combination of metagenomic and cultivation approaches. *Genome Biol.* **20**:96. DOI:10.1186/s13059-019-1711-6
55. Renesto, P., Crapoulet, N., Ogata, H. et al. (2003). Genome-based design of a cell-free culture medium for Tropheryma whipplei. *Lancet* **362**:447–449. DOI:10.1016/s0140-6736(03)14071-8
56. Dhaka, V., Singh, S., Ramamurthy, P.C. et al. (2023). Biological degradation of polyethylene terephthalate by rhizobacteria. *Environ. Sci. Pollut. Res. Int.* **30**:116488–116497. DOI:10.1007/s11356-022-20324-9
57. Pranamuda, H., Tokiwa, Y. and Tanaka, H. (1997). Polylactide Degradation by an Amycolatopsis sp. *Appl. Environ. Microbiol.* **63**:1637–1640. DOI:10.1128/aem.63.4.1637-1640.1997

The Innovation, Volume 7

Supplemental Information

Potential plastic biodegradation in lakes worldwide

Qi Zhang, Zhenyan Zhang, Ziyao Zhang, Guoyan Qin, Mingkang Jin, Bingfeng Chen, Yitian Yu, Tingzhang Wang, Meixia Wang, Tao Lu, Dong Zhu, Li Cui, Haifeng Qian, Matthias C. Rillig, and Yong-Guan Zhu

Supplemental Information

Supplemental methods

Datasets

We searched the European Nucleotide Archive (ENA; https://ebi.ac.uk/ena/) and NCBI Sequence Read Archive (https://ncbi.nlm.nih.gov/sra/) for metadata from 01-16-2023, using the terms “Lake,” “Metagenome,” and “Freshwater.” We used the following metagenome selection criteria to minimize possible bias: (i) pond samples, such as artificial or natural ponds, were excluded; (ii) samples were not collected from aquatic organisms and sediments or lakes adjacent to pollution sources, extreme climates, treatments, and inaccurate coordinate records; (iii) only paired-end sequencing reads in FASTQ format were included, and the average read length of the metagenomes exceeded 100 base pairs. We collected 715 metagenomic samples from 95 lakes located across five continents and 19 countries.

Metagenomic assemblies and reconstruction of LPDMAGs

Metagenomic assemblies were reconstructed via MEGAHIT v1.2.9 with metawrap assembly and metawrap binning commands.[1] The reads were mapped to contigs via Bowtie 2 (v.2.3.5.1) with default parameters,[2] and the resulting alignment files were sorted and indexed via SAMtools (v1.9).[3] The sorted BAM file was used to bind the contigs via a combination of CONCOCT [4] (v.1.0.0), MaxBin 2.0 (v.2.2.6),[5] and metaBAT2 (v.2.12.1) with default parameters to identify the MAGs.[6] During refinement, the completeness and contamination of all MAGs were estimated via the lineage-specific workflow via CheckM (v1.0.11) to generate MAGs with ≥ 50% completeness and < 10% contamination.[7] This quality filtering step was applied to minimize the influence of fragmented assemblies and chimeric bins, which can introduce noise into functional prediction when short or misassembled sequences are considered. Hidden Markov model (HMM) searches were then performed across all retained MAGs. To further reduce false-positive functional assignments associated with short contigs and assembly artifacts, we restricted candidate hits to contigs longer than 10 kb. In addition, we required that the taxonomic affiliation of each contig harboring a candidate gene was consistent with the overall taxonomy of its

corresponding MAG, thereby ensuring taxonomic coherence between predicted functions and genome-level context. After filtering, we produced a total of 4,856 LPDMAGs and clustered them into 1,370 species-level genome bins on the basis of 95% ANI via dRep (v2.2.4).[8] The current methods for analyzing putative plastic-degrading enzymes result in similar problems of broadening the scope for identifying candidate plastic-degrading bacteria. To calculate the community assembly of these candidate degrading bacteria in the lake, we used CoverM (v0.7.0) to successfully identify a total of 765 candidate plastic-degrading taxa across all metagenomes.[9] Ribosomal and transfer RNAs were identified via the nhmmer function (part of HMMER 3) from Barrnap (v.0.9) and tRNAscan-SE (v.2.0.9) with default parameters, respectively.[10] The high-quality MAGs were defined as those with > 90% completeness, ≤ 5% contamination, ≥ 18/20 tRNA genes, and 5S, 16S, and 23S rRNA genes, and the remaining MAGs were classified as medium-quality MAGs.

Enzyme dataset and construction of HMMs

On the basis of published studies and databases,[11,12] we collected a dataset of 184 sequenced plastic-degrading enzyme-encoding genes spanning 10 plastic types (Dataset S11). Before model construction, each enzyme sequence was manually curated and verified by cross-checking its origin, substrate type, and supporting literature. Controversial or retracted entries were excluded to ensure reliability. All reference enzymes were further categorized into three functional groups according to polymer type: polyolefin-degrading enzymes (PE and PS); conventional polyester-degrading enzymes (PET and PU); and biodegradable plastic–degrading enzymes (PLA, PBAT, PHAs, PHB and PVA). To expand the sequence space for building the HMMs, we downloaded the UniProt TrEMBL database (on May 16, 2023) with more than 2.4 billion candidate protein sequences, which was used to obtain the representative sequences via BLAST (v2.6) with default settings, except for an E-value cutoff of 1e-10 and an identity cut off 90%.[13] We next extracted each sequence ID and removed redundant representative sequences via CD-HIT with default settings,[14,15] with the exception of a word size of 5, a cluster size of 5, and a sequence identity cutoff of 60%. Finally, we performed multisequence alignment of these

representative sequences via MAFFT and constructed a total of 413 HMMs (including all representative sequences) via HMMER v3.3 hmmbuild utility (http://hmmer.org/) with default settings.[16, 17]

We detected the genes from the predicted proteins of the metagenomes on the basis of the constructed HMM profiles via hmmsearch from HMMER v3.3, with an E value cut off below 1e-10. Only the lowest E value and bit score hits were retained for each gene in the metagenomes. To minimize potential false positives and enhance annotation accuracy, we first used the customized HMM models to scan all metagenomic contigs and identify candidate open reading frames (ORFs) that matched plastic-degrading enzyme profiles. The ORFs aligned to these HMM models were then further analyzed using the machine learning-based CLEAN model, which employs official open-source pretrained weights to predict Enzyme Commission (EC) numbers.[18] By comparing the predicted EC numbers with those of known plastic-degrading enzymes (Dataset S2), we validated functional consistency and refined the annotation. The combined HMM–machine learning pipeline ultimately identified 45,154 nonredundant enzyme hits across all metagenomes, covering ten major plastic types (biodegradation pathways, see Figure S18).

Annotation of functions in LPDMAGs and reference genomes

Antibiotic resistance genes in LPDMAGs (1,744 high-completeness MAGs) and bacterial genomes were annotated with CARD (v2020) using their recommended tool, RGI (v5.1.1),[19] with the default parameters for genomes. Pathogenic (hypervirulence) and virulence factors were annotated with the Pathogen-Host Interactions (www.phi-base.org/) and Virulence Factors of Bacterial Pathogens (mgc.ac.cn) databases using DIAMOND (v2.1.4; e-value ≤ 10^{-3}, identity: 70%, coverage: 60%).[20] Moreover, we annotated the potential for the biosynthesis and repair of biological membrane from LPDMAGs and reference genomes with a constructed database based on the UniprotKB database (http://www.uniprot.org/) with biological membrane-related genes. Furthermore, we manually compiled a comprehensive database of genes involved in carbon, nitrogen, phosphorus, and sulfur (CNPS) cycles from NCycDB,[21] SCycDB,[22] QMEC,[23] and GeoChip 5.0.[24] The potential ecological functions of the

LPDMAGs were annotated via the CNPS database created by BWA (v0.7.13).[3]

Reconstruction of the biosynthesis pathways of growth factors in nearly complete LPDMAGs

We manually reconstructed a total of 73 biosynthetic pathways with 299 proteins and 469 KO numbers, covering 41 essential growth factors (e.g., vitamins, amino acids, nucleotides, and coenzymes) for bacteria or archaea (Dataset S8). To ensure the integrity of the biosynthesis of each growth factor, we considered all pathways, such as cysteine biosynthesis, which was associated with three genes (M00021, M00338 and M00609), and biotin biosynthesis, which included the BioU, I, F and W pathways. Moreover, we also considered the biosynthesis of the intermediates required in the biosynthesis pathway of each growth factor, such as pimeloyl-ACP for biotin biosynthesis. We annotated 1,744 high-completeness MAGs with these biosynthetic pathways on the basis of the Kyoto Encyclopedia of Genes and Genomes database via DIAMOND (v2.1.4; e value ≤ 10^{-3}, identity: 70%, coverage: 60%).[20]

Calculation of a priority index of candidate plastic-degrading bacteria

We developed a novel computational framework to assess the priority of strains for potential application in the environment on the basis of four factors: plastic-degrading potential, ecological risk, adaptation capacity, and competition capacity. The number of HMM hits in each LPDMAG indicates the plastic-degrading potential. Biofilms, the predominant mode of microbial life, are considered successful evolutionary adaptation for bacteria. By growing in biofilms, plastic-degrading bacteria can withstand external stress (such as oxidative stress, nutrient deficiencies, etc.), improving their chances of survival and reproduction.[25] Once the degraders are adapted to the plastic surface environment, nutritional restriction becomes the biggest obstacle, and better potential degraders can effectively utilize nutrient elements.

First, we calculated the adaptation capacity of each candidate plastic-degrading bacteria as follows:

$$F_{(AC)} = \sum_{i=1}^{n} Nor(F_{(AC)}i) \tag{1}$$

where $F_{(AC)}$ is the adaptation capacity of candidate plastic-degrading MAGs.

We thus took the potential of participating in carbon, nitrogen, phosphorus, and sulfur cycles as an indicator of the competitive capacity of candidate plastic-degrading bacteria.

Next, we calculated the competitive capacity of each candidate plastic-degrading bacteria as follows:

$$F_{(CC)} = \sum_{i=1}^{n} Nor(F_{(CC)}i) \quad (2)$$

where $F_{(CC)}$ is the competitive capacity of candidate plastic-degrading MAGs.

In addition, pathogenic (hypervirulence) and virulence factors underpin the pathogenicity and toxicity of these candidate degraders to animals, plants, and humans,[26] and antibiotic resistance genes with high risk also indicate a health risk for humans,[27] all of which represented the ecological risk of candidate degraders.
We calculated the ecological risk of each candidate plastic-degrading bacteria as follows:

$$F_{(ER)} = \sum_{i=1}^{n} Nor(F_{(ER)}i) \quad (3)$$

Finally, in combination with the above three factors, we calculated the priority index (PI) of the candidate plastic-degrading bacteria as follows:

$$PI_{MAG} = DP \times 1/2F_{(AC)} \times 1/2F_{(CC)} \times F_{(ER)} \quad (4)$$

where DP is the count of putative plastic-degrading enzyme-encoding in each candidate plastic-degrading bacteria. High plastic degradation potential, no other risk burden to human beings, and the environment play key roles in the selection of candidate plastic-degrading bacteria. Accordingly, these were considered primary indicators in our computing framework (weight 100%), whereas the weights of the adaptation and competitive capacity indicators were reduced (50%), reflecting the reduced importance of these two factors. The priority index of the pathogen is directly set to 0. The K-means algorithm divided the candidate plastic-degrading bacteria (number not strictly even but dissimilar in each rank) into EXCELLENT (n = 11), HIGH (n = 50), MEDIUM (n = 186), and LOW (n = 1,497).

Enrichment and isolation of plastic-degrading bacteria

We deployed samples of PLA and PET plastic for *in situ* enrichment in Xiazhu

Lake, Huzhou, China (30.51581° N, 120.04847° E), for six months and subsequently retrieved these plastics for collecting the PLA and PET plastisphere samples. Each plastic sample was rinsed three times with deionized water to remove any unattached organisms. Plastisphere samples were then collected via a sterile brush and blade, transferred into sterile centrifuge tubes containing 10 mL of PBS, and vortexed for 5 minutes. The plastic debris was removed from the resulting solution, and the remaining suspension was used as a microbial inoculum. The inoculum was transferred to a 250-mL flask containing 0.1 g of pre-cleaned PLA and PET particles (irregularly spherical, diameter of 120–200 μm) and 100 mL of MSM-based customized liquid medium. The test plastics were purchased from Dongguan Kaixuan Plastic Technology Co., Ltd., sterilized in 75% ethanol for 20 minutes, washed three times with sterile water, and air-dried on a sterile workbench. After weighing approximately 0.1g of each sample, the particles were placed in small Petri dishes and dried in a laminar flow hood. Subsequently, they were sterilized under UV light for 3 hours. After incubating at 28°C for 3 days, no colonies were observed around the particles in the culture medium, making them suitable for use in subsequent experiments.

To isolate plastic-degrading microorganisms, the plastic sheets were cut into 2 cm × 2 cm pieces, which were then suspended in 10 mL PBS solution and vortexed for 5 minutes. After removing the plastic pieces from the mixture, the remaining suspension was used as the inoculum and transferred into a 250 mL flask containing approximately 0.1g of pre-treated PET and PLA particles and 100 mL mineral salt medium (MSM). The experimental group was supplemented with eight different specialized media containing various growth factors following the recommended dosages of each essential growth factor (Dataset S9),[28,29] while the control group consisted of MSM without added growth factors. The PET and PLA particles were sterilized in 75% ethanol for 20 minutes, washed three times with sterile water, and dried in a laminar flow hood. Before the experiment, the sterilized PET and PLA particles were weighed. The microorganisms were cultured for 60 days at 30 °C in a shaking incubator (150 rpm), with subculturing every 15 days. A 1 mL aliquot of the

mixed bacterial culture was transferred into 5 mL Luria-Bertani medium and incubated for 24 hours. Then, 100 μL of the Luria-Bertani culture was transferred into a 1.5 mL sterile centrifuge tube containing sterile water, mixed, and used to prepare serial dilutions (10^{-1}, 10^{-2}, 10^{-3}, 10^{-4}, 10^{-5}, and 10^{-6}) to reduce the bacterial concentration. An appropriate volume of the diluted solution was inoculated onto agar plates using sterile pipette tips, and the bacteria were spread using sterile glass beads. The inoculated plates were incubated at an appropriate temperature (30 °C) for 24 hours, and bacterial colony growth was observed. Colonies that were consistent in appearance, color, and size (e.g., shape, size, and color) were selected as independent single colonies. A total of 53 independent colonies were recorded and then subcultured onto fresh agar plates, repeating the process 2-3 times to ensure the isolation of pure colonies (Figure S15).

Whole-genome sequencing of an isolated plastic-degrading strain

High-molecular-weight genomic DNA was extracted from late-log-phase cultures using the Cetyl Trimethyl Ammonium Bromide method with lysozyme digestion (10 mg/mL, 37 °C, 1 h). DNA quality was verified by Nanodrop (OD260/280 > 1.8) and Qubit fluorometry (Thermo Fisher). Illumina libraries (insert size: 394–441 bp) were constructed using the NEBNext Ultra II DNA Library Prep Kit and sequenced on the Illumina NovaSeq 6000 platform (Majorbio BioPharm Technology Co., Ltd., Shanghai) with 150-bp paired-end reads targeting ≥100× coverage. Raw reads were quality controlled using FASTP v0.20.0 (parameters: -q 20 -u 30 -l 50).[30] De novo assembly was performed using SOAPdenovo v2.04 (kmer = 55) with GapCloser v1.12 for scaffold refinement [1]. Assembly quality was assessed via BUSCO v5.4.7 (Bacteria odb10 dataset; completeness > 98%).[31] For gene prediction, Prodigal v2.6.3 was used, and for tRNA/rRNA identification, Barrnap (v.0.9) and tRNAscan-SE (v.2.0.9) with default parameters were used.[10] The database and thresholds for function annotation are consistent with those in the section "Annotation of functions in LPDMAGs and reference genomes". The whole-genome features of *Serratia ficaria* HfyG-1 were shown in Figure S19.

Full-length 16S rRNA sequencing and bioinformatics analysis

For the PLA and PET degrader isolations, culture broths collected on Day 30 and Day 60 were used for DNA extraction with the FastDNA® Spin Kit for Soil (MP Biomedicals) following the manufacturer's protocol. DNA purity and concentration were assessed with a Qubit fluorometer. The nearly full-length 16S rRNA gene was amplified using primers 27F (5′-AGAGTTTGATCMTGGCTCAG-3′) and 1492R (5′-TACGGYTACCTTGTTACGACTT-3′), and libraries were subsequently sequenced on the Majorbio Cloud Platform (Shanghai, China). Raw reads were processed with the VSEARCH workflow: quality filtering, dereplication, chimera removal (UCHIME), and OTU clustering (typically at 97% sequence identity), yielding representative sequences and an OTU table. Taxonomic annotation was performed against the SILVA reference database (release 138), and the resulting species/OTU table was used for downstream community analyses.

Verification test of plastic degradation

Plastisphere samples from Xiazhu Lake in Huzhou, China, were cultured for 60 days using PLA and PET as the sole carbon sources. The type4 cultured bacterial community, which was the most significantly enriched, was used to verify the degradation ability of plastics. Subsequently, the cleaned PLA and PET particles were inoculated with the type4 cultured bacterial community and *S. ficaria* HfyG-1 strain solution (four treatment groups, three biological replicates) and sterile water (four control groups, three biological replicates) for a duration of 90 days. After 90 days, the PLA and PET particles were washed three times with sterile water and air-dried in a sterile environment. The weight of each plastic sample was then recorded. Weight loss, the most straightforward indicator of plastic degradation, was calculated via Equations (5) and:[31]

$$\text{Weight loss} = \frac{W_{initial(g)} - W_{post(g)}}{W_{initial(g)}} \times 100\% \qquad (5)$$

where $W_{\text{initial(g)}}$ represents the initial weight and $W_{\text{post(g)}}$ represents the weight after treatment. Three pieces of plastic were weighed and averaged.

Furthermore, SEM images provide a close-up view of the plastic film surfaces; atomic force microscopy was used to observe the plastic surface topographical

changes and calculate the roughness.[32] Fourier transform infrared spectroscopy was used to assess the degradation of plastics by measuring the absorption of infrared radiation at specific wavelengths, which corresponds to the vibration modes of the chemical bonds.

Statistical analysis and visualization

The data are presented as the means ± standard errors of the means. We tested for differences via multiple methods, as described in the main text. The Mann–Whitney U test and Kruskal–Wallis test were used for pairwise comparisons between two or more groups via GraphPad Prism 8. Principal coordinate analyses and Adonis tests were performed via R (v3.6.3), and heatmaps were generated via TBtools (v1.113) and R (v3.6.3). The classification and regression models were built, and the accuracy was assessed *via random forest regression* and *forest.score* functions in the 'sklearn' Python package (v1.1.3). Venn diagrams were generated via EVenn (http://www.ehbio.com/test/venn; v1.0). Data figures were created via GraphPad Prism 8 and R (v3.6.3), and Adobe Illustrator 2020 was used to format the figures. The phylogeny was built via the 400 universal PhyloPhlAn markers via PhyloPhlAn (v3.0.60),[33] FastTree (v2.1.10),[34] and RAxML (v8.1.12) with parameters.[35] The best tree refined by RAxML is generated via ggtree (v3.2.1) and iTOL (v6.8.1).[36] All schematic diagrams and art elements were drawn via BioRender (https://app.biorender.com/; v1.0) with full publishing rights.

Supplemental figures

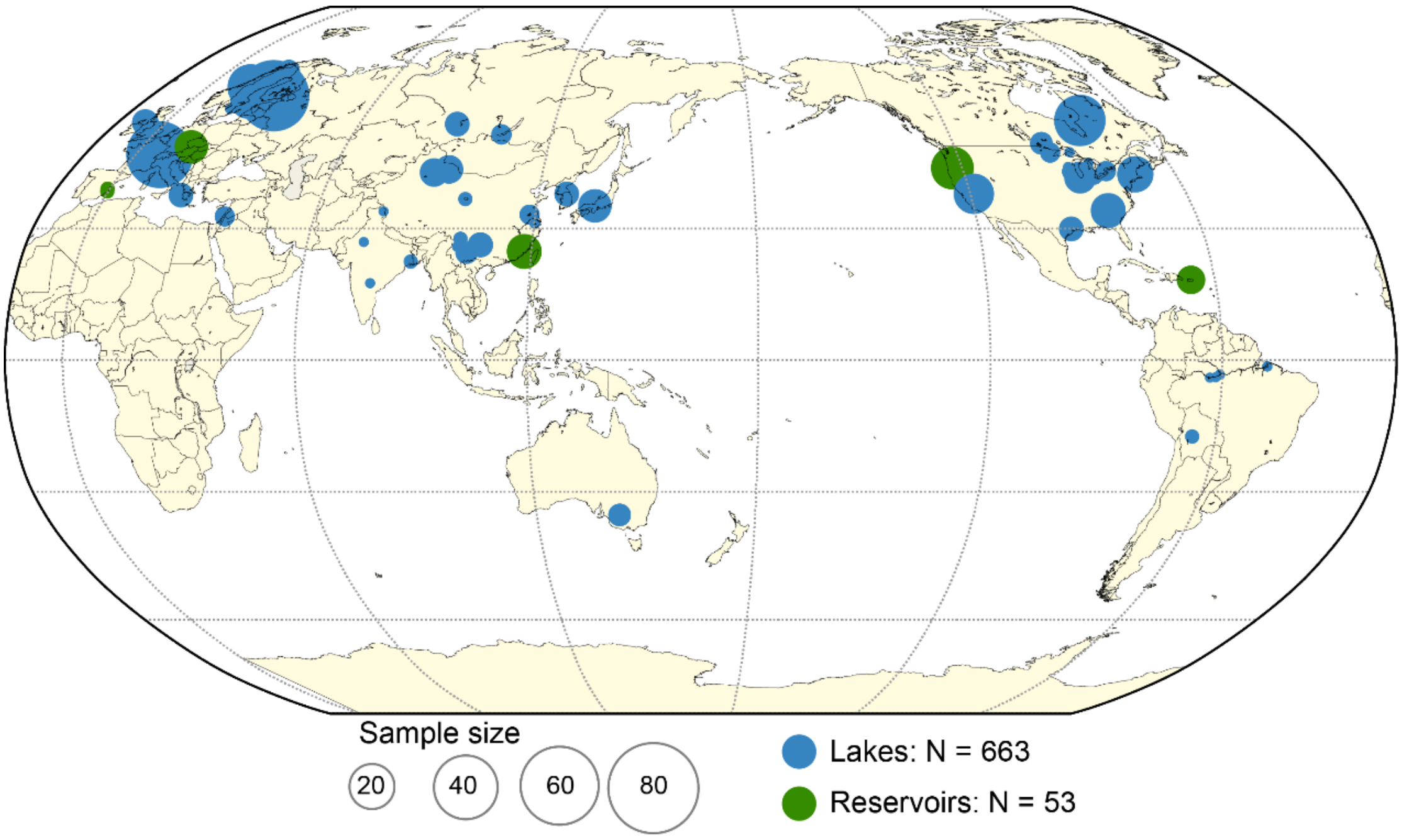

Figure S1. Geographical distribution of metagenomic sequencing data Circle size indicates sample numbers. Blue and green circles indicate the lake and reservoir samples, respectively. More details can be found in Dataset S1.

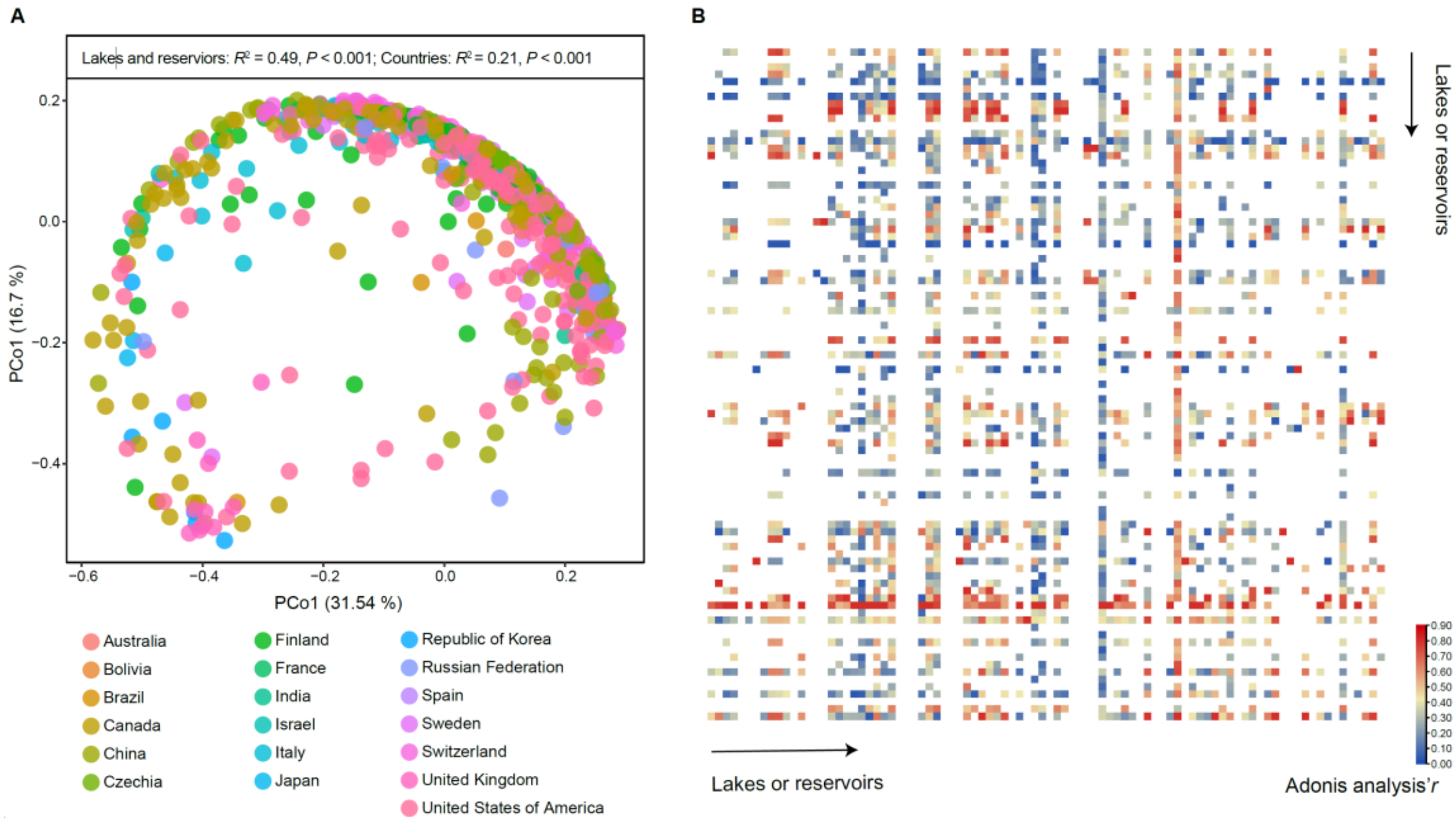

Figure S2. Geographical distribution of microbial plastic-degrading potential of all samples (A) Principal co-ordinates analysis with Bray-Curtis distance showed higher variations of microbial plastic-degrading potential between lakes and reservoirs than countries. (B) Pairwise Adonis analysis showed the distribution of candidate plastic-degrading enzyme-coding genes significantly different among all lakes and reservoirs. The gradient colour scale from red to blue indicates the Adonis analysis' *r* from high to low.

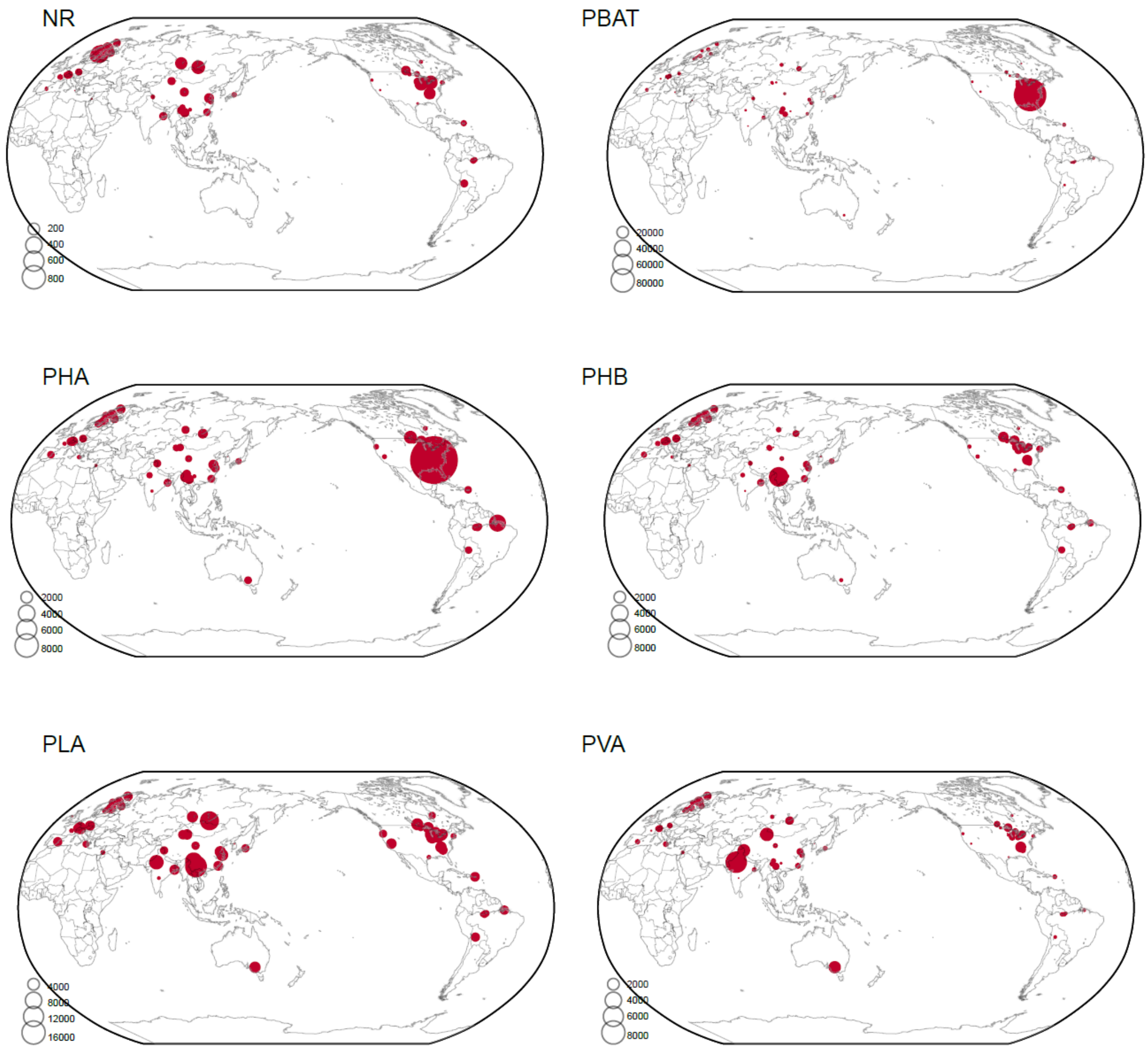

Figure S3. Geographical distribution of biodegradable plastic-degrading potential of microbes in lakes NR, natural rubber; PBAT, polybutylene adipate terephthalate; PHAs, poly-hydroxyalkanoate; PHB, polyhydroxybutyrate; PLA, polylactic acid; PVA, polyvinyl alcohol. The size of the circle indicates the abundance of candidate plastic-degrading enzyme-coding genes.

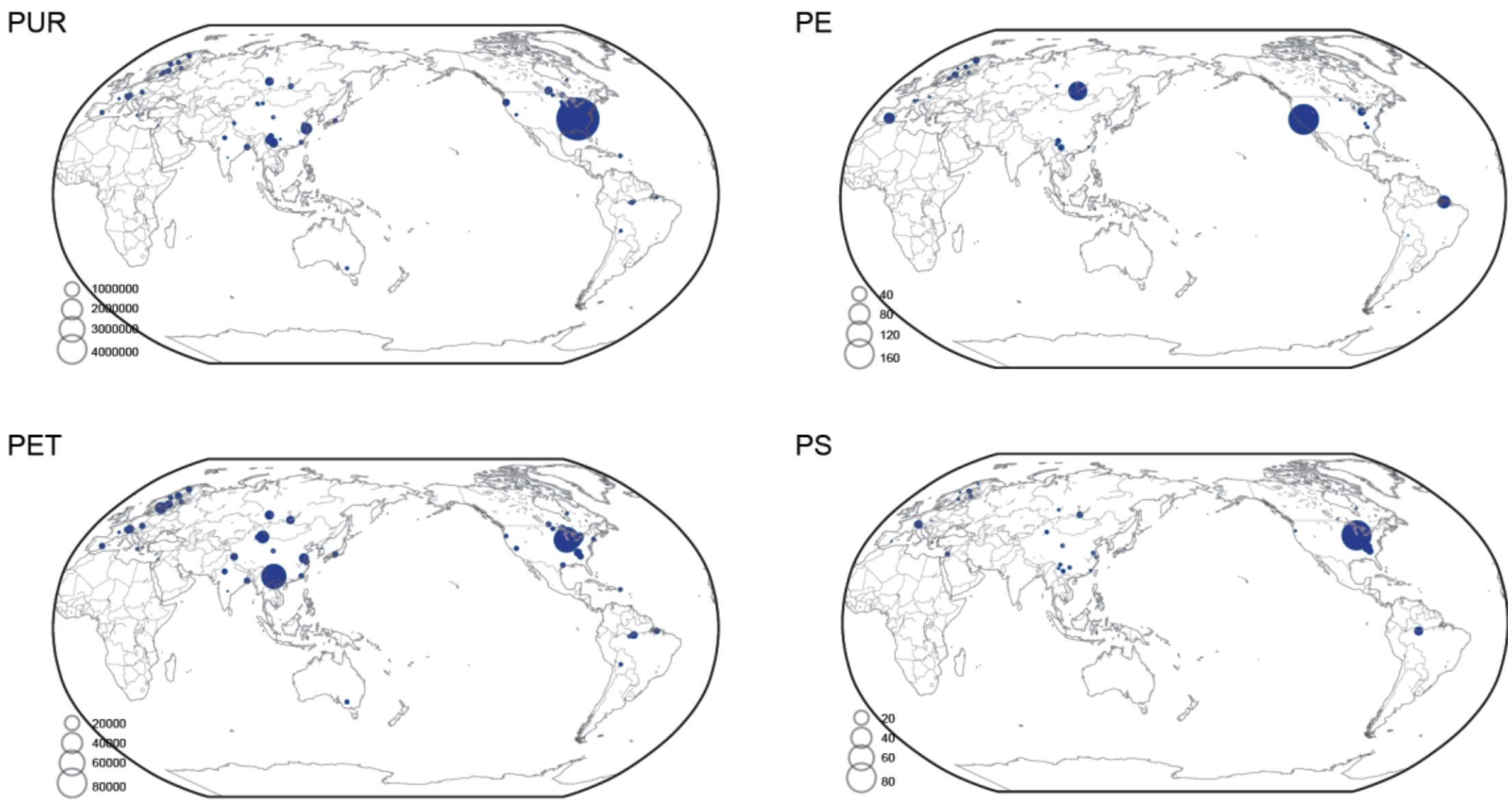

Figure S4. Geographical distribution of plastic-degrading potential of microbes in polyolefin and conventional polyester plastics in lakes The size of the circle indicates the abundance of candidate plastic-degrading enzyme-coding genes. PUR: polyurethane; PET: polyethylene terephthalate; PS: polystyrene; PE: polyethylene.

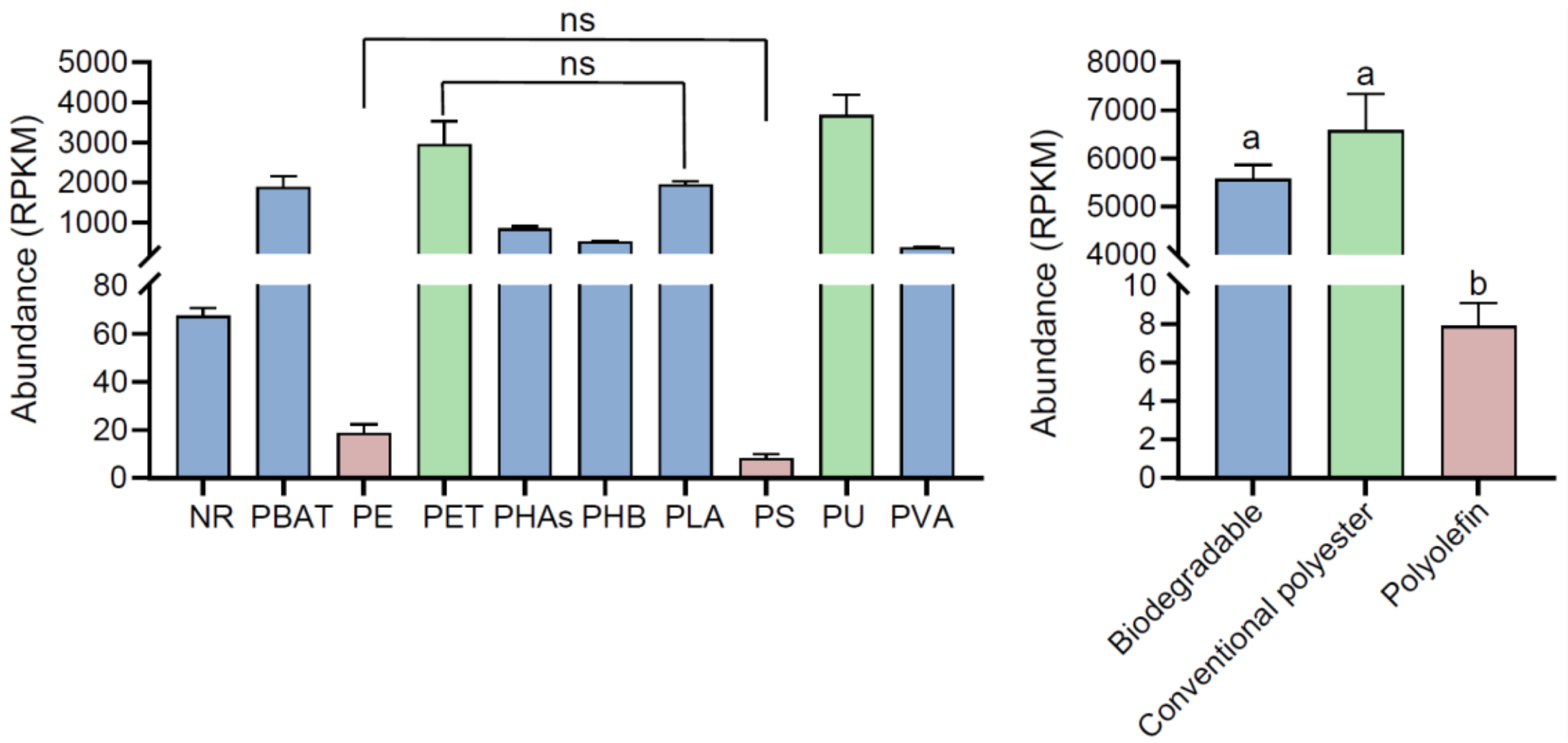

Figure S5. The abundance of plastic-degrading potential in lakes Abundance (RPKM) of various candidate plastic-degrading enzyme-coding genes. Different letters indicate the significant difference among groups (Kruskal-Wallis test, $p < 0.05$). RPKM: Reads Per Kilobase per Million mapped reads. Data are represented as mean ± SEM.

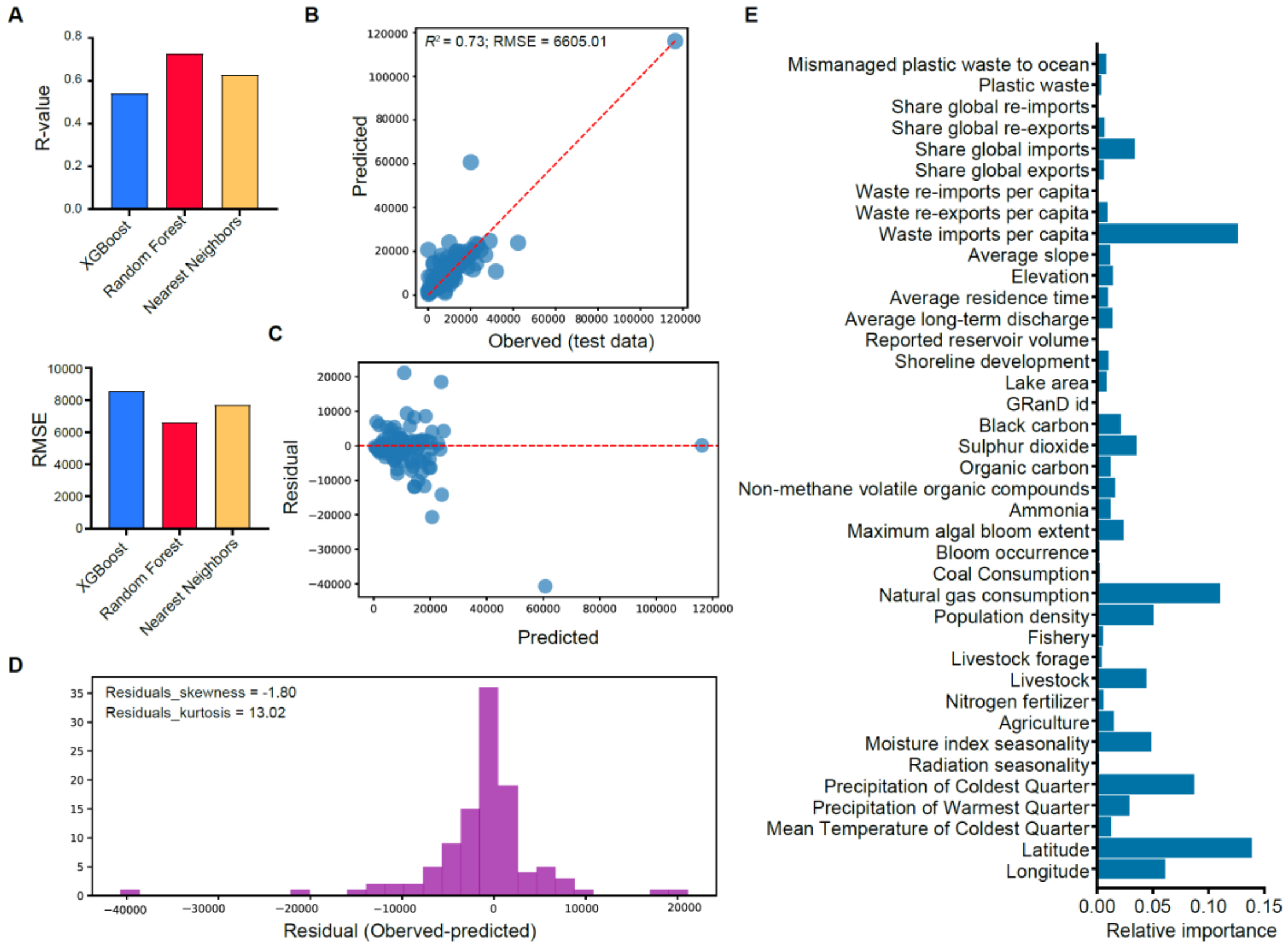

Figure S6. Machine learning prediction of microbial plastic-degradation potential in global lakes (A) Model performance comparison among XGBoost, Random Forest, and K-Nearest Neighbors algorithms based on R^2 and RMSE values. (B) Observed versus predicted plastic-degradation potential in the test dataset using the XGBoost model (R^2 = 0.73; RMSE = 6605.01). (C) Residual plot showing the distribution of prediction errors across samples. (D) Histogram of residuals illustrating skewness and kurtosis values. (E) Relative importance of 39 environmental and anthropogenic predictors in shaping microbial plastic-degradation potential, highlighting the dominant influence of plastic-related and climatic variables.

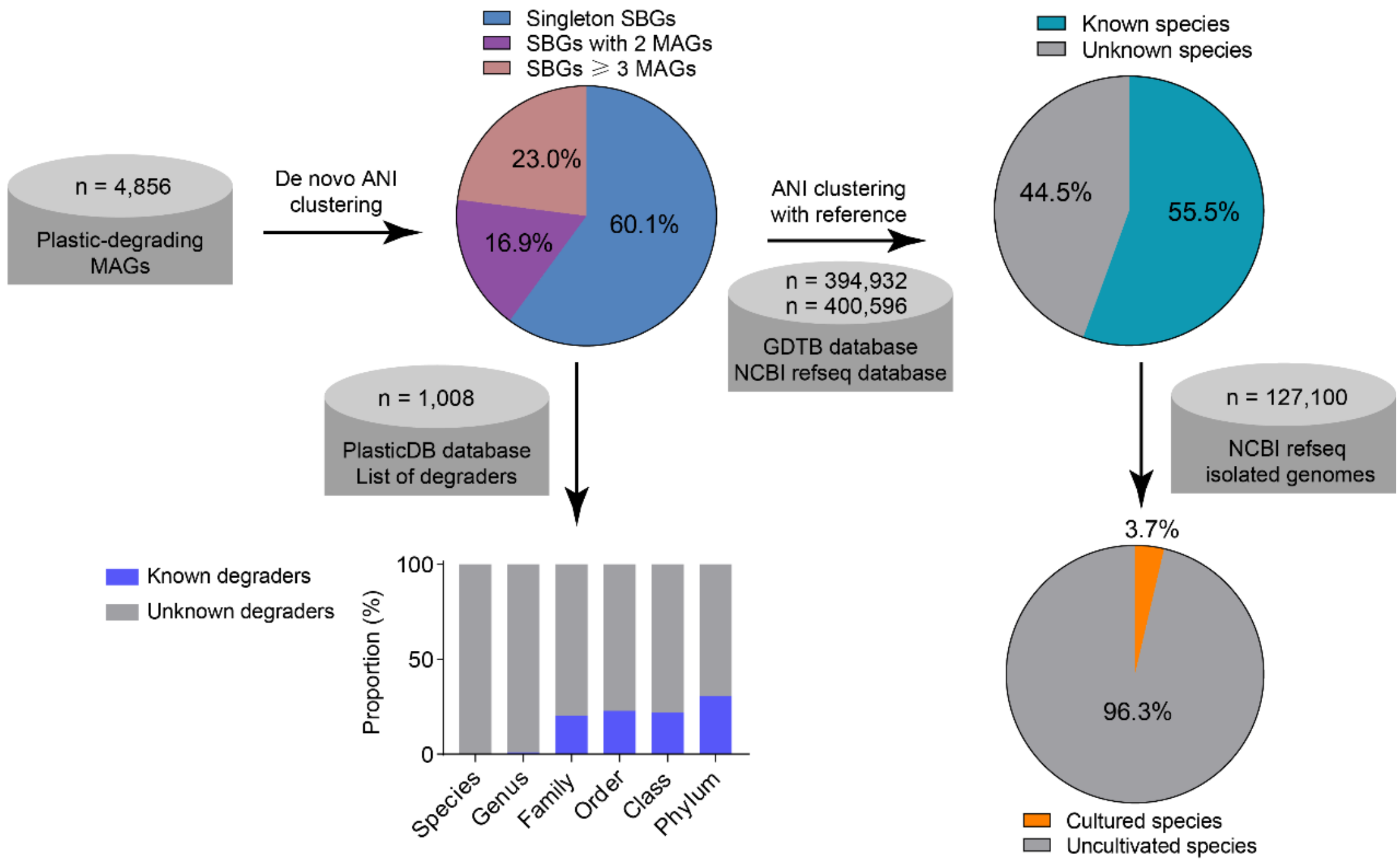

Figure S7. 1,370 genomes (41%) from 4,856 candidate plastic-degrading MAGs were assigned to the plastic degraders We clustered the 4,856 MAGs into 1,370 species-level genome bins (SBGs, denoted as plastic degraders) based on 95% whole-genome average nucleotide identity (ANI).

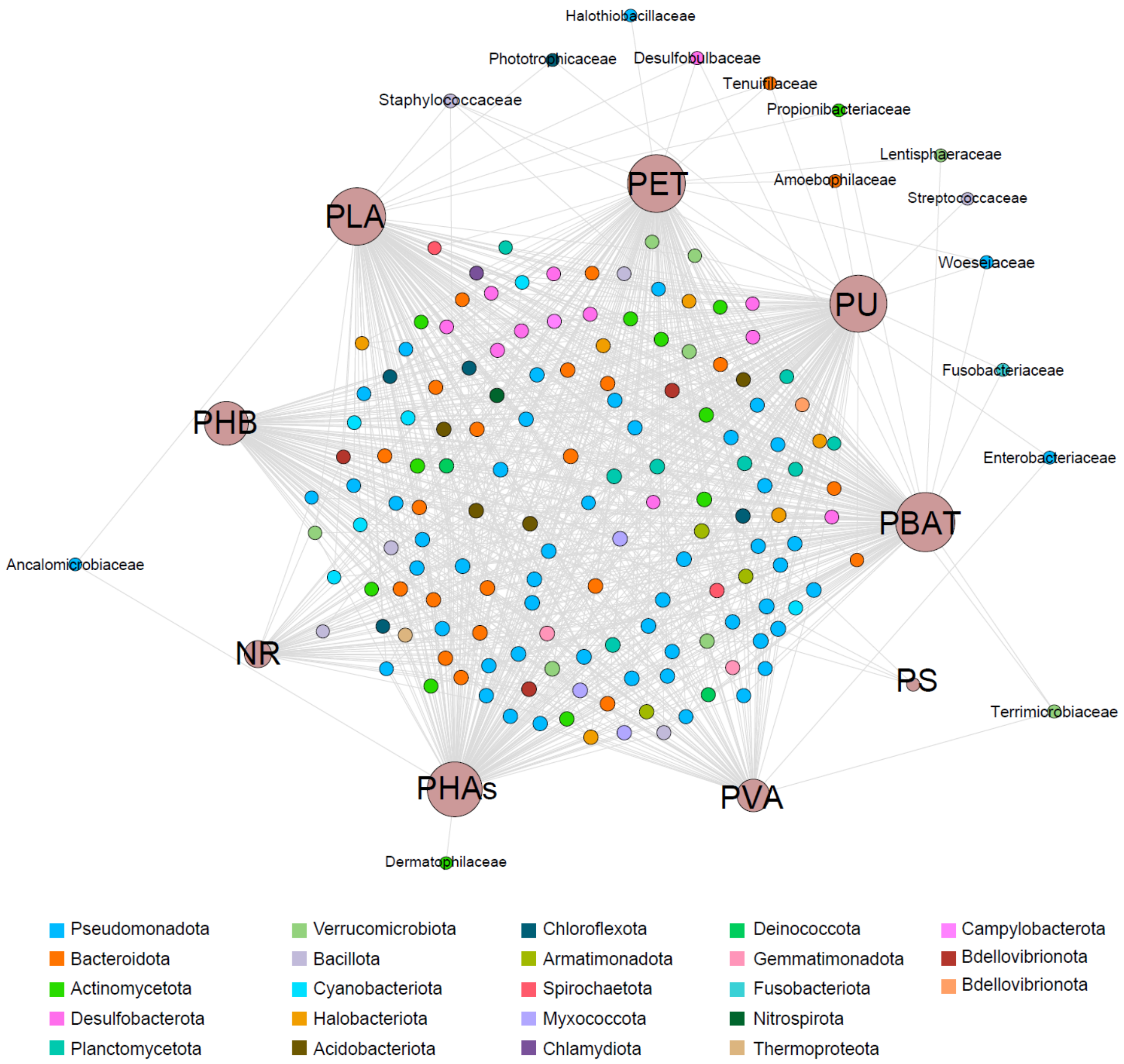

Figure S8. Taxonomic network linking microbial families and plastic types Network visualization shows the associations between candidate plastic-degrading microbial families and ten representative plastic polymers (PLA, PET, PU, PBAT, PHAs, PHB, PVA, PS, NR). Each node represents a microbial family, colored by its corresponding phylum, and edges indicate shared degradation potential inferred from enzyme annotation and co-occurrence patterns. Larger nodes denote plastic types, and node proximity reflects functional similarity in degradation profiles. The network reveals extensive taxonomic overlaps across polymers, suggesting broad enzymatic versatility among plastic-degrading lineages.

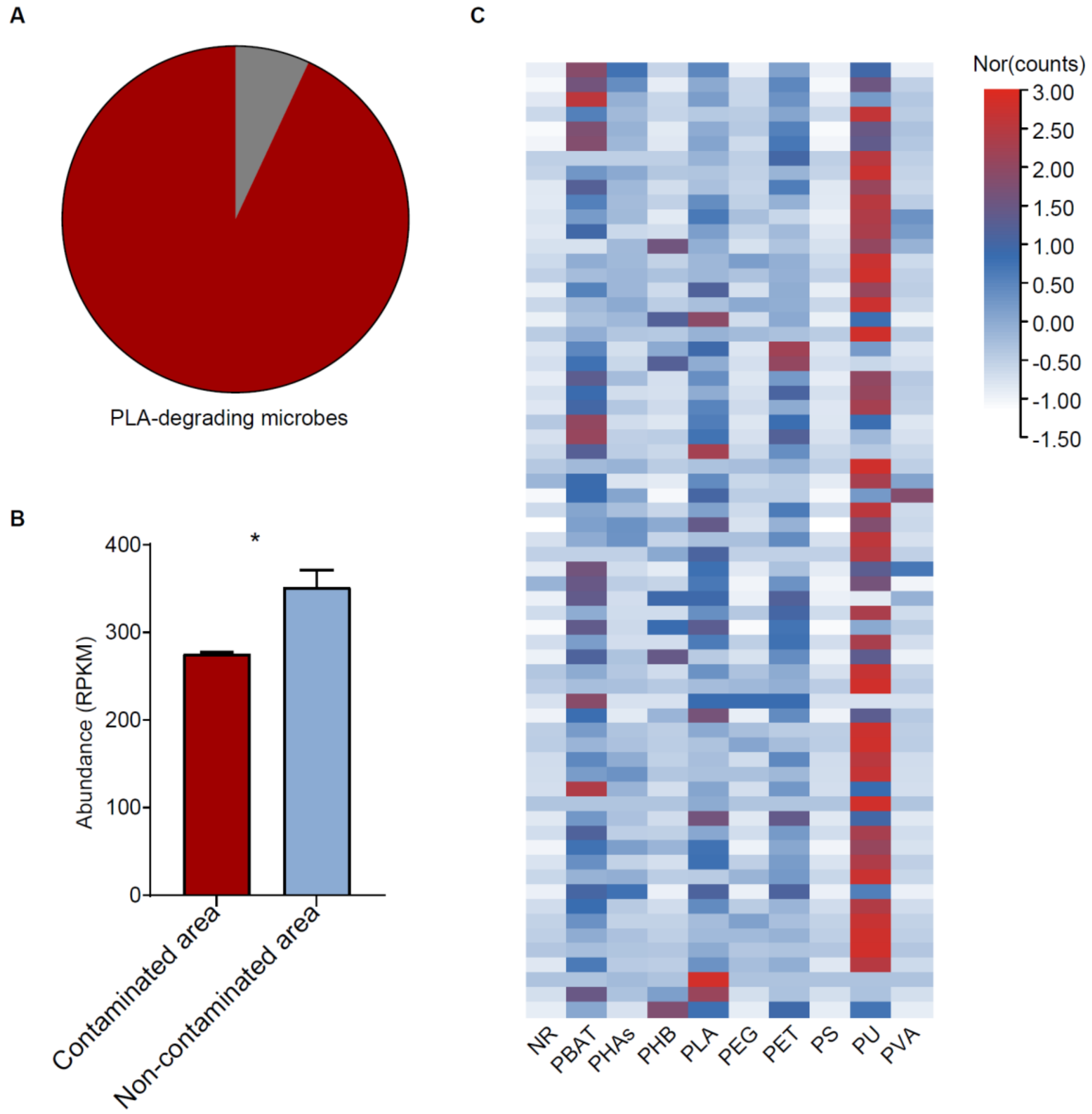

Figure S9. Distribution and functional versatility of candidate PLA-degrading microbes in contaminated versus non-contaminated areas (A) Proportion of candidate PLA-degrading microbes among all candidate degraders detected in the PLA-contaminated site, showing that 93% were PLA degraders. (B) Abundance (RPKM) of candidate PLA-degrading microbes in contaminated and non-contaminated areas, with significantly higher abundance in the contaminated site. Data are represented as mean ± SEM. "*" indicates significance at $P < 0.05$ (one-sided *t*-test). (C) Heatmap of normalized counts of enzymes encoded by candidate PLA-degrading microbes, showing their functional repertoire across different plastic types.

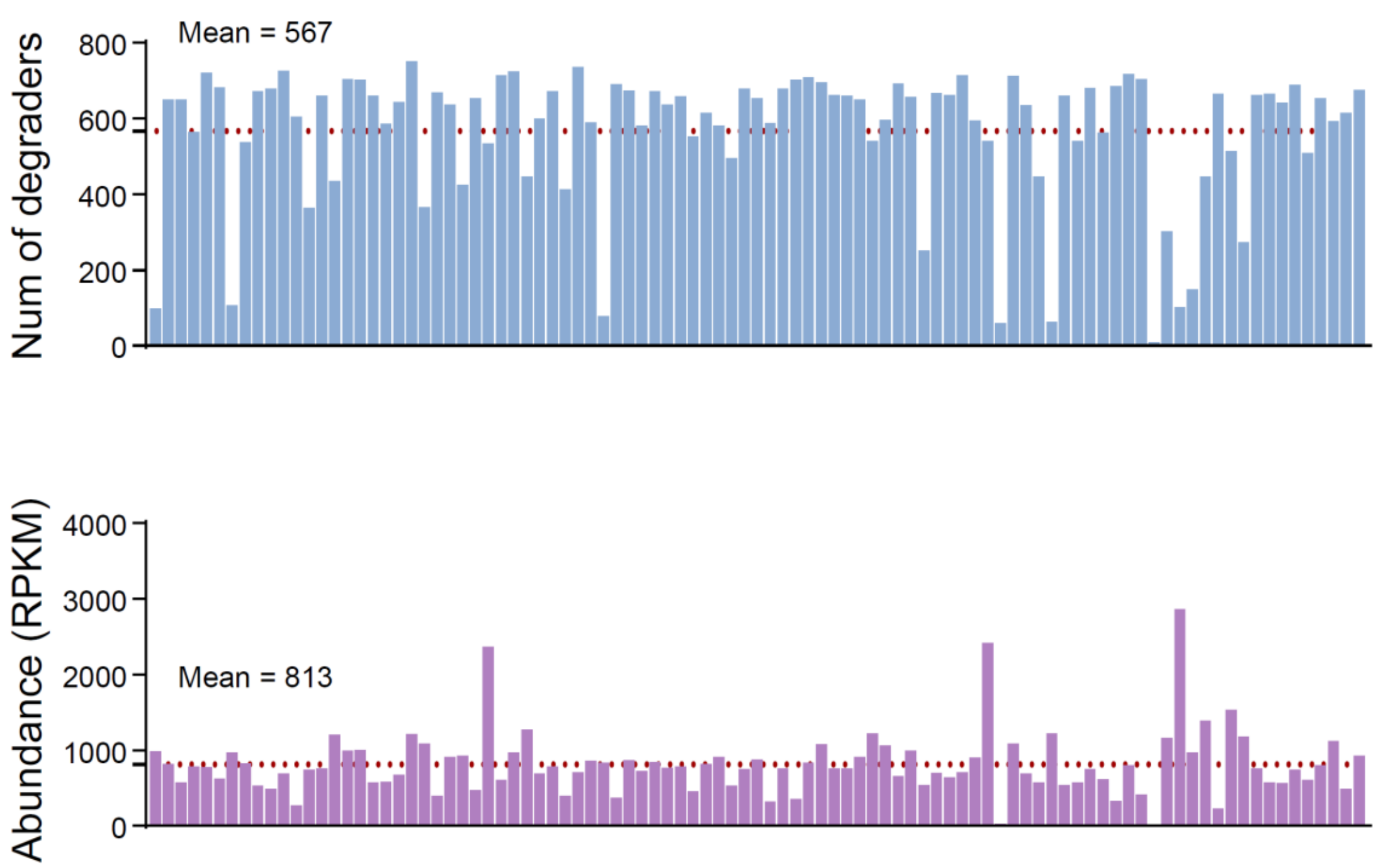

Figure S10. Number and abundance of candidate plastic-degrading bacteria in lake metagenomes We detected an average of 567 candidate plastic-degrading taxa per sample, with a mean abundance of 813 (RPKM).

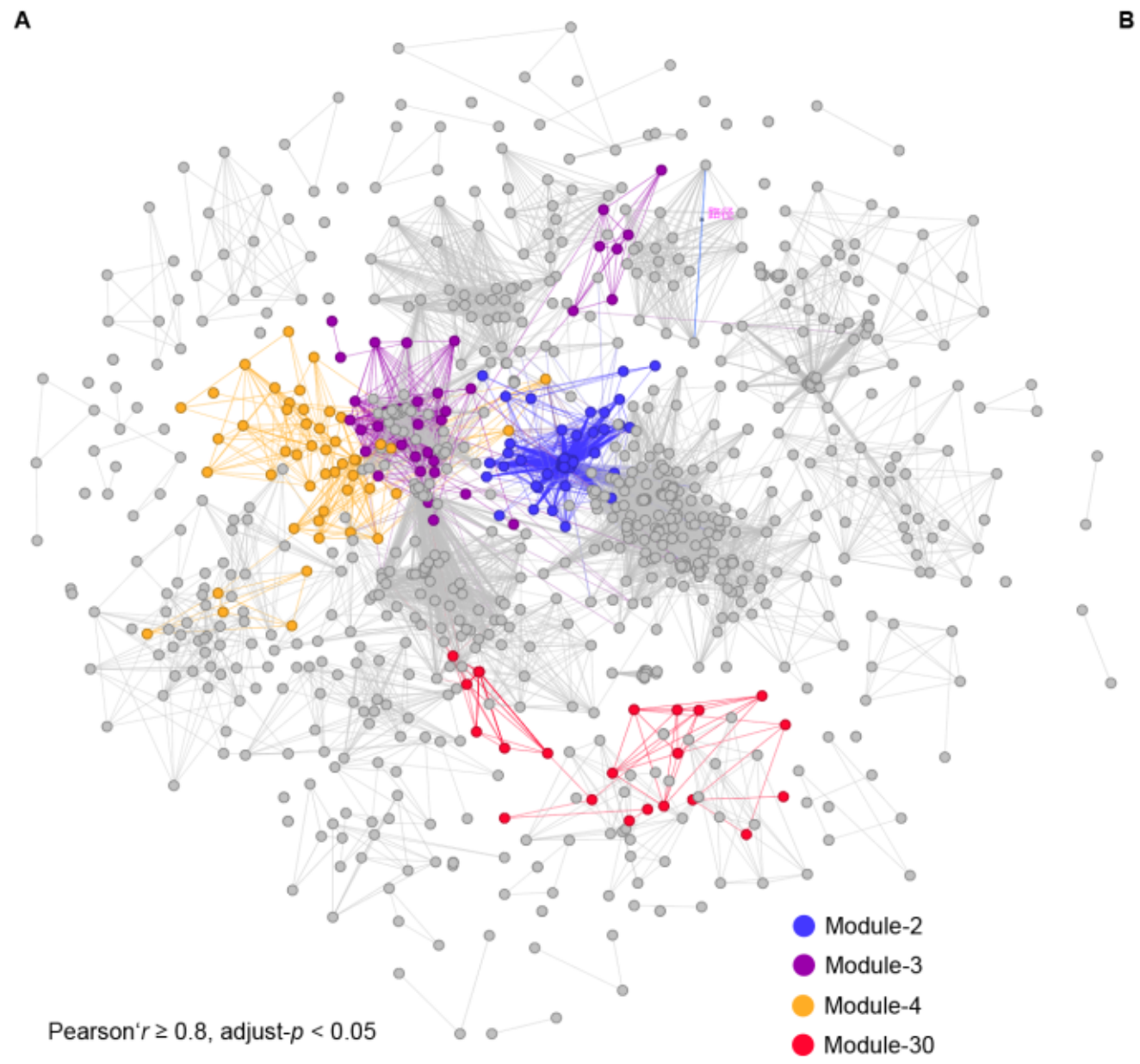

Figure S11. Co-occurrence modules associated with candidate plastic-degrading bacteria in lake metagenomes (A) Network showing microbial taxa significantly correlated with candidate plastic-degrading bacteria (Pearson's $r \geq 0.8$, adjusted $p < 0.05$). Colored nodes represent four major modules. (B) Taxonomic composition of the four modules, highlighting dominant non-degrading taxa potentially cooperating with degraders in nutrient cycling and metabolic interactions.

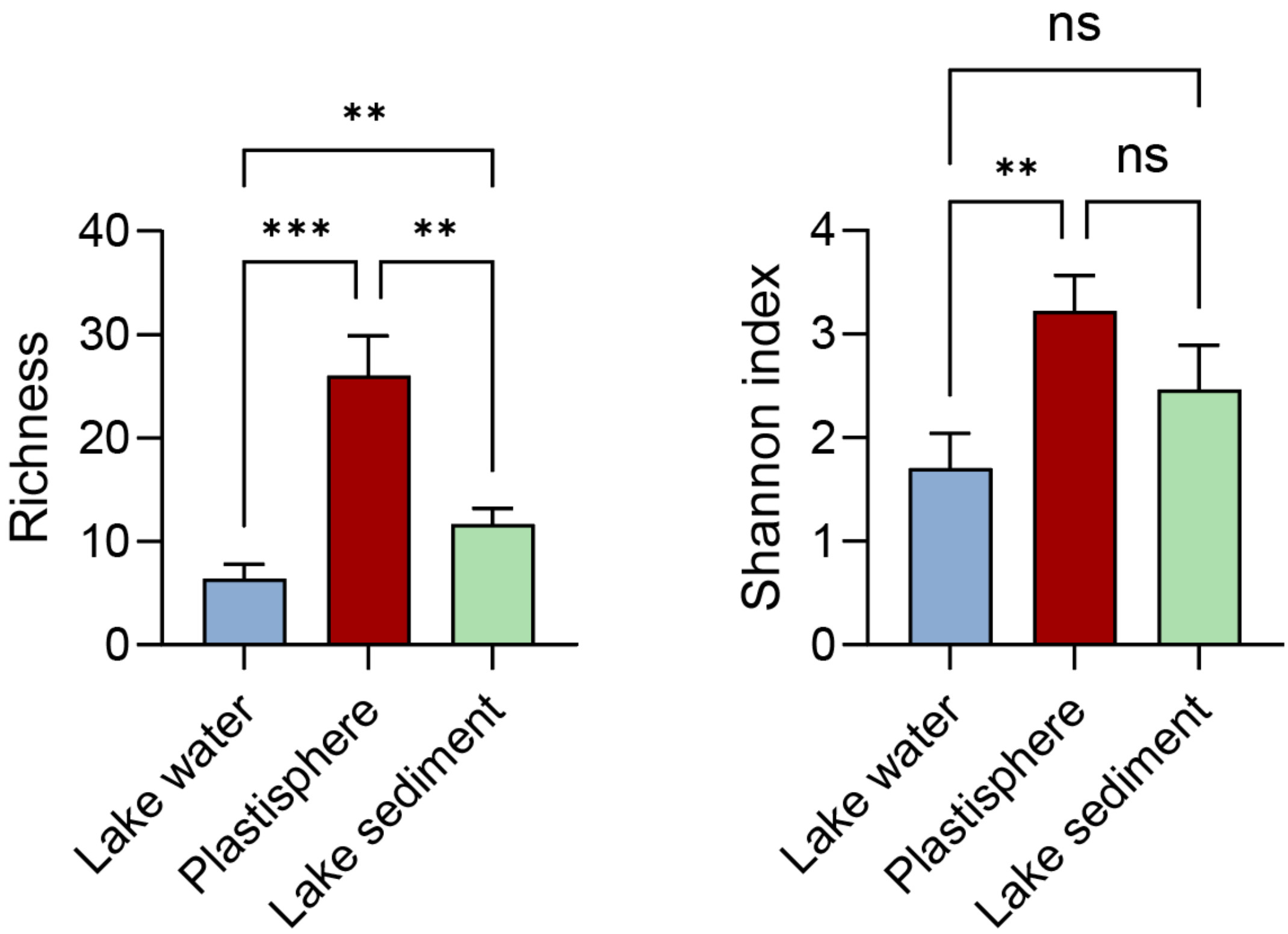

Figure S12. Diversity of candidate plastic-degraders in plateau karst lakes The plastisphere in lakes is a hot zone for enriching and isolating plastic-degrading bacteria. Data are represented as mean ± SEM. "**"and "***" indicate significance at $P < 0.01$ and $P < 0.001$, respectively (one-way ANOVA with Bartlett's test).

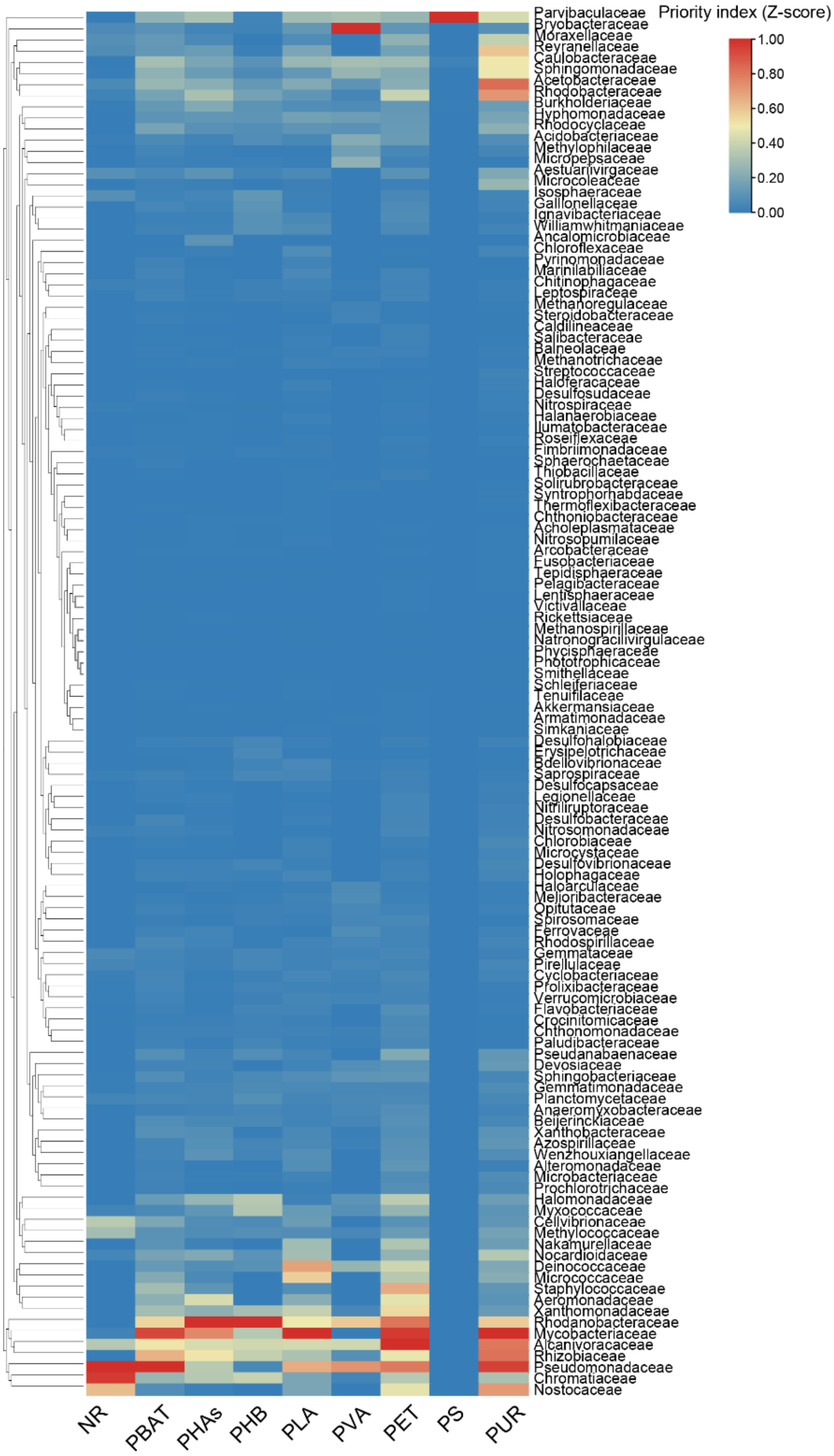

Figure S13. The priority index of candidate degraders in various plastics Color gradient indicates the Z-score of priority index from high (red) to low (blue).

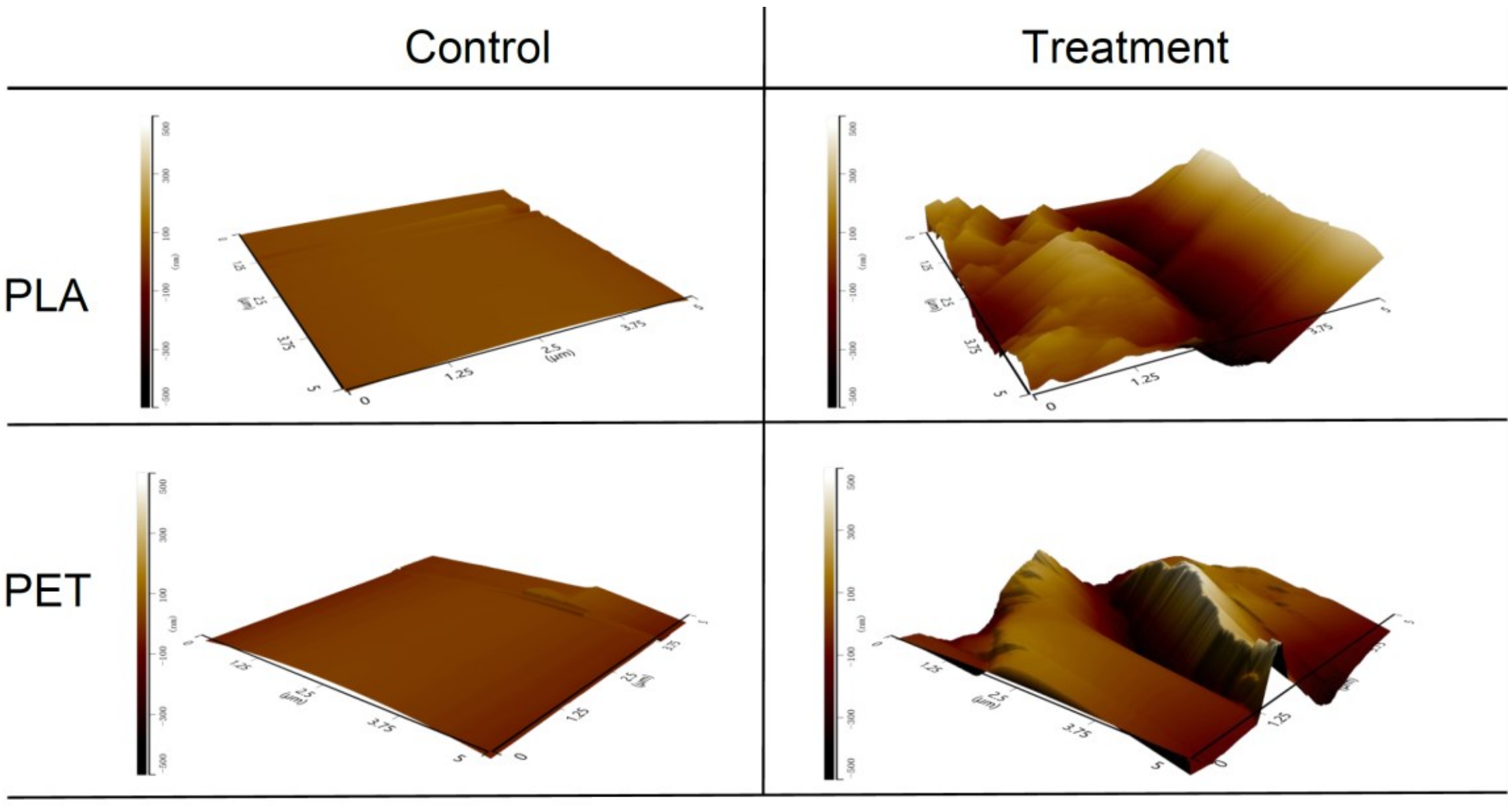

Figure S14. The topography of plastic surface Topographic map of a 5*5 μm microregion shows that the roughness of plastic surface was significantly increased in treatment groups, especially the PLA and PET.

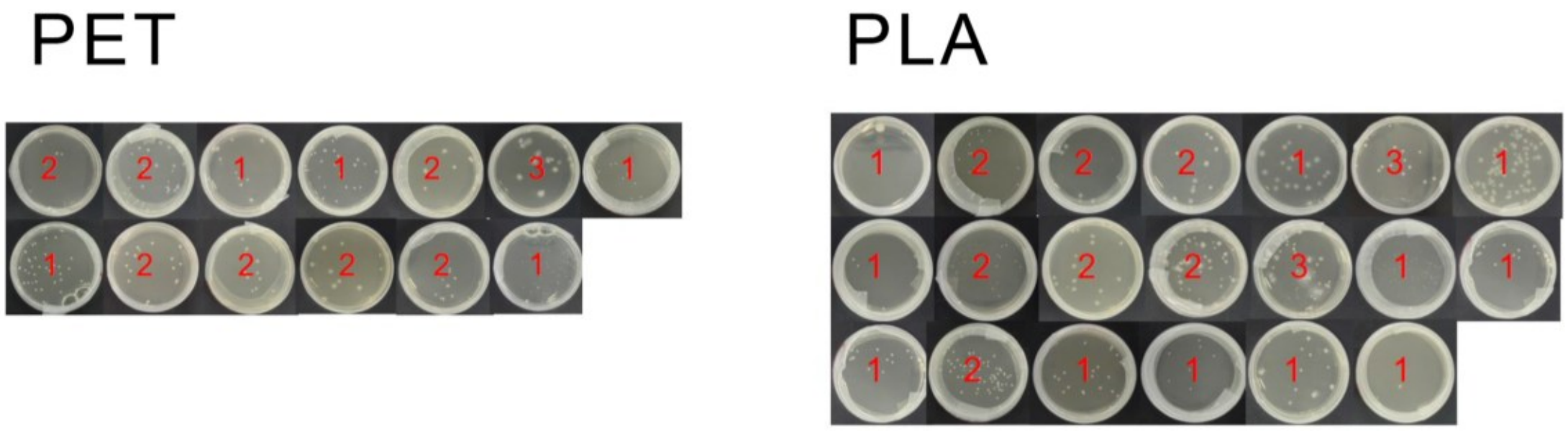

Figure S15. Candidate plastic-degrading bacterial colonies A total of 53 candidate plastic-degrading bacterial colonies were isolated from eight customized media containing PLA and PET particles.

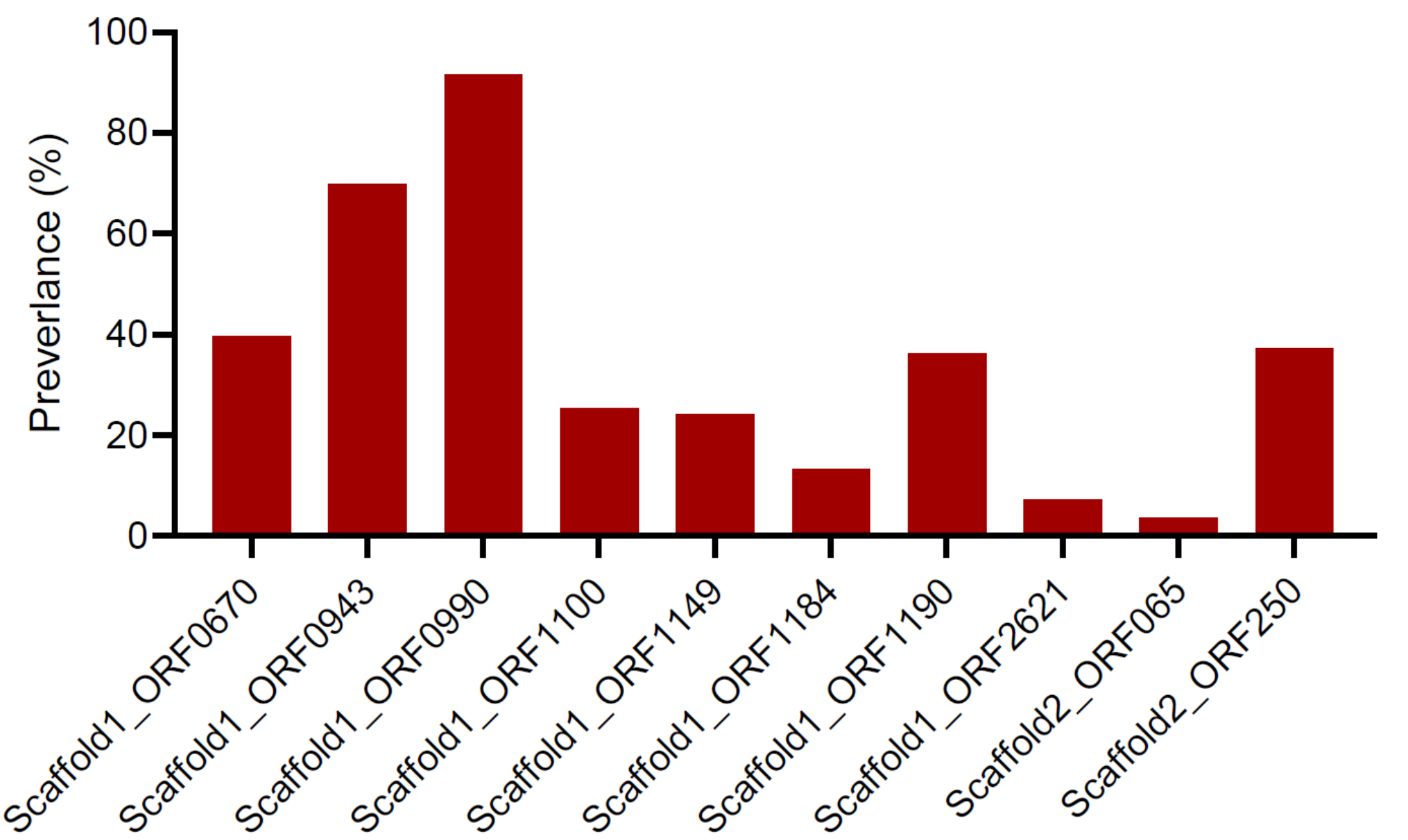

Figure S16. The detection rate of putative plastic-degrading enzymes in the lakes

Metatranscriptomic surveys showed that these putative enzymes were frequently detected, suggesting they are actively expressed in natural environments and thus likely to play functional roles in plastic degradation *in situ*.

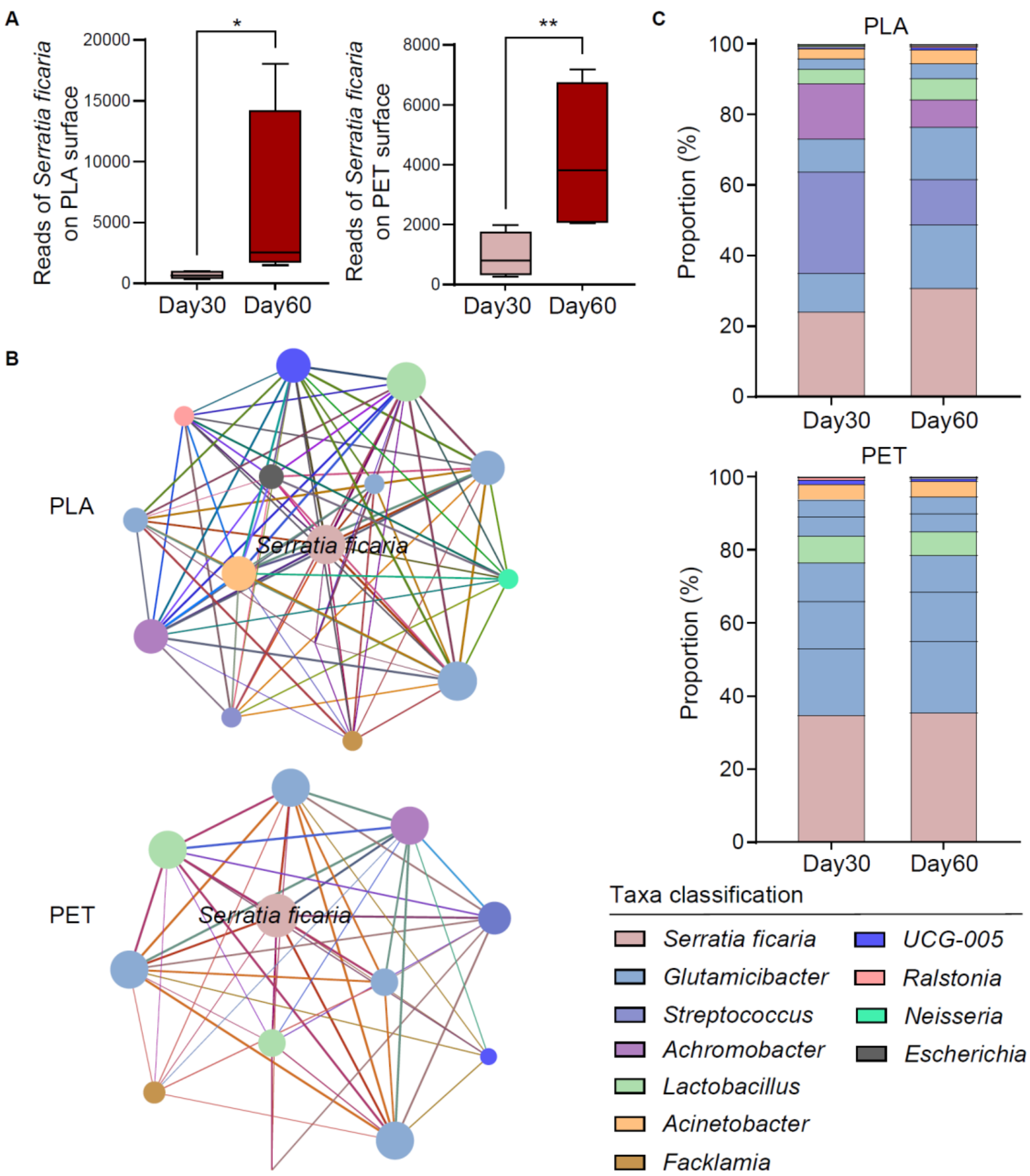

Figure S17. Synergistic microbial interactions during PLA and PET degradation

(A) Temporal enrichment of *Serratia ficaria* on PLA and PET surfaces during 30- and 60-day incubation, showing a significant increase in relative abundance over time. Data are represented as mean ± SEM. "*"and "**" indicate significance at $P < 0.05$ and $P < 0.01$, respectively (one-sided *t*-test). (B) Co-occurrence networks of microbial consortia associated with *S. ficaria* on PLA and PET surfaces. Node size represents relative abundance, and edge color indicates positive correlations among taxa. (C) Community composition at the genus level, showing the increasing dominance of *S. ficaria* and its cooperative taxa (e.g., *Glutamicibacter*, *Achromobacter*, *Ralstonia*, *Lactobacillus*) over time.

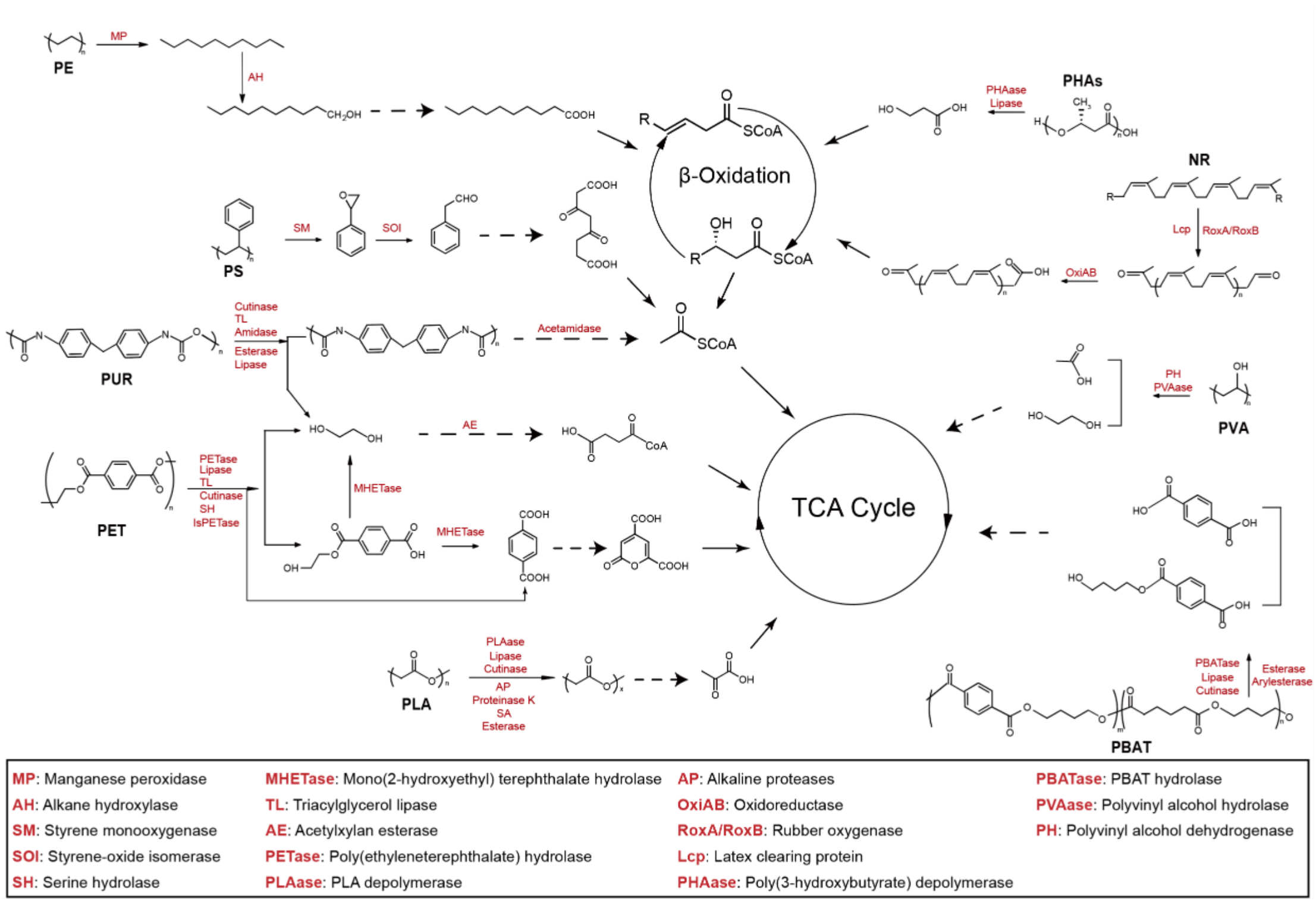

Figure S18. Biodegradation pathways of various plastics PVA, polyvinyl alcohol; PHA, poly-hydroxyalkanoate; PHB, polyhydroxybutyrate; PUR, polyurethane; PET, polyethylene terephthalate; PBAT, polybutylene adipate terephthalate; PS, polystyrene; PLA, polylactic acid; PE, polyethylene; NR, natural rubber. The degradation pathways and enzymes of PS and PE are still in the exploratory stage in the current research, and some enzymes may not be widely verified.

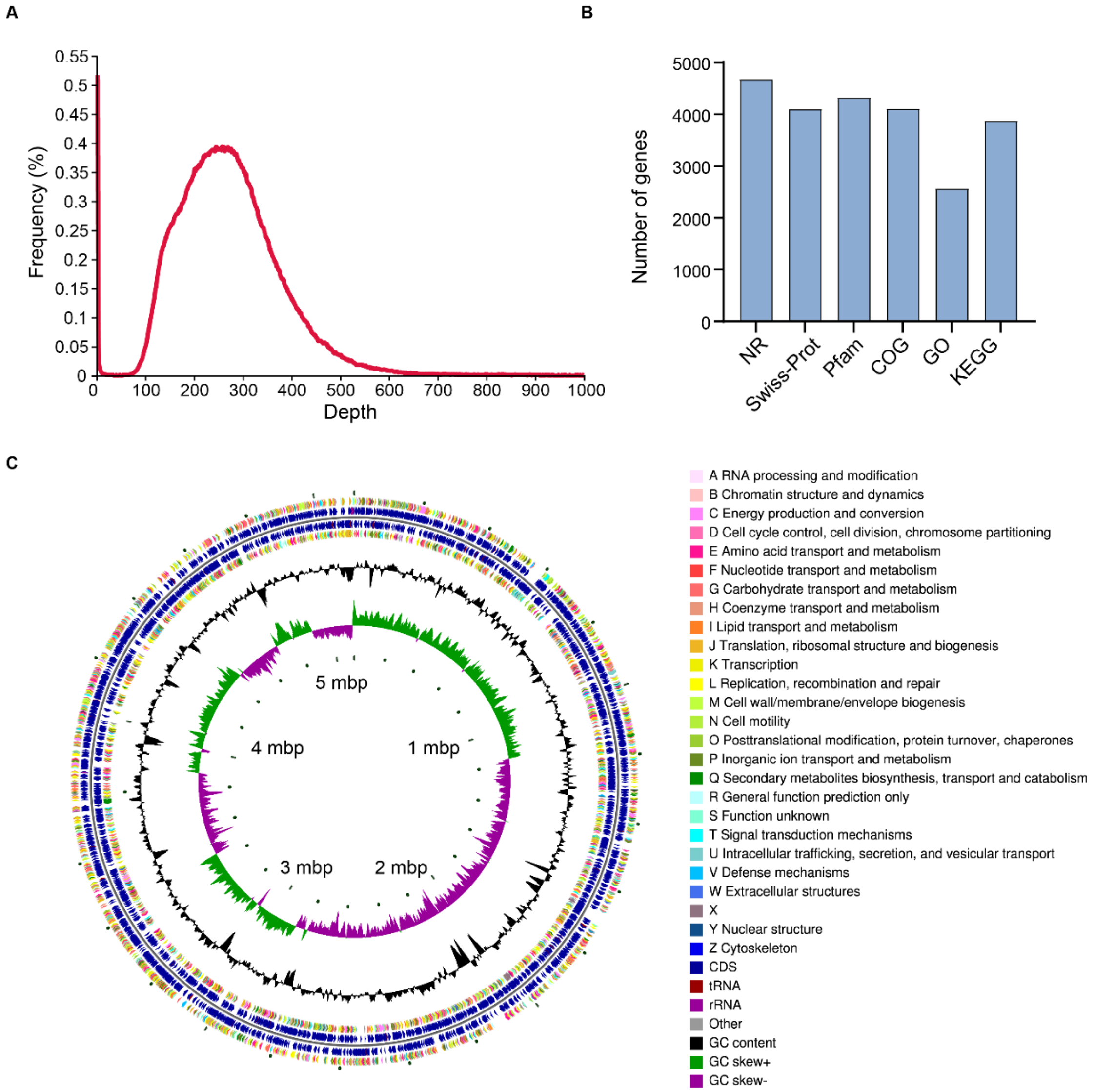

Figure S19. Whole-genome features of *Serratia ficaria* HfyG-1. (A) K-mer frequency distribution showing a peak at ~25× sequencing depth, indicating uniform coverage. (B) Functional annotation counts across databases: NR (highest), Swiss-Prot, COG, GO, and KEGG. (C) Circular genome map (4 Mbp scale) displaying: Outer ring: Functional COG categories (A: RNA processing; S: Unknown function largest segment). Middle rings: Gene features (CDS/tRNA/rRNA). Inner rings: GC content (black) and GC skew (±, blue).

Supplemental references

1. Li D., Liu C. M., Luo R., et al. (2015). MEGAHIT: an ultra-fast single-node solution for large and complex metagenomics assembly via succinct de Bruijn graph. *Bioinformatics* **31**:1674-1676. DOI:10.1093/bioinformatics/btv033
2. Langmead B. and Salzberg S. L. (2012). Fast gapped-read alignment with Bowtie 2. *Nat. Methods* **9**:357-359. DOI:10.1038/nmeth.1923
3. Li H., Handsaker B., Wysoker A. et al. (2009). The Sequence Alignment/Map format and SAMtools. *Bioinformatics* **25**:2078-2079. DOI:10.1093/bioinformatics/btp352
4. Alneberg J., Bjarnason B. S., de Bruijn I. et al. (2014). Binning metagenomic contigs by coverage and composition. *Nat. Methods* **11**:1144-1146. DOI:10.1038/nmeth.3103
5. Wu Y. W., Simmons B. A. and Singer S. W. (2016). MaxBin 2.0: an automated binning algorithm to recover genomes from multiple metagenomic datasets. *Bioinformatics* **32**:605-607. DOI:10.1093/bioinformatics/btv638
6. Kang D. D., Li F., Kirton E. et al. (2019). MetaBAT 2: an adaptive binning algorithm for robust and efficient genome reconstruction from metagenome assemblies. *PeerJ* **7**:e7359. DOI:10.7717/peerj.7359
7. Parks D. H., Imelfort M., Skennerton C. T. et al. (2015). CheckM: assessing the quality of microbial genomes recovered from isolates, single cells, and metagenomes. *Genome Res.* **25**:1043-1055. DOI:10.1101/gr.186072.114
8. Olm M. R., Brown C. T., Brooks B. et al. (2017). dRep: a tool for fast and accurate genomic comparisons that enables improved genome recovery from metagenomes through de-replication. *ISME J.* **11**:2864-2868. DOI:10.1038/ismej.2017.126
9. Aroney S. T. N., Newell R. J. P., Nissen J. N. et al. (2025). CoverM: read alignment statistics for metagenomics. *Bioinformatics* **41**. DOI:10.1093/bioinformatics/btaf147
10. Chan Patricia P., Lin Brian Y., Mak Allysia J. et al. (2021). tRNAscan-SE 2.0: improved detection and functional classification of transfer RNA genes. *Nucleic*

Acids Res. **49**:9077-9096. DOI:10.1093/nar/gkab688

11. Buchholz P. C. F., Feuerriegel G., Zhang H. et al. (2022). Plastics degradation by hydrolytic enzymes: The plastics-active enzymes database-PAZy. *Proteins* **90**:1443-1456. DOI:10.1002/prot.26325
12. Gambarini V., Pantos O., Kingsbury J. M. et al. (2022). PlasticDB: a database of microorganisms and proteins linked to plastic biodegradation. *Database (Oxford)* **2022**. DOI:10.1093/database/baac008
13. Altschul S. F., Gish W., Miller W., et al. (1990). Basic local alignment search tool. *J. Mol. Biol.* **215**:403-410. DOI:10.1016/S0022-2836(05)80360-2
14. Fu L., Niu B., Zhu Z. et al. (2012). CD-HIT: accelerated for clustering the next-generation sequencing data. *Bioinformatics* **28**:3150-3152. DOI:10.1093/bioinformatics/bts565
15. Li W. and Godzik A. (2006). Cd-hit: a fast program for clustering and comparing large sets of protein or nucleotide sequences. *Bioinformatics* **22**:1658-1659. DOI:10.1093/bioinformatics/btl158
16. Katoh K. and Standley D. M. (2016). A simple method to control over-alignment in the MAFFT multiple sequence alignment program. *Bioinformatics* **32**:1933-1942. DOI:10.1093/bioinformatics/btw108
17. Eddy S. R. (1998). Profile hidden Markov models. *Bioinformatics* **14**:755-763. DOI:10.1093/bioinformatics/14.9.755
18. Yu T., Cui H., Li J. C. et al. (2023). Enzyme function prediction using contrastive learning. *Science* **379**:1358-1363. DOI:10.1126/science.adf2465
19. Alcock B. P., Raphenya A. R., Lau T. T. Y. et al. (2020). CARD 2020: antibiotic resistome surveillance with the comprehensive antibiotic resistance database. *Nucleic Acids Res.* **48**:D517-d525. DOI:10.1093/nar/gkz935
20. Buchfink B., Reuter K. and Drost H. G. (2021). Sensitive protein alignments at tree-of-life scale using DIAMOND. *Nat. Methods* **18**:366-368. DOI:10.1038/s41592-021-01101-x
21. Tu Q., Lin L., Cheng L. et al. (2019). NCycDB: a curated integrative database for fast and accurate metagenomic profiling of nitrogen cycling genes.

Bioinformatics **35**:1040-1048. DOI:10.1093/bioinformatics/bty741

22. Yu X., Zhou J., Song W. et al. (2021). SCycDB: A curated functional gene database for metagenomic profiling of sulphur cycling pathways. *Mol. Ecol. Resour.* **21**:924-940. DOI:10.1111/1755-0998.13306

23. Zheng B. J. Zhu Y., Sardans J. et al. (2018). QMEC: a tool for high-throughput quantitative assessment of microbial functional potential in C, N, P, and S biogeochemical cycling. **61**:1451-1462. DOI: 10.1007/s11427-018-9364-7

24. Shi Z., Yin H., Van Nostrand J. D. et al. (2019). Functional Gene Array-Based Ultrasensitive and Quantitative Detection of Microbial Populations in Complex Communities. *mSystems* **4**. DOI:10.1128/mSystems.00296-19

25. Nadell C. D., Drescher K. and Foster K. R. (2016). Spatial structure, cooperation and competition in biofilms. *Nat. Rev. Microbiol.* **14**:589-600. DOI:10.1038/nrmicro.2016.84

26. Du Toit A. (2018). Increasing virulence factors. *Nat. Rev. Microbiol.* **16**:658-658. DOI:10.1038/s41579-018-0091-3

27. Zhang Z., Zhang Q., Wang T. et al. (2022). Assessment of global health risk of antibiotic resistance genes. *Nat. Commun.* **13**:1553. DOI:10.1038/s41467-022-29283-8

28. Atlas R. (2005). Handbook of media for environmental microbiology, second edition. Q. Rev. Biol. 10.1201/9781420037487.

29. Cross K. L., Campbell J. H., Balachandran M. et al. (2019). Targeted isolation and cultivation of uncultivated bacteria by reverse genomics. *Nat. Biotechnol.* **37**:1314-1321. DOI:10.1038/s41587-019-0260-6

30. Chen S., Zhou Y., Chen Y. et al. (2018). fastp: an ultra-fast all-in-one FASTQ preprocessor. *Bioinformatics* **34**:i884-i890. DOI:10.1093/bioinformatics/bty560

31. Seppey M., Manni M. and Zdobnov E. M. (2019). BUSCO: Assessing Genome Assembly and Annotation Completeness. *Methods Mol. Biol.* **1962**:227-245. DOI:10.1007/978-1-4939-9173-0_14

32. Iñiguez M. E., Conesa J. A. and Fullana A. (2018). Recyclability of four types

of plastics exposed to UV irradiation in a marine environment. *Waste Manag.* **79**:339-345. DOI:10.1016/j.wasman.2018.08.006

33. Asnicar F., Thomas A. M., Beghini F. et al. (2020). Precise phylogenetic analysis of microbial isolates and genomes from metagenomes using PhyloPhlAn 3.0. *Nat. Commun.* **11**:2500. DOI:10.1038/s41467-020-16366-7

34. Price M. N., Dehal P. S. and Arkin A. P. (2010). FastTree 2–Approximately Maximum-Likelihood Trees for Large Alignments. *PLoS One* **5**:e9490. DOI:10.1371/journal.pone.0009490

35. Stamatakis A. (2014). RAxML version 8: a tool for phylogenetic analysis and post-analysis of large phylogenies. *Bioinformatics* **30**:1312-1313. DOI:10.1093/bioinformatics/btu033

36. Letunic I. and Bork P. (2021). Interactive Tree Of Life (iTOL) v5: an online tool for phylogenetic tree display and annotation. *Nucleic Acids Res.* **49**:W293-W296. DOI:10.1093/nar/gkab301
